# Causes of death across the life course in the Association of Southeast Asian Nations (ASEAN): a systematic analysis for the Global Burden of Disease Study 2023

**DOI:** 10.1016/S2468-2667(26)00159-3

**Published:** 2026-09-22

**Authors:** GBD 2023 ASEAN Causes of Death Collaborators, Taishun Li, Periklis Charalampous, Stephanie C C van der Lubbe, Yawen Zhou, Simon I Hay, Jeofrey Bautista Abalos, Edimansyah Abdin, Hanif Abdul Rahman, Asnawi Abdullah, Abdulrakib Abdulrahim, Michael R M Abrigo, Wirawan Adikusuma, Tawseef Ahmad, Sima Ahmadpour, Mohamed Mustaf Ahmed, Budi Aji, Mohammed Alfaqeeh, Khaidar Ali, Syed Mohamed Aljunid, Joseph Uy Almazan, Ali Salman Al-Shami, Junaid Amin, Roshan A Ananda, Chin-Siang Ang, Nguyen Hoang Anh, Sumadi Lukman Anwar, Irfan Ardani, Rizal Arifin, Keivan Armani, Mohammad Ashfaq, Muhammad Shahzad Aslam, Yuni Asri, Zaw Zaw Aung, Zulfa Ayuningsih, Fitriyono Ayustaningwarno, Abul Kalam Azad, Mohd Yusmaidie Aziz, Ahmed Bali, Muhammad Irfan Bashir, Bagas Suryo Bintoro, Ahmad Naoras Bitar, Ai Cao My Bui, Asmat Burhan, Muhammad Daoud Butt, Dimples Acquiatan Camporedondo, Yu Cao, Hana Chen, Nicholas WS Chew, Ruixin Chi, Bryan Chong, Dennis Chong, Karleen Chong, Christina Malini Christopher, Dinh-Toi Chu, Mayank Dalakoti, Pojsakorn Danpanichkul, Bryan Gervais de Liyis, Edward Christopher Dee, Sadia Din, Mai Ngoc Do, Thao Huynh Phuong Do, Minh Cuong Duong, Angel Belle Cheng Dy, Ferry Efendi, Ekawati Ekawati, Muhammad Esa, Maryam Farooqui, Nelsensius Klau Fauk, Neeraj Kumar Fuloria, Shivkanya Fuloria, Francis Sarial Ganancial, Fernando Jr Barroga Garcia, Adrian Eleazar Go, Bey Hing Goh, Ernesto Ramos Gregorio, Nguyen Hai Nam, Harapan Harapan, Arief Hargono, Andy Martahan Andreas Hariandja, Faizul Hasan, Zaw Myo Hein, Rohman Hikmat, Aaron Kwun Hang Ho, Tinh Ho Huu, Mehdi Hosseinzadeh, Mega Hasanul Huda, Fadly Husain, Mohd Salami Ibrahim, Umar Idris Ibrahim, Korakrit Imwattana, Lalu Muhammad Irham, Fahrul Islam, Jazlan Jamaluddin, Roland Dominic G Jamora, Hanish Singh Jayasingh Chellammal, Yovanthi Anurangi Jayasinghe, Jost B Jonas, Muhammad Khan, Parisa Khoshvaght, Siti Khuzaiyah, Ko Ko, Soewarta Kosen, Chong-Han Kua, Garry Kuan, Yee Cheng Kueh, Maria Dyah Kurniasari, Dea Anita Ariani Kurniasih, Asep Kusnali, Aria Kusuma, Dian Kusuma, Daphne Teck Ching Lai, Agung Dwi Laksono, Tri Laksono, Jerrald Lau, Bao Huy Le, Thao Thi Thu Le, Hoi Leong Lee, Shaun Wen Huey Lee, Elvynna Leong, Mohammad Farris Iman Leong Bin Abdullah, Retno Lestari, Mei Fong Liew, Lee-Ling Lim, Donglin Liu, Xiao Liu, Arianna Maever Loreche, Hung N Luu, Kaung Suu Lwin, Zheng Feei Ma, Melani Ratih Mahanani, Roy Rillera Marzo, Richard James Maude, Muhammad Miftahussurur, Muhammad Agus Mikrajab, Long Chiau Ming, Maziar Moradi-Lakeh, Amartya Mukhopadhyay, Lutfi Muzaqi, Kiran P Nagdeo, Firzan Nainu, Tam Thi Ngo, Cao Duy Nguyen, Ha Thu Nguyen, Hien Thu Nguyen, Hien Thi Nguyen, Long Hoang Nguyen, Minh Thien Nguyen, Minh-Ha Nguyen, Nghia Phu Nguyen, Thanh Van Nguyen, Thinh Gia Nguyen, Luong Nguyen Thanh, Cham Thi Nguyen Thi, Ricvan Dana Nindrea, Mohammed Noushad, Nurfatimah Nurfatimah, Ahmad Dzulfikri Nurhan, Sok King Ong, Sandersan Onie, Zhinya Kawa Othman, Helena Ullyartha Pangaribuan, Santa Maria Pangaribuan, Bunga A Paramashanti, Veincent Christian Filipino Pepito, Tung Thanh Pham, Dimitri Poddighe, Kian Keong Poh, Thantrira Porntaveetus, Ragil Angga Prastiya, Yusuf Alif Pratama, Hery Purnobasuki, Nuzul Qur'aniati, Ali Qureshi, Basuki Rachmat, Asad Ur Rahman, Vignesh Ramachandran, Kadar Ramadhan, Sheena Ramazanu, Ateeq Rehman, Yohanes Andy Rias, Erna Rochmawati, Alina Rodriguez, Iftitakhur Rohmah, Thanakorn Rojanathagoon, Chanapong Rojanaworarit, Debby Syahru Romadlon, Betty Roosihermiatie, Bedanta Roy, Suebsarn Ruksakulpiwat, Ahmad Sahban Rafsanjani, Shuaibu Musa Saidu, Yoseph Leonardo Samodra, Lintang Dian Saraswati, Shilpa Sarawagi, Ni Putu Wulan Purnama Sari, Mohammad-Ali Sarvghadi, Tual Sawn Khai, Abrar Mohammad Sayaf, KT Matthew Seah, Zhi Huang See, Siddharthan Selvaraj, Shazlin Shaharudin, JiaHao Shi, Ching-Hui Sia, Robert Sinto, Chandrashekhar T Sreeramareddy, Vetriselvan Subramaniyan, Chee Fai Sui, Desy Sulistiyorini, Henry Surendra, Suryanti Suryanti, Herry Susanto, Hani Susianti, Tri Nugraha Susilawati, Dayinta Annisa Syaiful, Balamurugan Tagiisuran, Ching Siang Tan, Kit-Aun Tan, Ekamol Tantisattamo, Ingan Ukur Tarigan, Andrew W Taylor-Robinson, Van Chi Thai, Lakshmi Thangavelu, Hein Thu, Jansje Henny Vera Ticoalu, Santo Imanuel Tonapa, Nam Minh-phuong Tran, Nam Hoang Tran, Quynh Thuy Huong Tran, Thang Huu Tran, Thi Thuy Vy Tran, Nguyen Tran Minh Duc, Indang Trihandini, Quynh Xuan Nguyen Truong, Abdul Rohim Tualeka, Sok Cin Tye, Josefien van Olmen, Narayanaswamy Venketasubramanian, Nguyen-Kieu Viet-Nhi, Tuan Vinh, Lan Thi Hoang Vu, Linh Vu, Thinh Toan Vu, Ali Wagdy, Mugi Wahidin, Tati Suryati Warouw, Apichai Wattanapisit, Taweewat Wiangkham, Yohanes Cakrapradipta Wibowo, Akhmad Fajri Widodo, Angga Wilandika, Sri Winarni, Utoomporn Wongsin, Mun Fei Yam, Kwang Mo Yang, Siyan Yi, Mustafa Younis, Riky Tri Yunardi, Emilia Zainal Abidin, Lilik Zuhriyah, Mohsen Naghavi, Marie Ng, Cuilin Zhang

**Affiliations:** A Nanjing Drum Tower Hospital, Affiliated Hospital of Medical School, Nanjing University, Nanjing, China; B Global Center for Asian Women's Health (GloW), Yong Loo Lin School of Medicine, National University of Singapore, Singapore; C Yong Loo Lin School of Medicine, National University of Singapore, Singapore, Singapore; D Department of Public Health, Erasmus University Medical Center, Rotterdam, Netherlands; E NUS-IHME Global Burden of Disease Research Centre, Yong Loo Lin School of Medicine, National University of Singapore, Singapore, Singapore; F Institute for Health Metrics and Evaluation, University of Washington, Seattle, WA, USA; G Department of Health Metrics Sciences, School of Medicine, University of Washington, Seattle, WA, USA; H University of Eastern Pangasinan, Binalonan, Philippines; I Research Division, Institute of Mental Health, Singapore, Singapore; J Saw Swee Hock School of Public Health, National University of Singapore, Singapore, Singapore; K Institute of Health Sciences, University of Brunei Darussalam, Gadong, Brunei; L National Health Policy Agency, Ministry of Health, Jakarta, Indonesia; M Universitas Muhammadiyah Aceh, Banda Aceh, Indonesia; N Department of Medical Microbiology, Universiti Putra Malaysia, Serdang, Malaysia; O Philippine Institute for Development Studies, Quezon City, Philippines; P Research Center for Computing, National Research and Innovation Agency (BRIN), Cibinong, Indonesia; Q Department of Clinical Pharmacy, Prince of Songkla University, Hat-Yai, Thailand; R Department of Pharmacy, COMSATS Institute of Information Technology, Abbottabad, Pakistan; S Department of Information System, Universiti Tunku Abdul Rahman, Kampar, Malaysia; T Institute for Global Health, SIMAD University, Mogadishu, Somalia; U Faculty of Medicine and Public Health, Jenderal Soedirman University, Purwokerto, Indonesia; V Department of Pharmacology and Clinical Pharmacy, Padjadjaran University, Bandung, Indonesia; W Department of Public Health, Jenderal Soedirman University, Purwokerto, Indonesia; X Center of Sustainable Development Goals, Jenderal Soedirman University, Purwokerto, Indonesia; Y Department of Public Health and Community Medicine, International Medical University, Kuala Lumpur, Malaysia; Z International Centre for Casemix and Clinical Coding, National University of Malaysia, Bandar Tun Razak, Malaysia; AA Department of Medicine, Nazarbayev University, Astana, Kazakhstan; AB Department of Pharmacology, Sana'a University, Sanaa, Yemen; AC Department of Basic Medical Sciences, Universiti Malaysia Sarawak, Kota Samarahan, Malaysia; AD Department of Physiotherapy, University of Hail, Hail, Saudi Arabia; AE Department of Orthopaedic Rehabilitation, University of Science Malaysia, Kubang Kerian, Malaysia; AF Department of General Medicine, Eastern Health, Box Hill, VIC, Australia; AG Department of Primary Care and Public Health, Imperial College London, London, England; AH World Health Organization for Digital Health and Health Education, Nanyang Technological University, Singapore, Singapore; AI Department of General Medicine, Thai Binh University of Medicine and Pharmacy in Vietnam, Thai Binh City, Viet Nam; AJ Ophthalmology Department, Hoa Binh General Hospital, Hoa Binh City, Viet Nam; AK Department of Surgery, Gadjah Mada University, Yogyakarta, Indonesia; AL Research Center for Public Health and Nutrition, National Research and Innovation Agency (BRIN), Bogor, Indonesia; AM Faculty of Engineering, Universitas Muhammadiyah Ponorogo, Ponorogo, Indonesia; AN School of Public Health, Imperial College London, London, England; AO Faculty of Pharmaceutical Sciences, UCSI University, Kuala Lumpur, Malaysia; AP Research & Development Cell, Woxsen University, Hyderabad, India; AQ Faculty of Engineering, Chulalongkorn University, Bangkok, Thailand; AR School of Traditional Chinese Medicine, Xiamen University Malaysia, Sepang, Malaysia; AS Nursing Department, Institute of Technology and Health Science RS Dr Soepraoen, Malang, Indonesia; AT Faculty of Health Science, Institute of Technology and Health Science RS Dr Soepraoen, Malang, Indonesia; AU Department of Health Outcome and Policy Evaluation, Thailand National Health Foundation, Bangkok, Thailand; AV Department of Nutrition, Universitas Islam Negeri Walisongo, Semarang, Indonesia; AW Nutrition Science Department, Diponegoro University, Semarang, Indonesia; AX Center of Nutrition Research (CENURE), Diponegoro University, Semarang, Indonesia; AY Department of Pharmacy, Universiti Antarabangsa MAIWP, Kuala Lumpur, Malaysia; AZ Advanced Medical & Dental Institute, Universiti Sains Malaysia, Penang, Malaysia; BA Institute of Research and Development, Duy Tan University, Da Nang, Viet Nam; BB Jadara Research Center, Jadara University, Irbid, Jordan; BC Faculty of Pharmacy, Universiti Sultan Zainal Abidin, Terengganu, Malaysia; BD Department of Health Behaviour, Environment and Social Medicine, Universitas Gadjah Mada, Sleman, Indonesia; BE Center of Health and Behavior and Promotion, Universitas Gadjah Mada, Sleman, Indonesia; BF Department of Clinical Pharmacy, Universiti Sultan Zainal Abidin, Besut, Malaysia; BG School of Medicine, University of Medicine and Pharmacy at Ho Chi Minh City, Ho Chi Minh, Viet Nam; BH School of Nursing, Universitas Harapan Bangsa (National Hope University), Banyumas, Indonesia; BI School of Pharmaceutical Sciences, Universiti Sains Malaysia, Pulau Penang, Malaysia; BJ School of Medicine, Caraga State University, Butuan City, Philippines; BK Elisa R. Ochoa Memorial Maternity and General Hospital, Butuan City, Philippines; BL Faculty of Dentistry, National University of Singapore, Singapore, Singapore; BM Faculty of Medicine, University of Cyberjaya, Cyberjaya, Malaysia; BN Office of Global Curtin, Curtin University, Perth, WA, Australia; BO Lee Kong Chian School of Medicine, Nanyang Technological University, Singapore, Singapore; BP Department of Medicine, National University of Singapore, Singapore, Singapore; BQ Department of Psychiatry, University of Cambridge, Cambridge, England; BR Department of Primary Care Medicine, Universiti Malaya, Kuala Lumpur, Malaysia; BS School of Pharmaceutical Science, Science University of Malaysia, Gelugor, Malaysia; BT The Interdisciplinary Research Group on Biomedicine and Health, VNU International School (VNUIS), Hanoi, Viet Nam; BU Faculty of Applied Sciences, VNU International School (VNUIS), Hanoi, Viet Nam; BV Department of Public Health and Primary Care, University of Cambridge, Cambridge, England; BW Cardiovascular Metabolic Translational Research Program, National University of Singapore, Singapore, Singapore; BX Department of Internal Medicine, Texas Tech University, Lubbock, TX, USA; BY Department of Neurosurgery, National Brain Center in Indonesia, Jakarta, Indonesia; BZ Memorial Sloan Kettering Cancer Center, Memorial Sloan Kettering Cancer Center, New York, NY, USA; CA Department of Computer Engineering, Gachon University, Seongnam-si, South Korea; CB Center of Excellence in Precision Medicine and Digital Health, Chulalongkorn University, Bangkok, Thailand; CC International Institute for Training and Research (INSTAR), Vietnam National University, Hanoi, Viet Nam; CD Department of Medicine, Can Tho University of Medicine and Pharmacy, Can Tho, Viet Nam; CE School of Population Health, University of New South Wales, Sydney, NSW, Australia; CF Ateneo Center for Research and Innovation, Ateneo De Manila University, Pasig City, Philippines; CG Demography and Health, London School of Hygiene & Tropical Medicine, London, England; CH Advanced Nursing Department, Universitas Airlangga (Airlangga University), Surabaya, Indonesia; CI La Trobe University, Melbourne, VIC, Australia; CJ Occupational Safety and Health Study Program, Diponegoro University, Semarang, Indonesia; CK Drug Delivery System Excellence Center, Prince of Songkla University Hat Yai, Hat Yai, Thailand; CL Discipline of Social and Administrative Pharmacy, Science University of Malaysia, Penang, Malaysia; CM Centre for Public Health, Equity and Human Flourishing, Torrens University Australia, Adelaide, SA, Australia; CN Institute of Resource Governance and Social Change, Kupang, Indonesia; CO Faculty of Pharmacy, AIMST University, Bedong, Malaysia; CP Faculty of Pharmcy, AIMST University, Bedong, Malaysia; CQ Department of Public Health, China Medical University, Taichung, Taiwan; CR Department of Health Policy and Administration, University of the Philippines Manila, Manila, Philippines; CS Department of Anatomy, Cebu Institute of Medicine, Cebu City, Philippines; CT Sunway Biofunctional Molecules Discovery Centre, Sunway University, Sunway City, Malaysia; CU Australian Research Centre in Complementary and Integrative Medicine, University of Technology Sydney, Ultimo, NSW, Australia; CV College of Public Health, University of the Philippines Manila, Manila, Philippines; CW Department of Hepato-Pancreatico-Biliary Surgery, Thong Nhat Hospital, Ho Chi Minh City, Viet Nam; CX Medical Research Unit, Universitas Syiah Kuala, Banda Aceh, Indonesia; CY Department of Epidemiology, Biostatistics, Population Studies and Health Promotion, Faculty of Public Health, Universitas Airlangga, Surabaya, Indonesia; CZ Health Polytechnic Ministry of Health Jakarta, Jakarta, Indonesia; DA Faculty of Nursing, Chulalongkorn University, Bangkok, Thailand; DB College of Medicine, Ajman University, Ajman, United Arab Emirates; DC Department of Nursing, Prince of Songkla University, Songkhla, Thailand; DD Department of Cardiology, National University Hospital Singapore, Singapore, Singapore; DE Epidemiological Department, Public Health Institute, Ho Chi Minh, Viet Nam; DF School of Engineering and Technology, Duy Tan University, Da Nang, Viet Nam; DG International Center for Materials Sciences and Technology, Western Caspian University, Baku, Azerbaijan; DH Pediatric Nursing Department, University of Indonesia, Depok, Indonesia; DI Department of Antropology and Sociology, Universitas Negeri Semarang, Semarang, Indonesia; DJ Department of Medical and Health Professional Education, Universiti Sultan Zainal Abidin, Kuala Terengganu, Malaysia; DK Faculty of Pharmacy, Sultan Zainal Abidin University, Malaysia, Terengganu, Malaysia; DL Department of Microbiology, Mahidol University, Bangkok, Thailand; DM Department of Pathology & Immunology, Baylor College of Medicine, Houston, TX, USA; DN Faculty of Pharmacy, Universitas Ahmad Dahlan, Yogyakarta, Indonesia; DO Department of Environmental Health, Poltekkes Kemenkes Mamuju (Health Polytechnic of the Ministry of Health, Mamuju), Mamuju, Indonesia; DP Department of Neurosciences, University of the Philippines Manila, Manila, Philippines; DQ Institute for Neurosciences, St. Luke's Medical Center, Bonifacio Global City, Philippines; DR Department of Pharmacology and Life Sciences, Universiti Teknologi MARA (UiTM), Selangor, Malaysia; DS Centre for Evidence Synthesis and Implementation Research, Cephas Health Research Initiative, Inc, Ibadan, Nigeria; DT Rothschild Foundation Hospital, Rothschild Foundation Hospital, Paris, France; DU Singapore Eye Research Institute, Singapore Eye Research Institute, Singapore, Singapore; DV Riphah School of Computing & Innovation, Riphah International University, Lahore, Pakistan; DW Department of CSE–AI&ML, Geethanjali College of Engineering and Technology, Hyderabad, India; DX Faculty of Health Sciences, Universitas Muhammadiyah Pekajangan Pekalongan, Pekalongan, Indonesia; DY Graduate School of Biomedical and Health Sciences, Hiroshima University, Hiroshima, Japan; DZ Independent Consultant, Jakarta, Indonesia; EA School of Applied Science, Republic Polytechnic, Singapore, Singapore; EB School of Health Sciences, Universiti Sains Malaysia, Kubang Kerian, Malaysia; EC School of Physical Education, Henan University, Keifeng, China; ED Department of Biostatistics and Research Methodology, Universiti Sains Malaysia, Kota Bharu, Malaysia; EE Faculty of Medicine and Health Science, Universitas Kristen Satya Wacana (Satya Wacana Christian University), Salatiga, Indonesia; EF School of Nursing, Taipei Medical University, Taipei, Taiwan; EG National Research and Innovation Agency (BRIN), Bogor, Indonesia; EH Faculty of Law, Padjadjaran University, Bandung, Indonesia; EI Clinical Research Unit of National Center for Mental Health Marzoeki Mahdi Hospital, Ministry of Health of the Republic of Indonesia, Bogor, Indonesia; EJ Department of Public Health and Epidemiology, Khalifa University of Science and Technology, Abu Dhabi, United Arab Emirates; EK Faculty of Public Health, University of Indonesia, Depok, Indonesia; EL School of Digital Science, Universiti Brunei Darussalam, Bandar Seri Begawan, Brunei; EM Institute of Applied Data Analytics, Universiti Brunei Darussalam, Bandar Seri Begawan, Brunei; EN Department of Public Health and Nutrition, National Research and Innovation Agency Republic of Indonesia, Central Jakarta, Indonesia; EO Health Policy, Airlangga Centre for Health Policy, Surabaya, Indonesia; EP Department of Physiotherapy, Universitas 'Aisyiyah Yogyakarta, Yogyakarta, Indonesia; EQ Department of Surgery, National University of Singapore, Singapore, Singapore; ER School of Medicine, Pham Ngoc Thach University of Medicine, Ho Chi Minh, Viet Nam; ES University of Medicine and Pharmacy at Ho Chi Minh City, Ho Chi Minh City, Viet Nam; ET Faculty of Electronic Engineering and Technology, Universiti Malaysia Perlis, Arau, Malaysia; EU School of Pharmacy, Monash University, Subang Jaya, Malaysia; EV School of Pharmacy, Taylor's University Lakeside Campus, Subang Jaya, Malaysia; EW Faculty of Science, Universiti Brunei Darussalam, Bandar Seri Begawan, Brunei; EX Faculty of Medicine, Universiti Sultan Zainal Abidin, Kuala Terengganu, Malaysia; EY Department of Psycho-oncology, First Affiliated Hospital of Henan Medical University, Weihui City, China; EZ Faculty of Health Sciences, Universitas Brawijaya, Malang, Indonesia; FA Division or Respiratory & Critical Care Medicine, Alexandra Hospital, Singapore, Singapore; FB Department of Medicine, University of Malaya, Kuala Lumpur, Malaysia; FC Department of Medicine and Therapeutics, The Chinese University of Hong Kong, Hong Kong, China; FD International College of Digital Innovation, Chiang Mai University, Chiang mai, Thailand; FE Department of Cardiovascular Metabolism Diseases, Duke-NUS Medical School, Singapore, Singapore; FF National Institutes of Health, University of the Philippines Manila, Manila, Philippines; FG Center for Research and Innovation, Ateneo De Manila University, Pasig City, Philippines; FH Dr. Mary and Ron Neal Cancer Center, Houston Methodist Research Institute, Houston, TX, USA; FI School of Human and Health Sciences, University of Huddersfield, Huddersfield, UK; FJ Centre for Public Health and Wellbeing, University of the West of England, Bristol, England; FK Heidelberg Institute of Global Health, Heidelberg University, Heidelberg, Germany; FL Division Prevention of Cardiovascular and Metabolic Diseases, Heidelberg University, Mannheim, Germany; FM Faculty of Humanities and Health Sciences, Curtin University, Sarawak, Malaysia; FN Jeffrey Cheah School of Medicine and Health Sciences, Monash University, Subang Jaya, Malaysia; FO Department of Epidemiology, Mahidol-Oxford Tropical Medicine Research Unit, Bangkok, Thailand; FP Nuffield Department of Medicine, University of Oxford, Oxford, England; FQ Division of Gastroenterohepatology, Universitas Airlangga (Airlangga University), Surabaya, Indonesia; FR Research Center for Public Health and Nutrition, National Research and Innovation Agency (BRIN), Bogor District, Indonesia; FS School of Pharmacy, Sunway University, Sunway City, Malaysia; FT Centre for Aquatic Foods and Global Health (AFAGH), Institute of Tropical Aquaculture and Fisheries (AKUATROP), Universiti Malaysia Terengganu, Kuala Terengganu, Malaysia; FU Preventive Medicine and Public Health Research Center, Iran University of Medical Sciences, Tehran, Iran; FV Department of Medicine, National University Health System, Singapore, Singapore; FW Department of Public Health, Universitas Negeri Semarang, Semarang, Indonesia; FX Department of Epidemiology, New York University, New York, NY, USA; FY Faculty of Pharmacy, Hasanuddin University, Makassar, Indonesia; FZ Faculty of Pharmacy, Ton Duc Thang University, Hanoi, Viet Nam; GA Institute for Global Health Innovations, Duy Tan University, Da Nang, Viet Nam; GB Department of Health Economics and Policy, Hanoi University of Public Health, Hanoi, Viet Nam; GC Department of Nutrition and Food Safety, Can Tho University of Medicine and Pharmacy, Can Tho, Viet Nam; GD Institute for Advanced Study in Technology, Ton Duc Thang University, Ho Chi Minh city, Viet Nam; GE Department of Epidemiology, University of Medicine and Pharmacy at Ho Chi Minh City, Ho chi Minh, Viet Nam; GF Department of Epidemiology, Vanderbilt University, Nashville, TN, USA; GG Cardiovascular Research Department, Methodist Hospital, Merrillville, IN, USA; GH College of Health Sciences, Nam Can Tho University, Can Tho City, Viet Nam; GI The Children's Hospital Westmead Clinical School, The University of Sydney, New South Wales, NSW, Australia; GJ International University of Health and Welfare, International University of Health and Welfare, Narita, Japan; GK Stockholm Resilience Center, Stockholm University, Stockholm, Sweden; GL College of Health Science, VinUniversity, Hanoi, Viet Nam; GM Faculty of Medicine, Universitas Negeri Padang in Indonesia, Padang, Indonesia; GN Faculty of Medicine, Universiti Malaya, Kuala Lumpur, Malaysia; GO Midwifery Department, Poltekkes Kemenkes Palu, Palu, Indonesia; GP Department of Pharmacy Practice, Universitas Airlangga (Airlangga University), Surabaya, Indonesia; GQ School of Pharmacy, Taipei Medical University, Taipei, Taiwan; GR Department of Public Health, Ministry of Health, Bandar Seri Begawan, Brunei; GS Institute of Health Sciences, Universiti Brunei Darussalam, Bandar Seri Begawan, Brunei; GT Department of Global Health and Social Medicine, Harvard University, Boston, MA, USA; GU Wellspring Research, Wellspring Center Indonesia, Jakarta, Indonesia; GV Department of Pharmaceutical Sciences and Technology, Chulalongkorn University, Bangkok, Thailand; GW National Research and Innovation Agency, National Research Council, Jakarta, Indonesia; GX College of Nursing, Krida Wacana Christian University, Jakarta, Indonesia, Taipei, Indonesia; GY Research Center for Public Health and Nutrition, National Research and Innovation Agency (BRIN) in Indonesia, Jakarta, Indonesia; GZ St Luke's Medical Center Foundation, Inc, Taguig City, Philippines; HA College of Health Sciences, VinUniversity, Hanoi, Viet Nam; HB Research Advancement Consortium in Health, Hanoi, Viet Nam; HC College of Health Sciences (CHS), VinUniversity, Hanoi, Viet Nam; HD Department of Cardiology, National University Heart Center Singapore, Singapore, Singapore; HE Department of Health and Life Sciences, Universitas Airlangga (Airlangga University), Surabaya, Indonesia; HF Department of Pharmacy Practice, Faculty of Pharmacy, Universitas Airlangga, Universitas Airlangga (Airlangga University), Surabaya, Indonesia; HG Department of Biology, Universitas Airlangga (Airlangga University), Surabaya, Indonesia; HH Faculty of Nursing, Universitas Airlangga (Airlangga University), Surabaya, Indonesia; HI Research Center in Advancing Community Healthcare, Surabaya, Indonesia; HJ School of Pharmaceutical Sciences, Universiti Sains Malaysia in Malaysia, Penang, Malaysia; HK Research Center for Public Health and Nutrition, National Research and Innovation Agency (BRIN), Jakarta, Indonesia; HL Chakri Naruebodindra Medical Institute, Mahidol University, Samut Prakan, Thailand; HM Preclinical Department, Royal College of Medicine Perak, Universiti Kuala Lumpur, Ipoh, Malaysia; HN Department of Midwifery, Ministry of Health of the Republic of Indonesia, Palu, Indonesia; HO School of Nursing & Health Sciences, Hong Kong Metropolitan University, Hong Kong, China; HP Computer Science and Engineering Department, Saveetha Institute of Medical and Technical Sciences, Chennai, India; HQ College of Nursing, Insitut Ilmu Kesehatan Bhakti Wiyata Kediri, Kediri, Indonesia; HR School of Nursing, Universitas Muhammadiyah Yogyakarta, Bantul, Indonesia; HS School of Allied Health, Anglia Ruskin University, Chelmsford, England; HT Department of Psychological Medicine, National University of Singapore, Singapore, Singapore; HU Bristol Heart Institute, University of Bristol, Bristol, England; HV Department of Population Health, Hofstra University, Hempstead, NY, USA; HW National Research and Innovation Agency (BRIN), Jakarta, Indonesia; HX Faculty of Medicine, Quest International University Perak, Ipoh, Malaysia; HY Faculty of Nursing, Mahidol University, Bangkok, Thailand; HZ Department of Smart Computing and Cyber Resilience (DSCCR), Sunway University, Selangor, Malaysia; IA School of Global Health, Chulalongkorn University, Bangkok, Thailand; IB Institute of Epidemiology and Preventive Medicine, National Taiwan University, Taipei, Taiwan; IC Benang Merah Research Center (BMRC), Minahasa Utara, Indonesia; ID Department of Epidemiology and Tropical Disease, Diponegoro University, Semarang, Indonesia; IE National Dental Research Institute Singapore, National Dental Centre Singapore, Singapore, Singapore; IF Health Services and Systems Research Programme, Duke-NUS Medical School, Singapore, Singapore; IG Department of Palliative Nursing, Widya Mandala Surabaya Catholic University in Indonesia, Surabaya, Indonesia; IH School of Computer Sciences, University of Science Malaysia, Penang Island, Malaysia; II Department of Applied Social Sciences, Hong Kong Polytechnic University, Hong Kong, China; IJ School of Chemical Sciences, University Sains Malaysia, Penang, Malaysia; IK Clinical Trials Unit, University of Warwick, Coventry, England; IL Department of Sports Medicine, University of Malaya, Kuala Lumpur, Malaysia; IM Faculty of Dentistry, University of Puthisastra, Phnom Penh, Cambodia; IN School of Health Sciences, Universiti Sains Malaysia, Kota Bharu, Malaysia; IO National Institute of Education, Nanyang Technological University, Singapore, Singapore; IP College of Public Health Sciences, Chulalongkorn University, Bangkok, Thailand; IQ Department of Internal Medicine, University of Indonesia, Jakarta, Indonesia; IR Department of Internal Medicine, Dr. Cipto Mangunkusumo National Hospital, Jakarta Pusat, Indonesia; IS Department of Medical Sciences, Sunway University, Subang Jaya, Malaysia; IT Faculty of Medical and Life Science, Sunway University, Kuala Lumpur, Malaysia; IU Faculty of Health Science, Universitas Indonesia Maju, Jakarta, Indonesia; IV Infectious and Tropical Diseases Epidemiology, Public Health Program, Monash University, Tengerang, Indonesia; IW Oxford University Clinical Research Unit Indonesia, Oxford University, Jakarta, Indonesia; IX University of Dian Nuswantoro, Semarang, Indonesia; IY Faculty of Nursing, Universitas Islam Sultan Agung, Semarang, Indonesia; IZ Department of Clinical Pathology, Brawijaya University, Malang, Indonesia; JA Hospital Central Laboratory, Dr Saiful Anwar General Hospital, Malang, Indonesia; JB Department of Microbiology, Universitas Sebelas Maret, Surakarta, Indonesia; JC Faculty of Public Health, Universitas Airlangga Indonesia, Surabaya, Indonesia; JD Joslin Diabetes Center, Harvard University, Boston, MA, USA; JE School of Pharmaceutical Sciences, University of Science Malaysia, Penang, Malaysia; JF School of Pharmacy, KPJ Healthcare University, Negeri Seremban, Malaysia; JG Department of Psychiatry, Universiti Putra Malaysia, Serdang, Malaysia; JH American Heart Association Comprehensive Hypertension Center, University of California Irvine, Orange, CA, USA; JI Excellence Center for Organ Transplantation, Mahidol University, Bangkok, Thailand; JJ College of Health Sciences, VinUniversity (VinUni), Hanoi, Vietnam, Hanoi, Viet Nam; JK Center for Global Health, University of Pennsylvania, Philadelphia, PA, USA; JL School of Biomedical Sciences, The University of Western Australia, Crawley, WA, Australia; JM University of Cyberjaya, Selangor, Malaysia; JN Faculty of Public Health, Universitas Sam Ratulangi (Sam Ratulangi University), Manado, Indonesia; JO College of Nursing, Kaohsiung Medical University, Taiwan, Kaohsiung, Taiwan; JP School of Biomedical Engineering, International University, VNU-HCM, Ho Chi Minh city, Viet Nam; JQ School of Biomedical Engineering, Viet Nam National University, Ho Chi Minh City, Viet Nam; JR International Center, Tokushima University, Tokushima, Japan; JS Second Department of Internal Medicine, Kansai Medical University, Hirakata, Japan; JT Department of Internal Medicine, University of Medicine and Pharmacy at Ho Chi Minh City, Ho Chi Minh City, Viet Nam; JU Department of Business Analytics, University of Massachusetts Dartmouth, Dartmouth, MA, USA; JV Department of Internal Medicine, Minh Anh Hospital, Ho Chi Minh, Viet Nam; JW Research and Advocacy Initiative, ALS Vietnam, Quang Ngai, Viet Nam; JX College of Health Science, Vin University, Hanoi, Viet Nam; JY Department of Occupational Health and Safety, University of Development, Surabaya, Indonesia; JZ Department of Family Medicine, National University of Singapore, Singapore, Singapore; KA Department of Family Medicine & Population Health, University of Antwerp, Antwerp, Belgium; KB Raffles Neuroscience Centre, Raffles Hospital, Singapore, Singapore; KC College of Medicine, Taipei Medical University, Taipei, Taiwan; KD Radcliffe Department of Medicine, University of Oxford, Oxford, England; KE Department of Epidemiology, Hanoi University of Public Health, Hanoi, Viet Nam; KF Faculty of Public Health, VNU University of Medicine and Pharmacy, Hanoi, Viet Nam; KG International Institute for Training and Research (INSTAR), VNU University of Medicine and Pharmacy, Hanoi, Viet Nam; KH Department of Health and Human Performance, York College/City University of New York, New York, NY, USA; KI School of Business, University of Science and Technology, 6th of October City, Egypt; KJ Research Organization for Health, National Research and Innovation Agency (BRIN), Bogor, Indonesia; KK Health Unit Organization, National Research and Innovation Agency (BRIN), Bogor, Indonesia; KL Department of Family and Preventive Medicine, Prince of Songkla University, Songkhla, Thailand; KM Department of Physical Therapy, Naresuan University, Phitsanulok, Thailand; KN Department of Cardiology, Angiology, Haemostaseology and Medical Intensive Care, Heidelberg University, Mannheim, Germany; KO Department of Experimental Pharmacology, Heidelberg University, Mannheim, Germany; KP Graduate Institute of Injury Prevention and Control, College of Public Health, Taipei Medical University, Taipei, Taiwan; KQ Department of Nursing, Universitas Aisyiyah Bandung, Bandung, Indonesia; KR Faculty of Public Health, Diponegoro University, Kota Semarang, Indonesia; KS Faculty of Public Health, Mahidol University, Bangkok, Thailand; KT Universiti Sains Malaysia, Universiti Sains Malaysia, Pulau Pinang, Malaysia; KU ASEAN Institute for Health Development, Mahidol University, Nakhon Pathom, Thailand; KV KHANA Center for Population Health Research, Phnom Penh, Cambodia; KW Department of Health Policy and Management, Jackson State University, Jackson, MS, USA; KX Asia Pacific University of Technology & Innovation, Kuala Lumpur, Malaysia; KY Department of Engineering, Universitas Airlangga (Airlangga University), Surabaya, Indonesia; KZ Department of Environmental and Occupational Health, Universiti Putra Malaysia, Serdang, Malaysia; LA Department of Public Health, Universitas Brawijaya, Malang, Indonesia; LB Global Center for Asian Women's Health (GloW), Yong Loo Lin School of Medicine, National University of Singapore, Singapore, Singapore; LC Department of Obstetrics & Gynaecology, Yong Loo Lin School of Medicine, National University of Singapore, Singapore, Singapore

## Abstract

**Background:**

The drivers of mortality and their associated risk factors evolve throughout the course of life. Understanding these transitions is essential for effective, equitable, and age-appropriate health interventions.

**Methods:**

As part of the Global Burden of Diseases, Injuries, and Risk Factors Study (GBD) 2023, we examined life expectancy and the probability of death from birth to age 70 years (70q0) from 1990 to 2023 for each Association of Southeast Asian Nations (ASEAN) member state. In addition, we assessed causes of death and risk factor by age and sex across different life stages. Life expectancy was estimated from age-specific mortality rates with standard demographic techniques, and 70q0 from mortality and GBD life tables. Cause-specific mortality was estimated using the Cause of Death Ensemble model, and risk-attributable mortality using the comparative risk assessment framework.

**Findings:**

In 2023, regional life expectancy was 70·1 years (95% uncertainty interval 69·6–70·7) for males and 76·2 years (75·7–76·7) for females, ranging from 87·5 years (87·3–87·8) for females in Singapore to 62·1 years (60·8–63·5) for males in Myanmar. Correspondingly, all-cause 70q0 was higher for males than for females. Neonatal disorders were the leading cause of mortality among infants younger than 1 year. Road injuries were the primary driver of mortality among males from adolescence through to early adulthood, accounting for 25·0% (17·0–31·0) of total mortality (8230 [5740–10 800] deaths) in males aged 15–19 years, with mortality rates varying from 3·0 per 100 000 population (2·3–3·7) to 49·0 per 100 000 population (25·0–67·1) between countries. Among females, maternal disorders were the leading cause of death in ages 25–34 years. In mid-adulthood, non-communicable diseases such as ischaemic heart disease and stroke emerged as major causes. Tobacco use was the dominant risk factor across most of adulthood in males, whereas high blood pressure was the prevailing risk factor among adult females.

**Interpretation:**

Although the leading causes of death have converged across countries, the outcomes of these causes remain divergent, reflecting persistent inequalities across the region. These patterns present a strategic opportunity for multilateral action to mobilise collective resources and achieve regional health equity.

**Funding:**

Gates Foundation.

## Introduction

The Association of Southeast Asian Nations (ASEAN) region, established in 1967, constitutes 8·6% of the global population and is one of the fastest-growing economies in the world.^1^ However, the region is characterised by stark socioeconomic asymmetries. The Human Development Index ranged from 0·946 in Singapore to 0·606 in Cambodia in 2025,^2^ whereas the projected gross domestic product per capita in 2026 ranges from more than US$107 760 in Singapore to US$1520 in Myanmar.^3^ The socioeconomic diversity fundamentally defines the region's health landscape.^4^

Since 1990, ASEAN has undergone an unevenly paced epidemiological transition, with persistent infectious and maternal and child health burdens in several states alongside rising non-communicable diseases (NCDs) in all.^5^, ^6^ Disparities in health system capacity have yielded discrepancies in health outcomes.^4^, ^7^, ^8^ Although health inequities related to specific diseases have been explored within the region,^7^, ^8^, ^9^, ^10^ no research has systematically examined comparative all-cause mortality profiles and assessed age-sex-specific patterns from birth to adulthood across the entire ASEAN.

A life-course approach addresses this gap by linking early-life exposures and structural determinants to later-life health outcomes and disparities.^11^ Earlier regional applications were confined to childhood and adolescence or individual countries, without adulthood or cross-country comparison.^12^ Because mortality drivers and their risk factors evolve across the life course,^13^, ^14^ we apply this framework using the latest findings from the Global Burden of Diseases, Injuries, and Risk Factors Study (GBD) 2023 to examine causes of death across all 11 ASEAN member states. This granular, stage-specific analysis identifies age-sex-specific diseases and risk factors driving mortality to inform targeted interventions, promote longevity, and improve health equity across the region.

**Research in context**

**Evidence before this study**

We searched PubMed from database inception to April 28, 2026, for English-language articles using the following search terms: “((“mortality” [MeSH]) AND (“cause of death” [MeSH]) AND (“risk factors” [MeSH] OR “attributable risk” OR “burden”) AND (“ASEAN” OR “Brunei” OR “Cambodia” OR “Indonesia” OR “Laos” OR “Malaysia” OR “Myanmar” OR “Philippines” OR “Singapore” OR “Timor-Leste” OR “Thailand” OR “Viet Nam”))”, which yielded 320 results. Existing disease burden studies have examined the pattern of specific diseases, injuries, or risk factors across the Association of Southeast Asian Nations (ASEAN), including country-specific analyses in Cambodia, Indonesia, Singapore, and Viet Nam. In Malaysia, a disease burden study applied a life-course approach using Global Burden of Diseases, Injuries, and Risk Factors Study 2013 data to determine all-cause and cause-specific mortality. However, the study was restricted to child and adolescent life stages and did not include cross-country comparisons with ASEAN member states. To date, no study has comprehensively examined regional and national similarities and disparities in mortality causes and assessed age-sex-specific patterns from birth to adulthood across the entire ASEAN region.

**Added value of this study**

This study characterises evolving trends in life expectancy and probability of premature mortality across all 11 ASEAN member states from 1990 to 2023. The study also provides a granular assessment of mortality drivers across life stages, sexes, and geographies. By adopting a life-course framework, we illustrated how leading causes of death varied across age groups from birth to older adulthood, and identified the specific life stages at which burdens from particular causes were most prominent. The findings show that epidemiological patterns differ across life stages, highlighting opportunities for targeted interventions.

**Implications of all the available evidence**

Our findings underscore the need for life-course-sensitive health policies across ASEAN. Mortality drivers shift throughout the lifespan, transitioning from neonatal disorders in infancy to road injuries in adolescence and young adulthood, and then to non-communicable diseases in mid to older adulthood, with each stage suggesting different policy priorities. Furthermore, sustained progress in reducing premature mortality will require addressing localised risk factors, including malnutrition and household air pollution, and tackling pervasive regional threats such as tobacco use. The ASEAN region should continue to pursue common ground and cooperative synergies to bolster health-system capacities across member states to narrow persistent health inequities.

## Methods

### Overview

Leveraging the best available data sources and a systematic analytical modelling approach, GBD 2023 produced geographically and temporally comparable estimates for various demographic and epidemiological metrics, including life expectancy and mortality for 292 causes of death, by age and sex, across 204 countries and territories, and from 1990 to 2023.^15^, ^16^

In this study, we examined life expectancy and probability of death from birth to age 70 years (70q0) across ASEAN and its member states from 1990 to 2023, and evaluated mortality patterns specific to cause and risk factor by life stages, sex, and location in 2023. Although Timor-Leste acceded to ASEAN only in October, 2025, we included it to provide the most current evidence base for the bloc as presently constituted, supporting forward-looking regional health planning. We defined life stages as: infancy and childhood (birth to age 9 years);^17^ adolescence (ages 10–24 years), following the expanded definition proposed by Sawyer and colleagues,^18^ which more closely reflects the biological, neurocognitive, and social transitions of this life stage; and adulthood (age 25 years and older).^19^

### GBD cause and risk factor hierarchy

GBD classifies causes and risk factors into four hierarchical levels. Level 1 comprises three categories: communicable, maternal, neonatal, and nutritional diseases; NCDs; and injuries, partitioned into 22 Level 2 clusters and disaggregated further at Levels 3 and 4. Risk factors are similarly organised, from three Level 1 groups (environmental and occupational, behavioural, and metabolic) into 20 Level 2 clusters with further subdivisions.^15^, ^20^ For the present analysis, we focused on causes of death at Level 3 and risk factors at Level 2.

### Data sources and processing

Data sources are detailed in the appendix (pp 6–13); mortality data were gathered from vital registrations, surveys, literature, and census data across ASEAN. Standard GBD processing corrections were applied, including age and sex splitting of deaths with insufficient information and redistribution of non-specific or implausible causes (garbage codes) to valid underlying causes. Sources were excluded if more than 50% of deaths were garbage codes or if completeness fell below 50%. Further details can be found elsewhere.^16^ Owing to gaps in primary data, estimates for certain causes and countries rely on statistical modelling (described in the Estimation methods section). The modelling process enables the production of internally consistent estimates, facilitating comparison across causes, age, sex, location, and time.

Risk factor exposure data were gathered from systematic reviews, household and health surveys, administrative records, and ground-sensing or remote-sensing data. Relative risks (RRs) were derived primarily from systematic reviews of studies reporting health outcomes associated with risk factor exposure.^15^, ^20^

### Estimation methods

#### Life expectancy

Life expectancy is the mean number of years for which an individual is expected to live at birth, assuming that age-specific mortality rates observed in a given year remain constant over the life course. Age-specific all-cause mortality rates were estimated using the OneMod framework, with country-specific all-cause mortality as the outcome and the Socio-demographic Index, HIV and COVID-19 mortality, and a summary measure of risk factor impact as covariates. These resulting rates were used to construct location-sex-specific life tables from which life expectancy was derived; further details are provided in the GBD 2023 Demography capstone.^21^

#### Probability of death

The probability of death metric reflects the likelihood of premature death within a specific age interval. Using age-specific all-cause mortality estimates and the life tables derived, we computed all-cause 70q0 by sex for each ASEAN member state from 1990 to 2023, which serves as a complementary measure of premature mortality risk.^16^

#### Cause-specific mortality

Cause-specific mortality was estimated using the Cause of Death Ensemble model.^22^ Estimates were rescaled to the all-cause mortality envelope using the Cause of Death Correction procedure so that cause-specific estimates sum to all-cause totals; full details are provided in the GBD 2023 Cause of Death capstone.^16^

#### Risk factor estimation

Risk-attributable burden was estimated using the comparative risk assessment framework, integrating population exposure, RR, the theoretical minimum risk exposure level, and underlying mortality burden. Exposures were modelled using techniques including spatiotemporal Gaussian process regression and DisMod-MR.^15^ Most risk–outcome relationships were evaluated using the Burden of Proof framework.^20^ Population-attributable fractions were calculated for each risk–outcome pair and multiplied by the corresponding cause-specific mortality to yield risk-attributable mortality by age, sex, location, and year.

#### Presentation of estimates

Cause-specific mortality estimates are presented in counts and age-specific rates per 100 000 population. For changes over time, we present percentage changes over the 1990–2023 period, calculated as the change from 1990 to 2023 relative to the 1990 baseline. Reported estimates are the mean across a 250-draw distribution, with 95% uncertainty intervals (UIs) defined as the 2·5th and 97·5th percentiles across the draws.

### Research and reporting practices

GBD 2023 complies with the Guidelines for Accurate and Transparent Health Estimates Reporting recommendations (appendix pp 4–5).^23^ The statistical code used for GBD 2023 estimation is available upon request.

### Role of the funding source

The funders of this study had no role in study design, data collection, data analysis, data interpretation, or writing of the report.

## Results

### Life expectancy and probability of death

In 2023, life expectancy in the ASEAN region was estimated at 70·1 years (95% UI 69·6–70·7) for males and 76·2 years (75·7–76·7) for females, a regional increase since 1990 of 12·2% for males and 10·3% for females. Singapore had the highest estimated life expectancy for both sexes (83·1 years [82·9–83·4] for males and 87·5 years [87·3–87·8] for females), whereas the lowest life expectancy was in Myanmar for males (61·6 years [60·1–63·0]) and Laos for females (67·2 years [66·0–68·4]). Since 1990, Laos saw the largest relative increase in life expectancy (33·3% for males and 27·5% for females), and the Philippines saw the smallest relative increase (5·0% for males and 4·3% for females; figure 1, appendix p 18).

**Figure 1:**
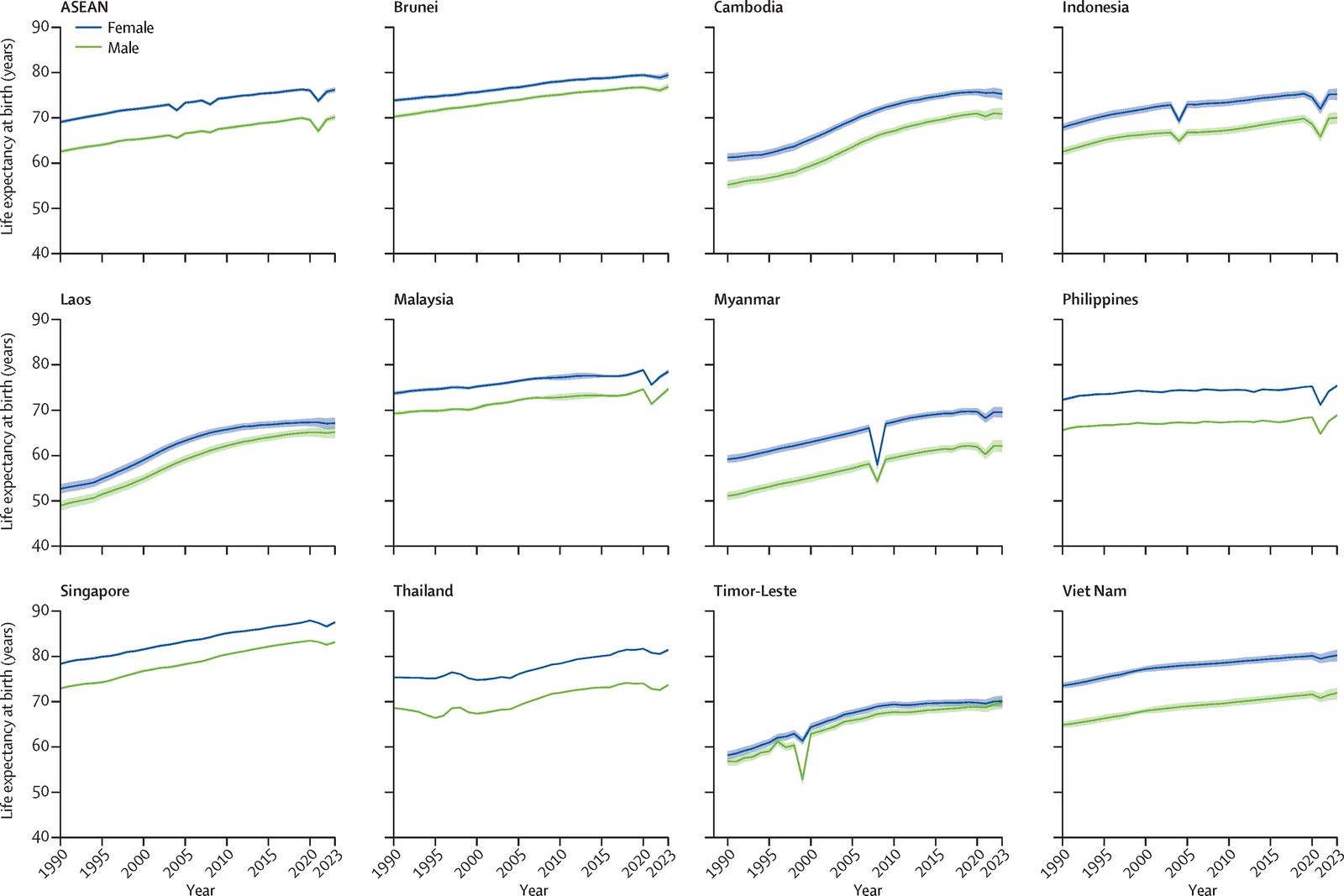
Trends in life expectancy at birth in ASEAN and its member states, 1990–2023 Lines show mean estimates of life expectancy at birth for the ASEAN region as a whole and each of its 11 member states from 1990 to 2023. Shaded areas represent 95% uncertainty intervals. Sudden declines in life expectancy at birth coincided with major mortality shocks, including the 1999 post-referendum violence in Timor-Leste, the 2004 Indian Ocean tsunami in Indonesia, the 2008 Cyclone Nargis in Myanmar, and the COVID-19 pandemic in 2021. ASEAN=Association of Southeast Asian Nations.

Across all ASEAN member states, the 70q0 from all causes combined was higher for males than for females (appendix p 19). In 2023, the regional 70q0 was estimated at 40·3% for males and 26·9% for females, and was lowest in Singapore (13·4% for males and 7·5% for females). Nine of 11 countries had male 70q0 exceeding 30%, with four surpassing 40% and the peak being in Myanmar (56·9%). Among females, four countries exceeded 30%, with the highest 70q0 observed in Laos (42·6%). Between 1990 and 2023, 70q0 decreased by 22·6% for males and 25·7% for females, most steeply in Singapore (–61·7% in males and –65·1% in females) and least in the Philippines (–7·6% in males and –8·4% in females; appendix p 19).

### Causes of death by life stages

Neonatal disorders were the leading cause of death among infants younger than 1 year, accounting for 42·9% (95% UI 36·7–49·1) of infant mortality in males (51 200 [42 700–60 300]) and 39·8% (34·1–45·8) in females (34 300 [28 900–42 200]; figure 2; appendix pp 14, 20–25). Congenital birth defects followed as the second leading cause and lower respiratory infections as the third. Among children aged 2–4 years and 5–9 years, divergence in the primary cause of death was observed between sexes (figure 2, appendix pp 20–25).

**Figure 2:**
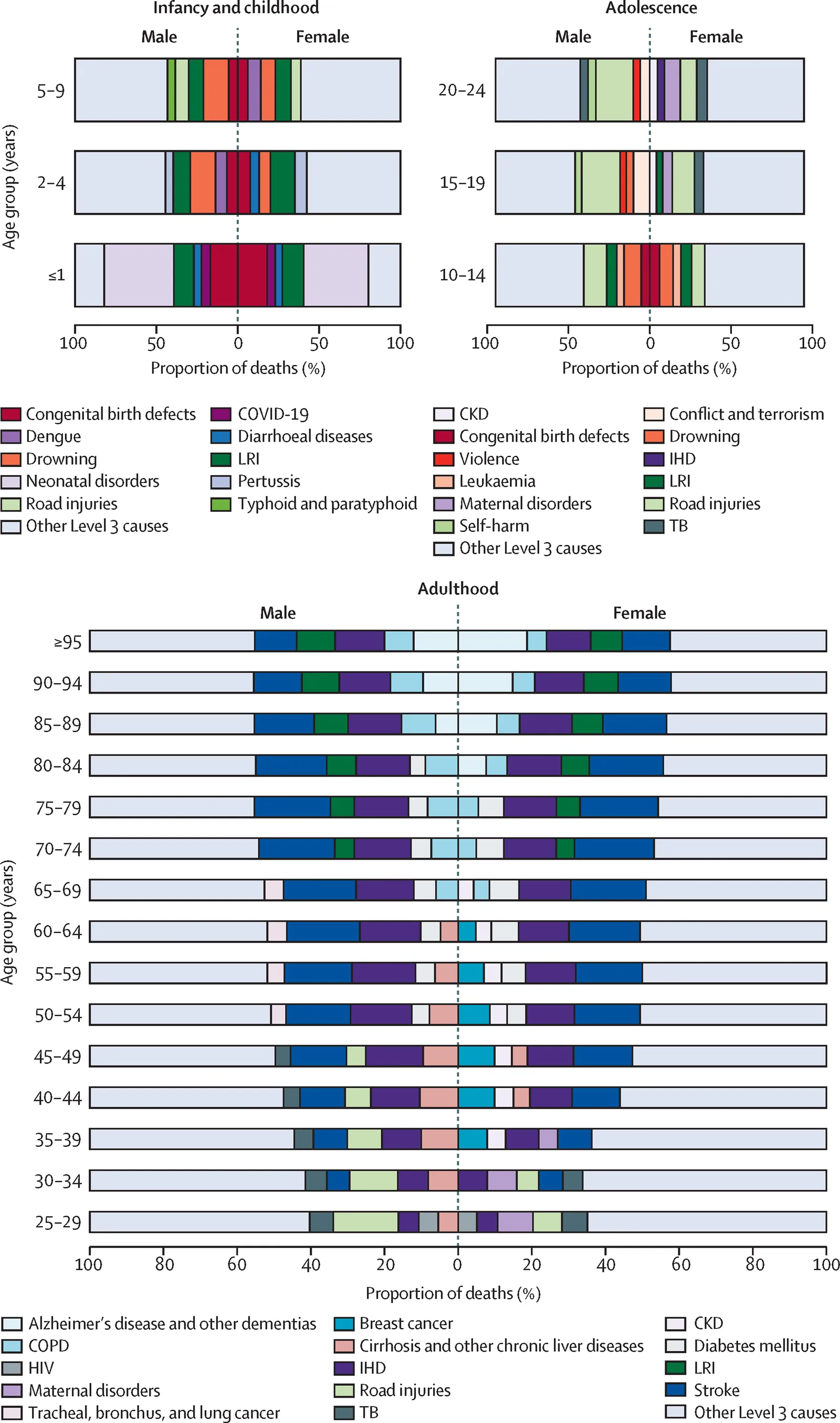
Relative contributions of top five Level 3 causes of death to total mortality by sex and age group in ASEAN, 2023 Stacked bars show the proportion (%) of total deaths attributable to the five leading Level 3 causes in each age-sex group. Other Level 3 causes comprise all remaining Level 3 causes of death beyond the top five and represent the remaining proportion of deaths in each age-sex group. ASEAN=Association of Southeast Asian Nations. CKD=chronic kidney disease. COPD=chronic obstructive pulmonary disease. HIV=HIV/AIDS. IHD=ischaemic heart disease. LRI=lower respiratory infection. TB=tuberculosis. Violence=interpersonal violence.

Underlying mortality rates differed substantially across ASEAN (appendix pp 26–61). For instance, neonatal disorder mortality among male infants younger than 1 year ranged from 55·3 deaths per 100 000 population (95% UI 40·6–64·5) in Singapore to 2261·2 deaths per 100 000 population (1740·6–2892·4) in Laos (appendix pp 38–40, 50–52). Lower respiratory infection mortality among female children aged 2–4 years ranged from 0·7 per 100 000 population (0·3–1·0) in Singapore to 56·2 per 100 000 population (32·3–91·8) in Timor-Leste (appendix pp 50–52, 56–58).

Among males aged 10–14 years, road injuries were the leading cause of death, accounting for 14·9% (95% UI 8·5–19·9) of mortality burden and a total of 2260 (1270–3040) deaths, followed by drowning. Among females in this age group, drowning predominated, resulting in 970 (672–1280) deaths, narrowly exceeding road injuries (960 [518–1350] deaths; figure 2, appendix pp 20–25). At ages 15–19 years, injury-related deaths became more prominent for males; road injuries accounted for 25·0% (17·0–31·0) of mortality burden, corresponding to 8230 (5740–10 800) deaths, followed by conflict and terrorism, drowning, and self-harm. Among females in this age group, road injuries also emerged as the top cause of death, accounting for 14·4% (8·2–19·9) of total mortality burden (3040 [1630–4270] deaths), followed by maternal disorders and tuberculosis. These patterns largely persisted into the age group of 20–24 years (figure 2, appendix pp 20–25).

Some divergence was observed in the leading causes of death among adolescents across ASEAN. For example, drowning was the leading cause of death among females aged 10–14 years in most of the region but ranked 19th in Singapore, where self-harm was the leading cause instead. Diabetes was the second (females) and fourth (males) leading cause of death for those aged 20–24 years in Brunei (appendix pp 29–31), yet diabetes did not rank within the top five in any other country (appendix pp 29–61). Cause-specific mortality rates also differed by country. Road injury mortality among males aged 15–19 years ranged from 49·0 per 100 000 population (25·0–67·1) in Thailand to 3·0 per 100 000 population (2·3–3·7) in Singapore (appendix pp 15, 50–55). For maternal disorders, a leading cause of death among females aged 20–24 years, mortality rates ranged from 43·0 per 100 000 population (22·7–65·3) in Timor-Leste to 0·1 per 100 000 population (0·1–0·1) in Singapore (appendix pp 16, 50–52, 56–58).

Road injuries remained the prevailing cause of death among young adult males aged 25–34 years (figure 2, appendix pp 20–25). Ischaemic heart disease rose to become the leading cause of death by ages 35–39 years before being replaced by stroke from age 50 years onward. Male cirrhosis and other chronic liver disease mortality ranked fourth at ages 25–29 years and peaked at ages 40–44 years (10·4% [8·4–12·9], 11 800 [8860–15 300] deaths; figure 2, appendix pp 20–25), remaining a top three cause during ages 30–59 years. Stroke was the leading cause of death through most of mid to late adulthood, peaking at ages 70–74 years (59 100 [47 900–73 000] deaths; figure 2, appendix pp 20–25).

Among females, maternal disorders were the leading cause of death at ages 25–34 years, accounting for nearly 10% of total mortality. From age 35 years, stroke became the leading cause, followed by ischaemic heart disease; this ranking was maintained up to ages 85–89 years. Diabetes rose to third by ages 65–69 years (figure 2, appendix pp 20–25). By ages 60–64 years, the top three causes of death were stroke, ischaemic heart disease, and diabetes. Towards late adulthood the sexes converged: stroke, ischaemic heart disease, diabetes, chronic obstructive pulmonary disease, and lower respiratory infections were the top five at ages 70–79 years. Alzheimer's disease rose to become a major cause in the oldest age groups (figure 2, appendix pp 20–25).

Leading causes of mortality diverged across countries in early adulthood, particularly among females. At ages 25–29 years, road injuries dominated in Malaysia, Thailand, and Viet Nam; maternal disorders in Indonesia, Myanmar, the Philippines, and Timor-Leste; lower respiratory infections in Laos; HIV/AIDS in Cambodia; diabetes in Brunei; and self-harm in Singapore (appendix pp 29–61). Causes converged by mid to late adulthood but outcomes stayed disparate. Stroke showed the greatest disparity: at ages 55–59 years, female stroke mortality ranged from 7·1 deaths per 100 000 population (95% UI 4·4–9·5) in Singapore to 248·3 per 100 000 population (158·0–369·0) in Myanmar, with a similar gradient among males (appendix pp 17, 44–46, 50–52).

### Mortality burden attributable to risk factors

In 2023, malnutrition was the leading risk factor in infancy and childhood in ASEAN, accounting for 48·4% (95% UI 42·1–55·4) of the mortality burden in male infants younger than 1 year (57 800 [48 900–66 600] deaths) and 45·2% (39·4–50·8) in females (38 800 [33 400–45 000] deaths; figure 3, appendix pp 62–67). Air pollution was the second leading risk factor in infancy, whereas unsafe water, sanitation, and hygiene grew in relative importance throughout childhood. During adolescence, occupational risks emerged as a leading factor among males aged 15–19 years, accounting for 4·8% (3·3–6·5) of mortality burden and 1570 (1080–2230) deaths, followed by high alcohol use; for females aged 15–19 years, kidney dysfunction and occupational risks were the two leading factors (figure 3, appendix pp 62–67). Across adulthood, tobacco was the leading risk factor among males (13–27% of mortality burden at ages 30–64 years). From age 65 years onwards, high blood pressure replaced tobacco as the leading risk, contributing to more than one-quarter of mortality burden during late adulthood. Dietary risks and air pollution also contributed consistently (figure 3, appendix pp 62–67). For females, unsafe sex and dietary risks were the top two risk factors for mortality among young adults aged 25–34 years. From age 35 years onwards, dietary risks and high blood pressure emerged as the leading risk factors and remained among the top five throughout adulthood (figure 3, appendix pp 62–67). High BMI was another key risk factor, particularly in mid-adulthood, contributing to 11·0% (5·6–16·3) of mortality among females aged 50–54 years (13 300 [6560–20 000] deaths). Air pollution contributed to more than 15% of mortality among both males and females aged 70 years and older (figure 3, appendix pp 62–67).

**Figure 3:**
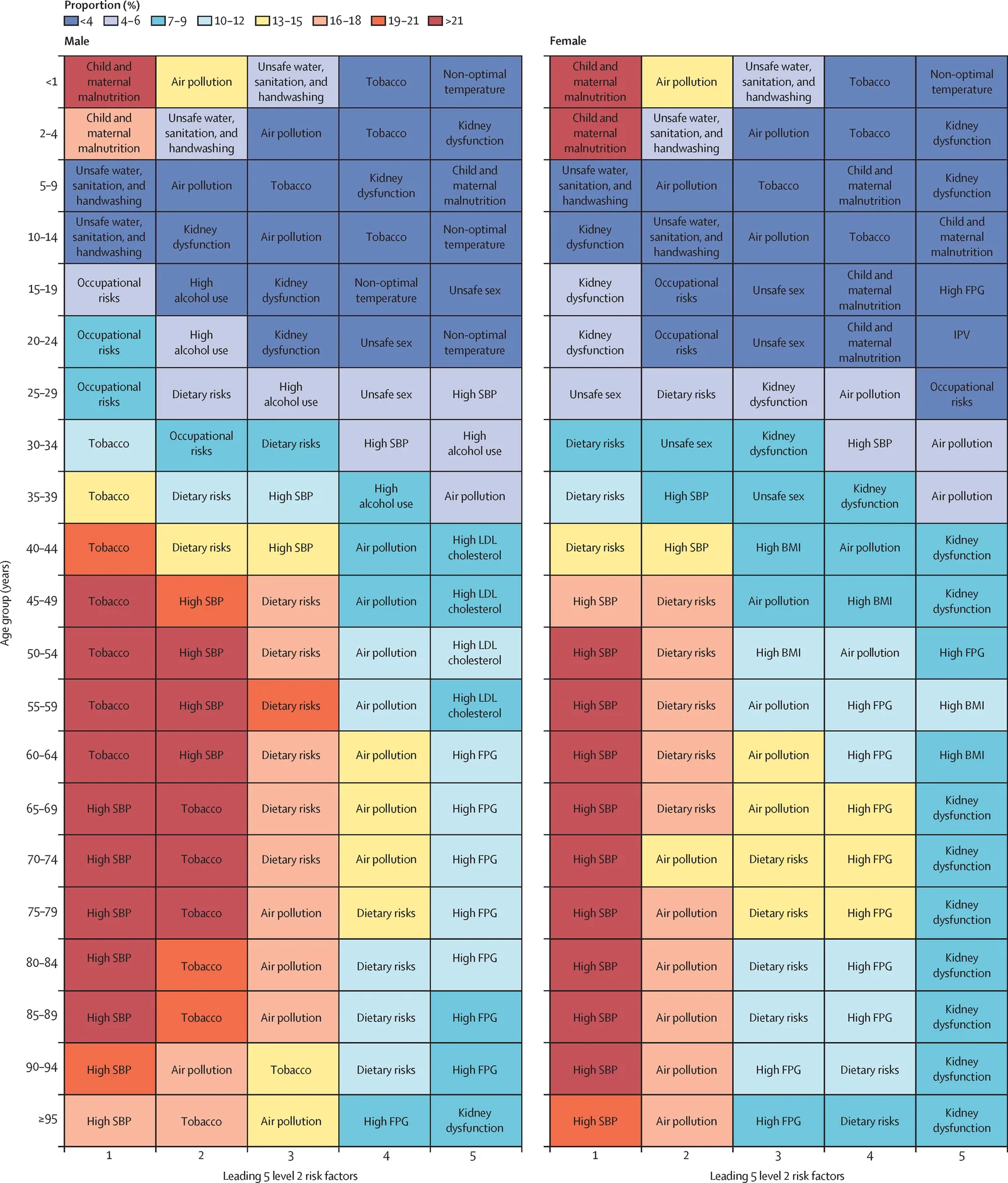
Relative contributions of top five Level 2 risk factors to total mortality by sex and age groups in ASEAN, 2023 Heatmaps show the proportion (%) of total deaths attributable to the five leading Level 2 risk factors in each age-sex group. ASEAN=Association of Southeast Asian Nations. FPG=fasting plasma glucose. IPV=intimate partner violence. SBP=systolic blood pressure.

## Discussion

This study provides a life-course perspective on the drivers of premature mortality across the ASEAN region. Our findings reveal substantial disparities in life expectancy and 70q0, with a life expectancy gap as wide as 20 years between the lowest-performing and highest-performing member states. Throughout the life course, leading mortality drivers showed similar age patterns: neonatal disorders dominate infancy; lower respiratory infections prevail in early childhood and drowning in later childhood to early adolescence; road injuries impose a substantial burden in adolescence and young adulthood, particularly among males; maternal disorders account for a notable share among young females in many ASEAN states; and NCDs, including ischaemic heart disease, stroke, and diabetes, dominate from mid-adulthood onwards. Yet, outcomes diverge markedly; neonatal mortality rates, for instance, differ 40-fold between the highest-performing and lowest-performing countries. These patterns underscore substantial health inequities and differences in health-system capacity, suggesting opportunities for joint regional action.

The increase in life expectancy and reduction in 70q0 between 1990 and 2023 reflect remarkable achievements in population health. The COVID-19 pandemic was associated with a temporary decline in 2020–21, before life expectancy resumed its upward trajectory. These advancements were accompanied by socioeconomic development, including gross domestic product growth, rising Human Development Index, and expanded universal health coverage.^24^, ^25^ However, progress remains uneven. In 2024, Singapore's gross domestic product per capita was 65 times that of Myanmar, with government health spending at 21% of public expenditure versus 4% in Myanmar.^26^ The divergence is mirrored in health outcomes across the region.

The leading causes of death echo the 15 priority conditions in the Global Health 2050 report by the *Lancet* Commission on Investing in Health, which targets a 50% reduction in probability of premature death before age 70 years by 2050.^27^ These conditions—spanning eight neonatal, childhood, maternal, and infectious causes and seven NCDs—align closely with the causes of death identified here. By applying a life-course perspective, our analysis highlights how the leading causes of death vary across life stages and identifies when burdens are most salient.

In the early years of life, neonatal disorders constitute the leading cause of death among infants younger than 1 year across ASEAN. This category includes, in order of magnitude: neonatal preterm birth, neonatal encephalopathy, neonatal sepsis, neonatal haemolytic disease, and other neonatal disorders, with the first two accounting for more than 30% of neonatal deaths. Although the predominance of neonatal disorders among infants younger than 1 year is expected, the magnitude of the disparity in mortality rates is concerning. High mortality from neonatal disorders is likely to reflect inadequacies in obstetric services.^28^ Several conditions are strongly associated with modifiable maternal risk factors, including hypertensive disorders of pregnancy, haemorrhagic conditions, anaemia, and maternal obesity.^29^ Managing these risks requires robust antenatal care, yet access remains restricted in resource-constrained countries; in Laos, only 68·9% of pregnant women attended four or more antenatal care visits in 2023,^30^ falling far short of the 90% WHO, UNICEF, and UN Population Fund target.^31^ Restricted access to facility-based delivery further compounds neonatal risks. In Timor-Leste and Myanmar, the proportions of facility births are among the lowest in the region, at 58·1% and 67·6% respectively.^30^ Beyond delivery services, many facilities have insufficient neonatal intensive care and diagnostic capacity.^32^ Furthermore, national newborn screening programmes remain unevenly implemented. Whereas Singapore, Thailand, and the Philippines have long-established screening programmes spanning birth defects, hearing and vision impairment, and metabolic and endocrine disorders, Cambodia, Laos, and Myanmar face persistent coverage gaps, probably widening inequities in early detection and intervention.^33^, ^34^

During late childhood, adolescence, and young adulthood, various types of injuries emerge as key drivers of premature mortality. Drowning was the leading cause of mortality among male children aged 2–9 years, and remained prominent into early adolescence. This burden is preventable through layered strategies, from community measures such as water-access barriers and swimming skills training to water-transport and disaster-preparedness policies, yet these remain unevenly implemented across ASEAN.^8^, ^35^ Feasible approaches include adaptation of effective regional programmes, such as Viet Nam's survival swimming curricula and Thailand's Child Drowning Prevention committee, alongside other WHO-recommended interventions.^8^

During adolescence and young adulthood, road injuries became the leading cause of death among males aged 15–34 years, with substantial regional differences. Potential contributors include high motorcycle use, low helmet and seatbelt use compliance, gaps in traffic law enforcement, and insufficient post-crash emergency care in parts of the region.^8^ Although ASEAN introduced a regional road-safety strategy in 2016 addressing five pillars of road safety, reducing road-injury mortality remains a public health priority for the region.^8^, ^36^ Deviating from regional patterns, self-harm was the leading driver of mortality among adolescents and young adults in Singapore. Increasing self-harm rates have also been documented in Malaysia and Brunei.^37^ These patterns reflect multiple interacting determinants, including underlying mental health conditions, psychological and interpersonal stressors, and structural barriers to care.^9^, ^38^ Although prevention approaches vary across ASEAN, two priorities recur: destigmatising and decriminalising suicide attempts, which are still punishable in Brunei and Myanmar; and strengthening self-harm surveillance, which remains sparse across the region.^8^, ^39^

The prevailing injury types at each life stage are broadly consistent with each country's socioeconomic development, following the pattern of the epidemiological transition.^15^ Some rates nonetheless stand out against global GBD 2023 results: drowning among children aged 5–9 years in Cambodia, Timor-Leste, and Viet Nam ranked within the 20 highest globally,^15^ and road injuries among males aged 15–19 years in Thailand ranked second among upper-middle-income countries. Reducing these preventable deaths calls for a concerted multisectoral response.^8^, ^40^

Throughout mid to late adulthood, ischaemic heart disease, stroke, diabetes, chronic obstructive pulmonary disease, and cirrhosis and other chronic liver diseases were the leading causes of death across ASEAN, reflecting the region's epidemiological transition. Although disease rankings were similar, outcomes diverged substantially, probably reflecting unequal access to care and differences in modifiable behavioural risk exposure. Among these risks, tobacco was the leading risk factor among those aged 30–64 years, with more than one-quarter of deaths in males aged 55–69 years attributable to this risk. Yet, tobacco control remains unevenly implemented under the WHO Framework Convention on Tobacco Control (FCTC). Singapore has one of the region's lowest male smoking prevalences following comprehensive measures, whereas Indonesia, where male smoking prevalence exceeds 50%,^10^ remains the only member state not a party to the FCTC. Strengthening FCTC implementation, drawing on models such as Singapore's, could reduce tobacco-attributable mortality in ASEAN.^10^ More broadly, primary prevention offers a cost-effective approach to avert long-term NCD burden and systemic strain.^41^

Distinctive risk factors shape mortality at different life stages. In early childhood, malnutrition predominated, accounting for more than 40% of under-5 deaths. During adolescence, occupational risks predominated among males, and kidney dysfunction and occupational risks affected females, the former partly reflecting regional vulnerabilities associated with unequal access to education, early workforce entry, and inadequate social protection.^42^ In adulthood, metabolic and dietary risk factors became increasingly salient. Air pollution weighed most heavily in childhood and late adulthood, although its dominant source, whether household air pollution from solid fuels or ambient particulate matter, varied by age and geography.^43^ Addressing these sources requires accelerating clean cooking in countries with high household air pollution burden^44^ and strengthening regional governance to reduce transboundary haze,^45^ both demanding investments and coordination across national and regional levels.^46^, ^47^ Unlike most risk factors concentrated within specific life stages, air pollution acts continuously across the life course, from early childhood to late adulthood, making air-quality policy uniquely high leverage.

This study has certain limitations. First, the precision of cause-specific mortality estimates depends on the quality of available data. Many ASEAN countries do not have complete vital registration, surveillance systems, and medically verified cause-of-death data; estimates for these settings therefore rely on statistical models using covariates and spatiotemporal patterns, and carry wider 95% UIs. Cross-country comparisons should be interpreted with caution, and these gaps highlight the need to strengthen national health information systems to improve future estimates and policy relevance. Second, the region has experienced major mortality shocks between 1990 and 2023, including natural disasters, civil conflicts, and the COVID-19 pandemic. Extensive efforts have been made to correct for these events; for instance, COVID-19 deaths misclassified as other causes were identified and corrected in the vital registration data through a dedicated machine-learning procedure. Nonetheless, the variable quality and inherent stochasticity of data from these periods hamper the precision of estimates.^21^ Third, although 70q0 provides a complementary summary of premature mortality, it does not have the granularity to identify the specific ages at which mortality risks are most concentrated. Fourth, because this analysis applies a life-course perspective using age-stratified estimates rather than longitudinal cohort data, it does not capture individual-level trajectories or distinguish age, period, and cohort effects. Our life-stage comparisons should be interpreted in this light. Finally, risk factors examined are not exhaustive. GBD inclusion requires sufficient evidence of causation as defined under the Burden of Proof methodology,^20^ so risk factors with insufficient evidence were excluded, potentially omitting emerging environmental and behavioural drivers of disease burden.

In conclusion, by examining causes of death across the life course, our findings illustrate how mortality drivers shift with age, underscoring the need for interventions tailored to each life stage. Between 1990 and 2023, mortality profiles across ASEAN have become more similar in composition, although the associated burden still differs substantially. Achieving equitable progress will require robust information systems and sustained multilateral cooperation, anchored in ASEAN's health-governance architecture, including the ASEAN Community Vision 2045 and its ASEAN Socio-Cultural Community Strategic Plan (2026–35), to address shared regional determinants and elevate health as a collective priority.

### GBD 2023 ASEAN Causes of Death Collaborators

### Affiliations

### Contributors

### Data sharing

To download the input data used in these analyses, please visit the Global Health Data Exchange at http://ghdx.healthdata.org/gbd-2023. All results from this study are publicly accessible. To download estimates produced in these analyses, please visit the GBD Results tool at https://vizhub.healthdata.org/gbd-results/.

## Declaration of interests

MSA reports grants or contracts from the Xiamen University Malaysia Research Fund (XMUMRF/2025-C15/ITCM/0006, XMUMRF/2023-C11/ISEM/0041, and XMUMRF/2026-C17/ITCM/0007; all internal research grants administered by Xiamen University Malaysia and disbursed to institution). JBA reports support from the University of Eastern Pangasinan through their employment. RJM reports support from the Wellcome Trust (315982/Z/24/Z). (For the purpose of Open Access, RJM has applied a CC BY public copyright license to any Author Accepted Manuscript version arising from this submission. This grant provides core funding for Mahidol Oxford Tropical Medicine Research and contributes to RJM's salary. RJM is requested by Wellcome to include this text in the funding statement and cover letter of all manuscripts on which RJM is a coauthor.) SO reports research fellowship support from the Lynch Family Foundation; grants or contracts from NHMRC Australia (EL1 Investigator Grant); and a leadership or fiduciary role in other board, society, committee or advocacy group, paid or unpaid with International Association for Suicide Prevention (Vice President 2025–26). YLS reports grants or contracts from NTU EPM & NSTC, Taiwan (postdoctoral fellow contract); a leadership or fiduciary role in other board, society, committee or advocacy group, paid or unpaid with Benang Merah Research Center (co-founder); and other financial or non-financial interests with Jago Beasiswa (international scholarship mentor). JHVT reports a leadership or fiduciary role in other board, society, committee or advocacy group, paid or unpaid with Benang Merah Research Center (co-founder).
