# Supplementary material for: Causes of death across the life course in the Association of Southeast Asian Nations (ASEAN): a systematic analysis for the Global Burden of Disease Study 2023
Source: Lancet Public Health. 2026 Sep 22;11(10):e786–96. doi: 10.1016/S2468-2667(26)00159-3 (PMC13630716; doi:10.1016/S2468-2667(26)00159-3)
Supplement: Supplementary file 1 [file mmc1.pdf]

THE LANCET
Public Health

Supplementary appendix

This appendix formed part of the original submission and has been peer reviewed. We post it as supplied by the authors.

Supplement to: GBD 2023 ASEAN Causes of Death Collaborators. Causes of death across the life course in the Association of Southeast Asian Nations (ASEAN): a systematic analysis for the Global Burden of Disease Study 2023. *Lancet Public Health* 2026; **11:** e786–96.

Appendix

Supplement to: "Causes of death across the life course in the Association of Southeast Asian Nations (ASEAN): a systematic analysis for the Global Burden of Disease Study 2023"

Table of Contents

Statement of GATHER Compliance 4

Supplementary methods: data sources 6

Supplementary results 14

Figure S1 | Relative contribution (%) of Level 2 causes of death to the total mortality by sex and age group, ASEAN, 2023 14

Figure S2 | Mortality rates per 100 000 population (95% UI) attributable to road injuries in ASEAN and its member states for A) males and B) females, across high-burden age groups (in years), 2023. .. 15

Figure S3 | Mortality rates per 100 000 population (95% UI) attributable to maternal disorders in ASEAN and its member states, 2023. 16

Figure S4 | Mortality rates per 100 000 population (95% UI) attributable to stroke in ASEAN and its member states for A) males and B) females, among adults aged 25–59 years, 2023. 17

Table S1 | Sex-disaggregated life expectancy at birth (in years) in 1990 and 2023, and its percentage change from 1990 to 2023, in ASEAN and its member states 18

Table S2 | Sex-disaggregated all-cause probability of death from birth to age 70 years (70q0) in 1990 and 2023, and its percentage change from 1990 to 2023, in ASEAN and its member states 19

Table S3 | Relative contribution (%) of top five Level 3 causes of death to the total mortality by sex and age groups in ASEAN, 2023 20

Table S4 | Absolute number of top five Level 3 causes of death by sex and age groups in ASEAN, 2023 23

Table S5 | Mortality rates (per 100 000) for the top five Level 3 causes of death by sex and age groups in ASEAN, 2023 26

Table S6 | Mortality rates (per 100 000) for the top five Level 3 causes of death by sex and age groups in Brunei, 2023 29

Table S7 | Mortality rates (per 100 000) for the top five Level 3 causes of death by sex and age groups in Cambodia, 2023 32

Table S8 | Mortality rates (per 100 000) for the top five Level 3 causes of death by sex and age groups in Indonesia, 2023 35

Table S9 | Mortality rates (per 100 000) for the top five Level 3 causes of death by sex and age groups in Laos, 2023 38

Table S10 | Mortality rates (per 100 000) for the top five Level 3 causes of death by sex and age groups in Malaysia, 2023 41
Table S11 | Mortality rates (per 100 000) for the top five Level 3 causes of death by sex and age groups in Myanmar, 2023 44
Table S12 | Mortality rates (per 100 000) for the top five Level 3 causes of death by sex and age groups in Philippines, 2023 47
Table S13 | Mortality rates (per 100 000) for the top five Level 3 causes of death by sex and age groups in Singapore, 2023 50
Table S14 | Mortality rates (per 100 000) for the top five Level 3 causes of death by sex and age groups in Thailand, 2023 53
Table S15 | Mortality rates (per 100 000) for the top five Level 3 causes of death by sex and age groups in Timor-Leste, 2023 56
Table S16 | Mortality rates (per 100 000) for the top five Level 3 causes of death by sex and age groups in Viet Nam, 2023 59
Table S17 | Relative contribution (%) of top five Level 2 risk factors to the total mortality by sex and age groups in ASEAN, 2023 62
Table S18 | Absolute number of deaths attributable to the top five Level 2 risk factors by sex and age groups in ASEAN, 2023 65
Author list 68
Authors' affiliations 70
Authors' contributions 78
Providing data or critical feedback on data sources 78
Developing methods or computational machinery 78
Providing critical feedback on methods or results 78
Drafting the manuscript or revising it critically for important intellectual content 80
Managing the estimation or publications process 81

Statement of GATHER Compliance

Item #	Checklist item	Reporting location
**Objectives and funding**		
1	Define the indicator(s), populations (including age, sex, and geographic entities), and time period(s) for which estimates were made.	○ Main text: Introduction, p 7<br>○ Main text: Methods (Overview), p 9
2	List the funding sources for the work.	○ Summary (Funding), p 4<br>○ Acknowledgments, p 35
**Data Inputs**		
For all data inputs from multiple sources that are synthesized as part of the study:		
3	Describe how the data were identified and how the data were accessed.	○ Main text: Methods, p 9-13
4	Specify the include and exclusion criteria· Identify all ad-hoc exclusions.	○ Main text: Methods, p 9-13
5	Provide information on all included data sources and their main characteristics· For each data source used, report reference information or contact name/institution, population represented, data collection method, year(s) of data collection, sex and age range, diagnostic criteria or measurement method, and sample size, as relevant.	○ Main text: Methods (Data sources and processing), p 10
6	Identify and describe any categories of input data that have potentially important biases (e.g., based on characteristics listen in item 5).	○ Main text: Methods, p 9-13
For data inputs that contribute to the analysis but were not synthesized as part of the study:		
7	Describe and give sources for any other data inputs.	○ Not Applicable
For all data inputs:		
8	Provide all data inputs in a file format from which data can be efficiently extracted (e.g., a spreadsheet rather than a PDF), including all relevant meta-data listed in item 5· For any data inputs that cannot be shared because of ethical or legal reasons, such as third-party ownership, provide a contact name or the name of the institution that retains the right to the data.	○ Included in an online tool (Global Health Data Exchange website: http://ghdx.healthdata.org/gbd-2023
**Data analysis**		
9	Provide a conceptual overview of the data analysis method· A diagram may be helpful.	○ Main text: Methods, p 9-13

10	Provide a detailed description of all steps of the analysis, including mathematical formulae· This description should cover, as relevant, data cleaning, data pre-processing, data adjustments and weighting of data sources, and mathematical or statistical model(s).	○ Main text: Methods, p 9-13
11	Describe how candidate models were evaluated and how the final model(s) were selected.	○ Found in GBD 2023 Disease and Injury and Risk Factor Collaborators (Methods, p 9-13)<br>○ Found in GBD 2023 Demography Capstone (Methods, p 11-13)<br>○ Found in GBD 2023 Causes of Death Capstone (Methods, p 11-13)<br>○ Found in GBD 2021 Risk Factors Collaborators (Methods, p 13)
12	Provide the results of an evaluation of model performance, if done, as well as the results of any relevant sensitivity analysis.	○ Found in GBD 2023 Disease and Injury and Risk Factor Collaborators (Methods, p 9-13)<br>○ Found in GBD 2023 Demography Capstone (Methods, p 11-13)<br>○ Found in GBD 2023 Causes of Death Capstone (Methods, p 11-13)<br>○ Found in GBD 2021 Risk Factors Collaborators (Methods, p 13)
13	Describe methods for calculating uncertainty of the estimates· State which sources of uncertainty were, and were not, accounted for in the uncertainty analysis.	○ Main text: Methods (Presentation of estimates), p 13
14	State how analytic or statistical source code used to generate estimates can be accessed.	○ Main text: Methods (Research and reporting practices), p 13

Supplementary methods: data sources

The table below summarises all mortality data sources used in the Global Burden of Disease (GBD) 2023 study for ASEAN countries, 1990-2023.

ASEAN Country	Year	Citation
Brunei	1960	Joint Census Department (Borneo). Brunei Population Census 1960. as it appears in United Nations Statistics Division (UNSD). United Nations Demographic Yearbook - Historical Supplement 1997. New York, United States of America: United Nations (UN).
	1950–1959; 1964–1969; 1970–1978; 1982–1992	Brunei Vital Registration Death Data 1950-1959, 1964-1969, 1970-1978, 1982-1992. as it appears in United Nations Statistics Division (UNSD). United Nations Demographic Yearbook - Historical Supplement 1997. New York, United States of America: United Nations (UN).
	1980-1999	Vital Registration. World Health Organization (WHO). Country Mortality Data 1980-1999.
	2017	World Health Organization (WHO). WHO Mortality Database Version March 2017. Geneva, Switzerland: World Health Organization (WHO).
	1996–2019	Brunei Darussalam Vital Registration - Deaths 1996-2019 ICD10. as it appears in World Health Organization (WHO). WHO Mortality Database (multiple versions: November 2015, October 2017, December 2018, December 2019, March 2021). Geneva, Switzerland: World Health Organization (WHO).
	2020, 2022	Vital Registration. Ministry of Finance and Economy (Brunei Darussalam). Brunei Darussalam Vital Statistics 2020 and 2022. Brunei Darussalam: Ministry of Finance and Economy (Brunei Darussalam).
Cambodia	1959	Cambodia Sample Survey 1959. as it appears in United Nations Statistical Office. United Nations Demographic Yearbook 1959. New York, United States of America: United Nations (UN), 1960.
	1997; 1999; 2003–2005	National Institute of Statistics (Cambodia), World Bank, United Nations Development Programme (UNDP), and Statistics Sweden. Cambodia Socio-Economic Survey 1997, 1999, and 2003-2005. Phnom Penh, Cambodia: National Institute of Statistics (Cambodia).
	1998	Macro International, Inc, Ministry of Health (Cambodia), National Institute of Public Health (Cambodia). Cambodia Special Demographic and Health Survey 1998. Fairfax, United States of America: ICF International.
	1998; 2008	National Institute of Statistics (Cambodia), Minnesota Population Center. Cambodia General Population Census 1998 and 2008 from the Integrated Public Use Microdata Series, International: [Machine-readable database]. Minneapolis: University of Minnesota.
	2000; 2005–2006; 2010–2011; 2014; 2021–2022	Macro International, Inc/ICF Macro/ICF International, Ministry of Health (Cambodia), National Institute of Statistics (Cambodia), and National Institute of Public Health (Cambodia). Cambodia Demographic and

		Health Survey 2000, 2005-2006, 2010-2011, 2014, and 2021-2022. Fairfax, United States of America: ICF International.
	2004	National Institute of Statistics (Cambodia). Cambodia Intercensal Population Survey 2004.
	2004; 2013	Minnesota Population Center. Integrated Public Use Microdata Series, International: Version 7.2 [Cambodia Inter-Censal Population Survey 2004 and 2013]. Minneapolis, MN: IPUMS, 2019. https://doi.org/10.18128/D020.V7.2
Indonesia	1964–1965; 1985; 1992–2013	Central Bureau of Statistics (Indonesia), Ministry of Health (Indonesia), United Nations Children's Fund (UNICEF), World Bank, and Statistics Indonesia. Indonesia National Socioeconomic Survey 1964-1965, 1985, and 1992-2013. Jakarta, Indonesia: Central Bureau of Statistics (Indonesia)/Statistics Indonesia.
	1971; 1980; 1990; 2000	Central Bureau of Statistics (Indonesia) and Statistics Indonesia. Indonesia Population and Housing Census 1971, 1980, 1990, and 2000.
	1971; 1980; 1990; 2000; 2010	Minnesota Population Center, Central Bureau of Statistics (Indonesia), and Statistics Indonesia. Indonesia Population Census 1971, 1980, 1990, 2000, and 2010 from the Integrated Public Use Microdata Series, International: [Machine-readable database]. Minneapolis: University of Minnesota, 2012.
	1976	Central Bureau of Statistics (Indonesia) and International Statistical Institute. Indonesia World Fertility Survey 1976. Voorburg, Netherlands: International Statistical Institute.
	1976; 1995	Minnesota Population Center and Central Bureau of Statistics (Indonesia). Indonesia Intercensal Population Survey 1976 and 1995 from the Integrated Public Use Microdata Series, International: [Machine-readable database]. Minneapolis: University of Minnesota, 2012.
	1985; 1995; 2005	Central Bureau of Statistics (Indonesia) and Statistics Indonesia. Indonesia Intercensal Population Survey 1985, 1995, and 2005.
	1987; 1991; 1994; 1997; 2002–2003; 2007; 2012; 2017	Central Bureau of Statistics (Indonesia), National Family Planning Coordinating Board (Indonesia), Westinghouse; Institute for Resource Development, Macro International, Inc, Ministry of Health (Indonesia), Statistics Indonesia, ICF International, and National Population and Family Planning Board (Indonesia). Indonesia Demographic and Health Survey 1987, 1991, 1994, 1997, 2002-2003, 2007, 2012, and 2017. Fairfax, United States of America: ICF International.
	1993–1994; 1997; 2000; 2007–2008; 2014–2015	RAND Corporation, University of Indonesia, Macro International, Inc, University of California, Los Angeles (UCLA), Center for Population and Policy Studies, Gadjah Mada University (Indonesia), and SurveyMETER. Indonesia Family Life Survey 1993-1994, 1997, 2000, 2007-2008, and 2014-2015. Santa Monica, United States of America: RAND Corporation.
	1997	United Nations Statistics Division (UNSD). United Nations Demographic Yearbook - Historical Supplement 1997. New York, United States of America: United Nations (UN).
	2010	Statistics Indonesia. Indonesia Population Census 2010. as it appears in United Nations Statistics Division (UNSD). United Nations Demographic Yearbook 2018. New York, United States of America: United Nations (UN), 2019.

Indonesia *(continued from the previous page)*	2011	Ministry of Home Affairs (Indonesia), National Development Planning Agency (BAPPENAS) (Indonesia), Statistics Indonesia, and United Nations Children's Fund (UNICEF). Indonesia - Papua Multiple Indicator Cluster Survey 2011. New York, United States of America: United Nations Children's Fund (UNICEF), 2013.
	2011	Ministry of Home Affairs (Indonesia), National Development Planning Agency (BAPPENAS) (Indonesia), Statistics Indonesia, and United Nations Children's Fund (UNICEF). Indonesia - West Papua Multiple Indicator Cluster Survey 2011. New York, United States of America: United Nations Children's Fund (UNICEF), 2013.
	2012	National Team for the Acceleration of Poverty Reduction (TNP2K) (Indonesia), SurveyMETER, University of Southern California, and World Bank. Indonesia Family Life Survey East 2012.
	2012	ICF International, Ministry of Health (Indonesia), National Population and Family Planning Board (Indonesia), and Statistics Indonesia. Indonesia Special Demographic and Health Survey 2012. Fairfax, United States of America: ICF International.
	2016–2017	National Population and Family Planning Board of Indonesia (BkkbN), Universitas Gadjah Mada (UGM), Universitas Hasanuddin (UNHAS), Universitas Sumatera Utara (USU), and The Bill & Melinda Gates Institute for Population and Reproductive Health at The Johns Hopkins Bloomberg School of Public Health. Performance Monitoring and Accountability 2020 (PMA2020) Survey Round 2, PMA2016/Indonesia-R2. 2016. Indonesia and Baltimore, Maryland, United States of America.
	2018	United Nations Statistics Division (UNSD). United Nations Demographic Yearbook 2018. New York, United States of America: United Nations (UN), 2019.
Laos	1995	National Statistical Center (Laos). Laos Census 1995. as it appears in United Nations Statistics Division (UNSD). United Nations Demographic Yearbook Special Census Topic, 2000 Round. New York, United States of America: United Nations (UN).
	2005	National Statistical Center (Laos). Laos Reproductive Health Survey 2005.
	2011–2012; 2017	Ministry of Education and Sports (Laos), Ministry of Health (Laos), Ministry of Planning and Investment (Laos), Lao Statistics Bureau, and United Nations Children's Fund (UNICEF). Laos Multiple Indicator Cluster Survey 2011-2012 and 2017. New York, United States of America: United Nations Children's Fund (UNICEF).
Malaysia	1952–1972; 1976–1978; 1984–1986; 1990–1998; 2000–2008; 2012	Malaysia Vital Registration Death Data 1952-1972, 1976-1978, 1984-1986, 1990-1998, 2000-2008, and 2012. as it appears in United Nations Statistics Division (UNSD). United Nations Demographic Yearbook - Historical Supplement 1997; United Nations Demographic Yearbook 2001, 2004, 2006, 2008, 2009-2010, and 2014; and United Nations Demographic Yearbook Database. New York, United States of America: United Nations (UN).
	1970; 1980	Department of Statistics (Malaysia), Minnesota Population Center. Malaysia Population and Housing Census 1970 and 1980 from the Integrated Public Use Microdata Series, International: [Machine-readable database]. Minneapolis: University of Minnesota.
	1974	National Population and Family Development Board (Malaysia), International Statistical Institute. Malaysia World Fertility Survey 1974. Voorburg, Netherlands: International Statistical Institute.
	1980–1982	Department of Statistics (Malaysia). Vital Statistics: Peninsular Malaysia 1980, 1981, and 1982. Kuala Lumpur, Malaysia: Department of Statistics (Malaysia), 1983 and 1984.

Malaysia *(continued from the previous page)*	1980-1999	World Health Organization (WHO). Country Mortality Data 1980-1999.
	2017	World Health Organization (WHO). WHO Mortality Database Version March 2017. Geneva, Switzerland: World Health Organization (WHO).
	1998	Department of Statistics (Malaysia). Malaysia - Peninsular Vital Statistics - Deaths 1998.
	2000–2020	Malaysia Vital Registration - Deaths 2000-2020 ICD10. as it appears in World Health Organization (WHO). WHO Mortality Database (multiple versions: October 2017 and February 2023). Geneva, Switzerland: World Health Organization (WHO).
	2018	Ministry of Health (Malaysia). Malaysia Health Indicators 2018. Putrajaya, Malaysia: Ministry of Health (Malaysia).
	2018	United Nations Statistics Division (UNSD). United Nations Demographic Yearbook 2018. New York, United States of America: United Nations (UN), 2019.
	2019; 2021–2023	Department of Statistics (Malaysia), National Registration Department, Ministry of Home Affairs (Malaysia). Malaysia Vital Statistics 2019, 2021-2023. Putrajaya, Malaysia: Department of Statistics (Malaysia), 2019, 2021-2023.
	2023	Department of Statistics (Malaysia), National Registration Department, Ministry of Home Affairs (Malaysia). Malaysia Demographic Statistics 2023. Putrajaya, Malaysia: Department of Statistics (Malaysia).
Myanmar	1983; 2014	Census Division, Department of Immigration and Manpower (Myanmar). Myanmar Population and Housing Census 1983. as it appears in United Nations Statistics Division (UNSD). United Nations Demographic Yearbook - Historical Supplement 1997. New York, United States of America: United Nations (UN).<br><br>Ministry of Immigration and Population (Myanmar). Myanmar Population and Housing Census 2014.
	1991	Ministry of Immigration and Population (Myanmar), United Nations Population Fund (UNFPA). Myanmar Population Change and Fertility Survey 1991.
	1995; 2009–2010	Ministry of Health (Myanmar), Ministry of National Planning and Economic Development (Myanmar), and United Nations Children's Fund (UNICEF). Myanmar Multiple Indicator Cluster Survey 1995 and 2009-2010.
	1997; 2001; 2006–2007	Ministry of Immigration and Population (Myanmar), United Nations Population Fund (UNFPA). Myanmar Fertility and Reproductive Health Survey 1997, 2001, and 2006-2007.
	2002–2003	Ministry of Health (Myanmar), United Nations Children's Fund (UNICEF). Myanmar Overall and Cause Specific Under-Five Mortality Survey 2002-2003.
	2005	Myanmar Vital Registration Death Data 2005. as it appears in United Nations Statistics Division (UNSD). United Nations Demographic Yearbook 2007. New York, United States of America: United Nations (UN), 2009.
	2009–2017	Central Statistical Office (Myanmar). Myanmar Vital Registration - Deaths by State/Region, Sex, Age, and Cause 2009-2017.
	2015–2016	ICF International, Ministry of Health and Sports (Myanmar). Myanmar Demographic and Health Survey 2015-2016. Fairfax, United States of America: ICF International, 2017.

	2017	World Health Organization (WHO). WHO Mortality Database Version March 2017. Geneva, Switzerland: World Health Organization (WHO).
	2018	United Nations Statistics Division (UNSD). United Nations Demographic Yearbook 2018. New York, United States of America: United Nations (UN), 2019.
	2022	Central Statistical Office (Myanmar). Myanmar Statistical Yearbook 2022. Naypyidaw, Myanmar: Central Statistical Office (Myanmar), 2022.
Philippines	1950–1953; 1956–1972; 1974–1976; 1978–1993	Philippines Vital Registration Death Data 1950-1953, 1956-1972, 1974-1976, and 1978-1993. as it appears in United Nations Statistics Division (UNSD). United Nations Demographic Yearbook - Historical Supplement 1997. New York, United States of America: United Nations (UN).
	1958	Bureau of Census and Statistics (Philippines). Philippines Statistical Survey of Private Households 1958. as it appears in United Nations Statistics Division (UNSD). United Nations Demographic Yearbook - Historical Supplement 1997. New York, United States of America: United Nations (UN).
	1963–1972; 1973–1978; 1981; 1992–1998; 1999–2003	Philippines Vital Registration - Deaths 1963-1972 ICD7, 1973-1978 and 1981 ICD8, 1992-1998 ICD9, and 1999-2003 ICD10. as it appears in World Health Organization (WHO). WHO Mortality Database (multiple versions: July 2012, May 2013, February 2014, November 2015, September 2016, and October 2017). Geneva, Switzerland: World Health Organization (WHO).
	1965; 1968–1974; 1976–1979	Bureau of Census and Statistics (Philippines) and National Statistics Office (Philippines). Philippines Vital Statistics Report 1965, 1968-1974, and 1976-1979. Manila, Philippines: Bureau of Census and Statistics (Philippines)/National Statistics Office (Philippines).
	1970; 1980	Bureau of Census and Statistics (Philippines) and National Census & Statistics Office (Philippines). Philippines Population and Housing Census 1970 and 1980. as it appears in United Nations Statistics Division (UNSD). United Nations Demographic Yearbook - Historical Supplement 1997. New York, United States of America: United Nations (UN).
	1978	National Statistics Office (Philippines), International Statistical Institute. Philippines World Fertility Survey 1978. Voorburg, Netherlands: International Statistical Institute.
	1979–2000; 2001–2005	Department of Health (Philippines). Philippines Vital Statistics - Deaths 1979-2000 and 2001-2005.
	1990; 2010	National Statistics Office (Philippines), Philippines Statistics Authority, and Minnesota Population Center. Philippines Population and Housing Census 1990 and 2010 from the Integrated Public Use Microdata Series, International: [Machine-readable database]/Version x.x [dataset]. Minneapolis, MN: University of Minnesota/IPUMS, 2018. https://doi.org/10.18128/D020.V7.1
	1993; 1998; 2003; 2008; 2013; 2017; 2022	Macro International, Inc/ICF International, National Statistics Office (Philippines), Department of Health (Philippines), Philippines Statistics Authority, and United States Agency for International Development (USAID). Philippines Demographic and Health Survey 1993, 1998, 2003, 2008, 2013, 2017, and 2022. Fairfax, United States of America: ICF International, 2010, 2014, 2018, and 2023.
	1993	Macro International, Inc, National Statistics Office (Philippines). Philippines In Depth Demographic and Health Survey 1993. Fairfax, United States of America: ICF International.

Philippines *(continued from the previous page)*	1997	United Nations Statistics Division (UNSD). United Nations Demographic Yearbook - Historical Supplement 1997. New York, United States of America: United Nations (UN).
	2006–2022	National Statistics Office (Philippines) and Philippines Statistics Authority. Philippines Vital Registration - Deaths 2006-2022.
	2011	ICF International, National Statistics Office (Philippines). Philippines Family Health Survey 2011.
	2014; 2017	World Health Organization (WHO). WHO Mortality Database Version February 2014 and March 2017. Geneva, Switzerland: World Health Organization (WHO).
	2018	United Nations Statistics Division (UNSD). United Nations Demographic Yearbook 2018. New York, United States of America: United Nations (UN), 2019.
	2023	National Statistics Office (Philippines). Philippines Birth, Marriage, and Death Statistics for 2023 (Provisional, as of 31 May 2024). Manila, Philippines: National Statistics Office (Philippines).
Singapore	1950–1997	Singapore Vital Registration Death Data 1950-1997. as it appears in United Nations Statistics Division (UNSD). United Nations Demographic Yearbook - Historical Supplement 1997. New York, United States of America: United Nations (UN).
	1955–1962; 1980–2015; 2021–2022	Singapore Vital Registration - Deaths 1955-1962 ICD7, 1980-2011 ICD9, and 2012-2015 and 2021-2022 ICD10. as it appears in World Health Organization (WHO)/Ministry of Health (Singapore). Singapore Causes of Death 1980-2015/WHO Mortality Database (multiple versions: July 2012, November 2015, and February 2024). Geneva, Switzerland: World Health Organization (WHO).
	1980–2020	Ministry of Health (Singapore). Singapore Causes of Death 1980-2020.
	2017	World Health Organization (WHO). WHO Mortality Database Version March 2017. Geneva, Switzerland: World Health Organization (WHO).
	2018	United Nations Statistics Division (UNSD). United Nations Demographic Yearbook 2018. New York, United States of America: United Nations (UN), 2019.
	2018–2019; 2021–2022	Immigration and Checkpoints Authority (ICA) (Singapore). Singapore Report on Registration of Births and Deaths 2018–2019, 2021–2022. Singapore: Immigration and Checkpoints Authority (ICA) (Singapore), 2019, 2020, 2022, and 2023.
	2023	Immigration and Checkpoints Authority (ICA) (Singapore). Singapore Demographic Bulletin 1st-4th Quarter 2023. Singapore: Immigration and Checkpoints Authority (ICA) (Singapore).
Thailand	1950–1994; 2008–2009	Thailand Vital Registration Death Data 1950-1994, 2008, and 2009. as it appears in United Nations Statistics Division (UNSD). United Nations Demographic Yearbook - Historical Supplement 1997 and United Nations Demographic Yearbook Database. New York, United States of America: United Nations (UN).
	1955–1968; 1969–1978; 1979–1987; 1990–1992; 1994–2019	Thailand Vital Registration - Deaths 1955-1968 ICD7, 1969-1978 ICD8, 1979-1987 and 1990-1992 ICD9, and 1994-2019 ICD10. as it appears in World Health Organization (WHO). WHO Mortality Database (multiple versions: July 2012, May 2013, November 2015, September 2016, October 2017, April 2018, December 2019, and March 2021). Geneva, Switzerland: World Health Organization (WHO).

Thailand *(continued from the previous page)*	1970; 1980; 1990; 2000	National Statistical Office (Thailand), Minnesota Population Center. Thailand Population and Housing Census 1970, 1980, 1990, and 2000 from the Integrated Public Use Microdata Series, International: [Machine-readable database]. Minneapolis: University of Minnesota.
	1975	Institute of Population Studies (Thailand), Chulalongkorn University, International Statistical Institute. Thailand World Fertility Survey 1975. Voorburg, Netherlands: International Statistical Institute.
	1981; 1984	Ministry of Public Health (Thailand). Thailand Contraception Prevalence Survey 1981 and 1984.
	1987	Chulalongkorn University, Institute of Population Studies (Thailand), Westinghouse; Institute for Resource Development. Thailand Demographic and Health Survey 1987. Fairfax, United States of America: ICF International.
	2005–2006	National Statistical Office (Thailand). Thailand Survey of Population Change 2005-2006.
	2005–2006; 2012; 2015–2016	College of Population Studies, Chulalongkorn University (Thailand), Institute for Population and Social Research, Mahidol University (Thailand), International Health Policy Program (Thailand), Ministry of Education (Thailand), Ministry of Public Health (Thailand), Ministry of Social Development and Human Security (MSDHS) (Thailand), National Health Security Office (Thailand), National Statistical Office (Thailand), Thai Health Promotion Foundation, and United Nations Children's Fund (UNICEF). Thailand Multiple Indicator Cluster Survey 2005-2006, 2012, and 2015-2016. New York, United States of America: United Nations Children's Fund (UNICEF).
	2014; 2017	World Health Organization (WHO). WHO Mortality Database Version February 2014 and March 2017. Geneva, Switzerland: World Health Organization (WHO).
	2016	National Health Security Office (Thailand), National Statistical Office (Thailand), United Nations Children's Fund (UNICEF). Thailand - Bangkok Small Community Multiple Indicator Cluster Survey 2016. New York, United States of America: United Nations Children's Fund (UNICEF), 2018.
	2018	United Nations Statistics Division (UNSD). United Nations Demographic Yearbook 2018. New York, United States of America: United Nations (UN), 2019.
		United Nations Statistics Division (UNSD). United Nations Demographic Yearbook Database.
	2020–2022	Department of Provincial Administration (Thailand). Thailand Annual Number of Deaths by Age 2020, 2021, and 2022. Thailand: Department of Provincial Administration (Thailand).
	2023	Department of Provincial Administration (Thailand). Thailand Statistics on Number of Deaths 2023. Thailand: Department of Provincial Administration (Thailand).
Timor-Leste	1991; 1994; 1997	Central Bureau of Statistics (Indonesia), Macro International, Inc, Ministry of Health (Indonesia), National Family Planning Coordinating Board (Indonesia). Indonesia Demographic and Health Survey 1991, 1994, and 1997. Fairfax, United States of America: ICF International.
	2001; 2007–2008	National Statistics Directorate (Timor-Leste), World Bank. Timor-Leste Living Standards and Measurement Survey 2001 and 2007-2008. Washington DC, United States of America: World Bank.
	2003; 2009–2010; 2016	ACIL Australia Pty Ltd., Australian National University, Ministry of Health (Timor-Leste), Ministry of Finance (Timor-Leste), National Statistics Directorate (Timor-Leste), University of Newcastle (Australia), ICF Macro,

Timor-Leste *(continued from the previous page)*		and ICF International. Timor-Leste Demographic and Health Survey 2003, 2009-2010, and 2016. Newcastle, Australia: University of Newcastle (Australia)/Fairfax, United States of America: ICF International, 2018.
	2004	National Statistics Directorate (Timor-Leste). Timor-Leste Population and Housing Census 2004.
Viet Nam	1989; 1999; 2009	General Statistics Office (Viet Nam), Minnesota Population Center. Viet Nam Population Census 1989 and Population and Housing Census 1999 and 2009 from the Integrated Public Use Microdata Series, International: [Machine-readable database]. Minneapolis: University of Minnesota.
	1994	General Statistics Office (Vietnam). Vietnam Intercensal Demographic Survey 1994.
	1997; 2002	Macro International, Inc, National Committee for Population and Family Planning (Vietnam), and General Statistics Office (Vietnam). Vietnam Demographic and Health Survey 1997 and 2002. Calverton/Fairfax, United States of America: Macro International, Inc/ICF International.
	2000; 2006; 2010–2011; 2013–2014; 2020–2021	General Statistics Office (Vietnam), United Nations Children's Fund (UNICEF). Vietnam Multiple Indicator Cluster Survey 2000, 2006, 2010-2011, 2013-2014, and 2020-2021. New York, United States of America: United Nations Children's Fund (UNICEF).
	2001–2002	General Statistics Office (Vietnam), Ministry of Health (Vietnam). Vietnam National Health Survey 2001-2002.
	2005	General Statistics Office (Vietnam), National Institute of Hygiene and Epidemiology (Viet Nam), ORC Macro. Vietnam AIDS Indicator Survey 2005. Fairfax, United States of America: ICF International.
	2006–2008; 2010–2011; 2013	General Statistics Office (Vietnam/Viet Nam), United Nations Population Fund (UNFPA). Vietnam/Viet Nam Population Change and Family Planning Survey 2006-2008, 2010-2011, and 2013. Hanoi, Viet Nam: General Statistics Office (Viet Nam).
	2008	Ministry of Health (Vietnam). Vietnam Health Statistics Yearbook 2008. Hanoi, Viet Nam: Ministry of Health (Vietnam).

Supplementary results

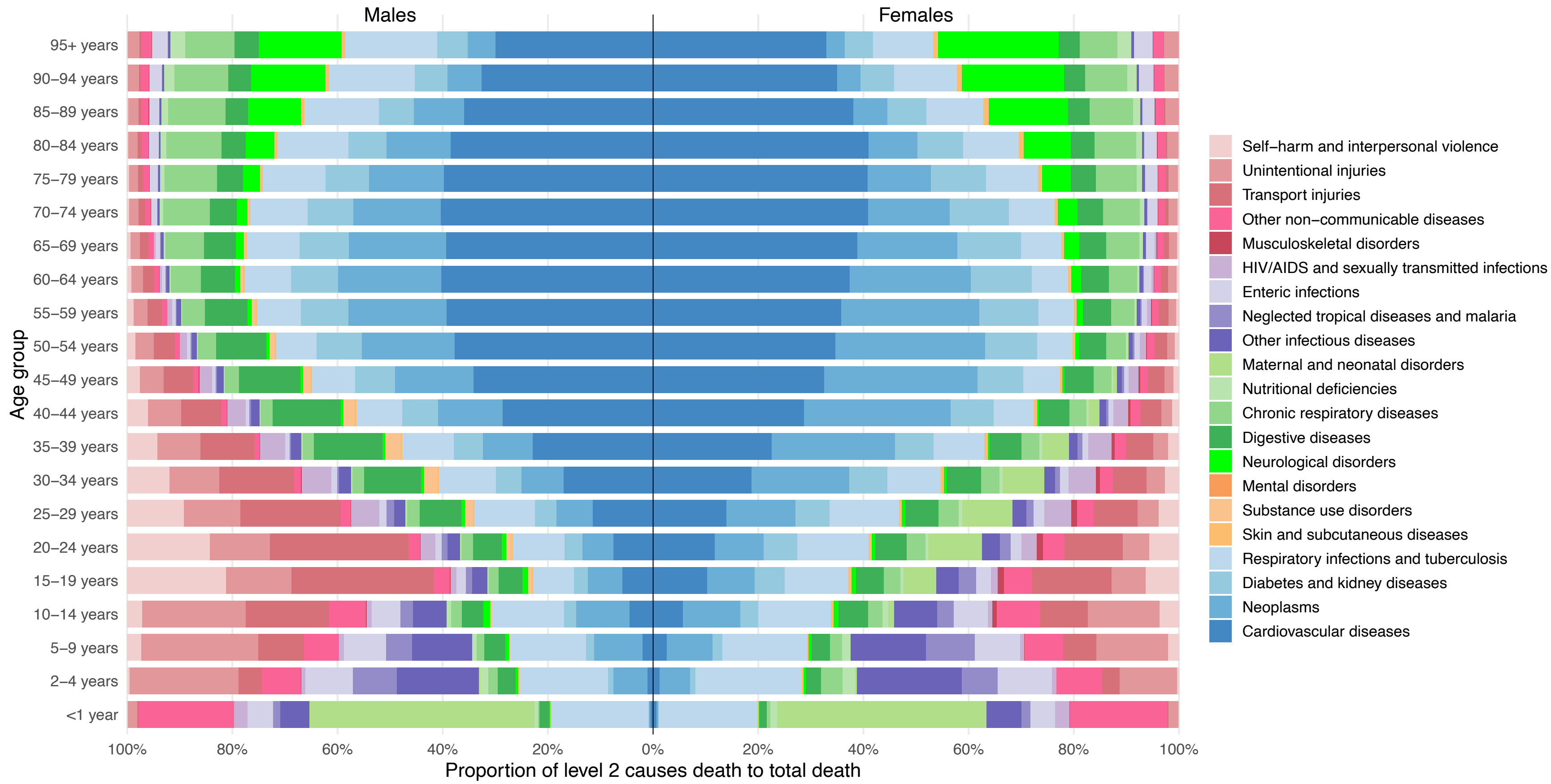

Figure S1 | Relative contribution (%) of Level 2 causes of death to the total mortality by sex and age group, ASEAN, 2023

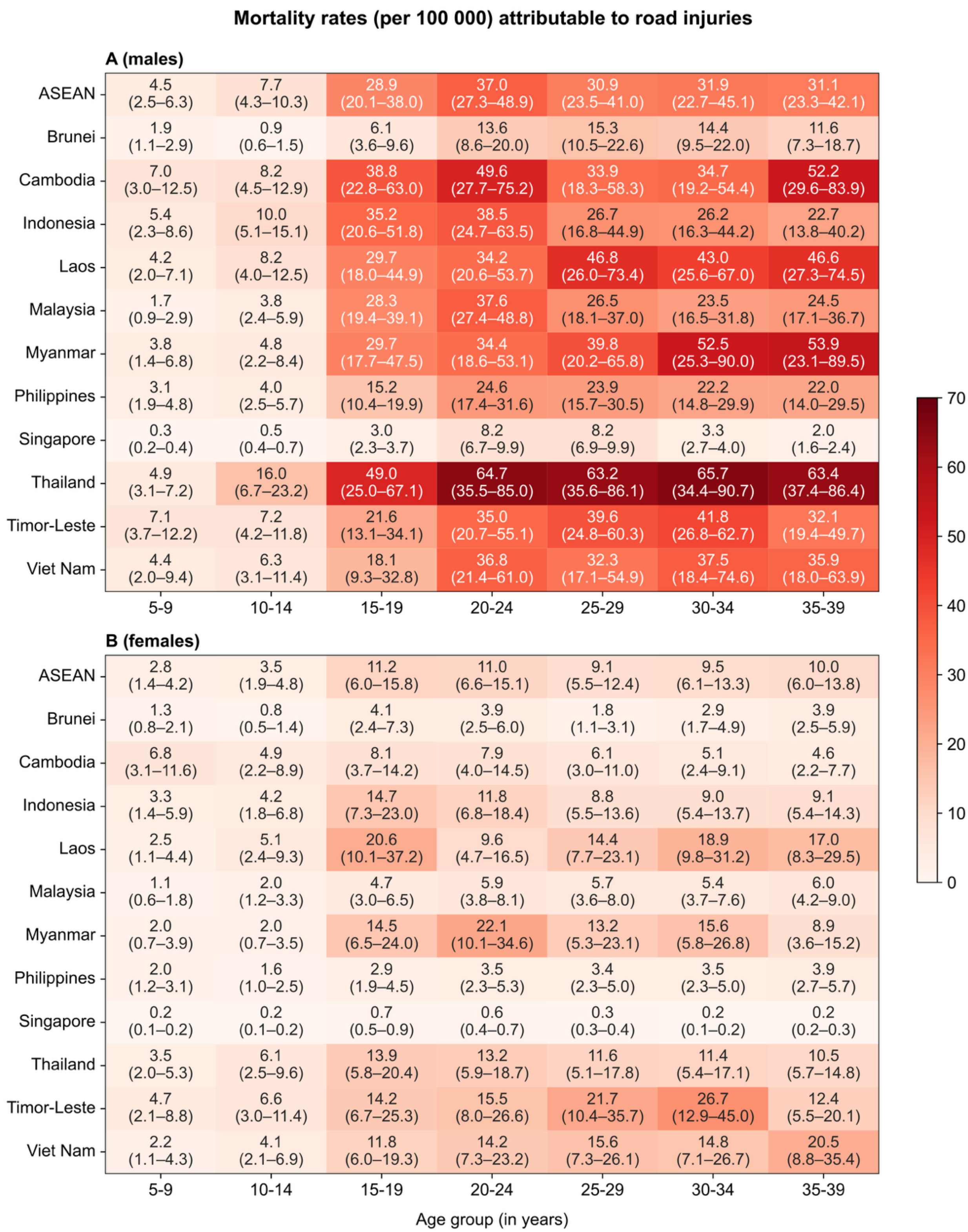

Figure S2 | Mortality rates per 100 000 population (95% UI) attributable to road injuries in ASEAN and its member states for **A**) males and **B**) females, across high-burden age groups (in years), 2023.

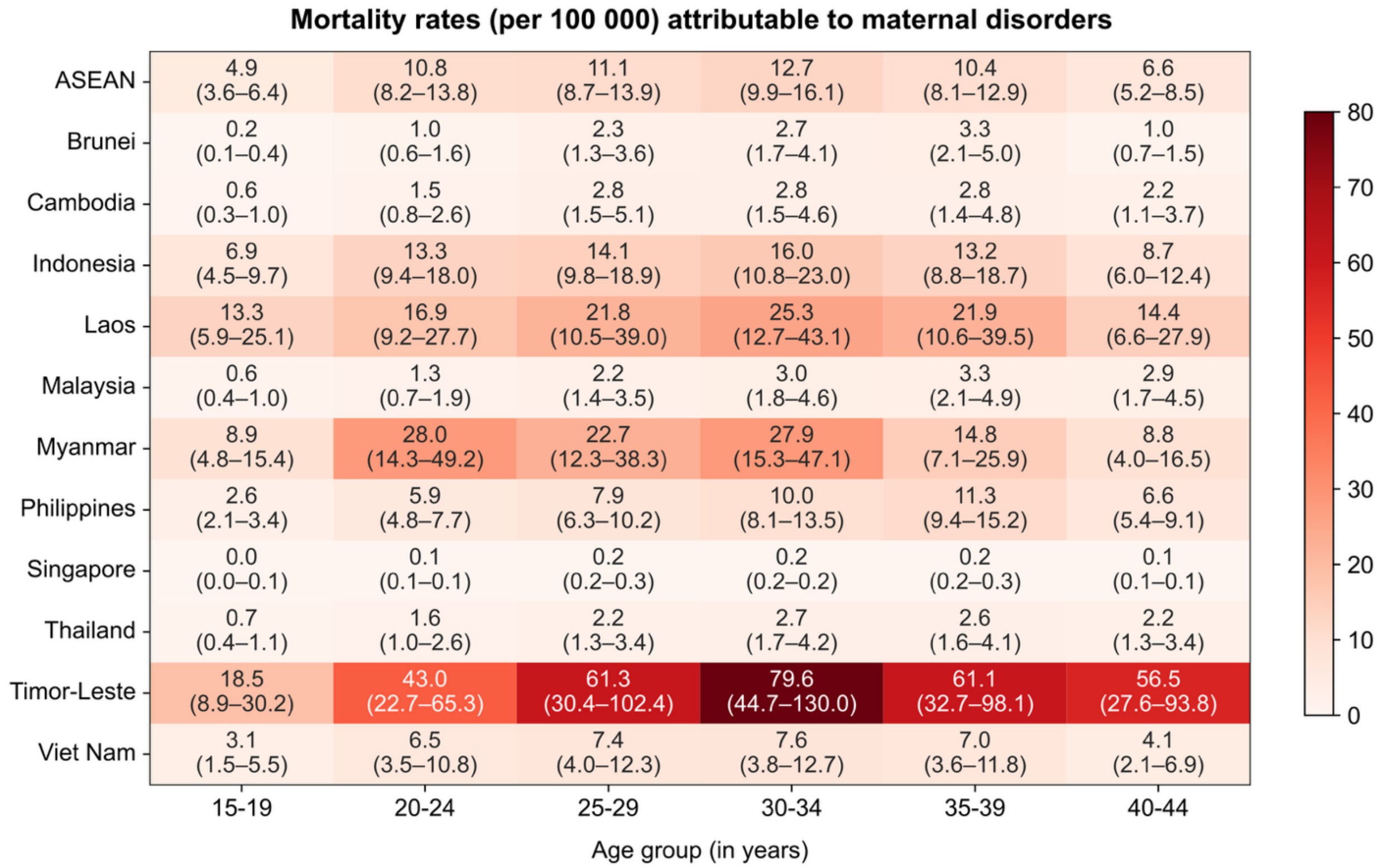

Figure S3 | Mortality rates per 100 000 population (95% UI) attributable to maternal disorders in ASEAN and its member states, 2023.

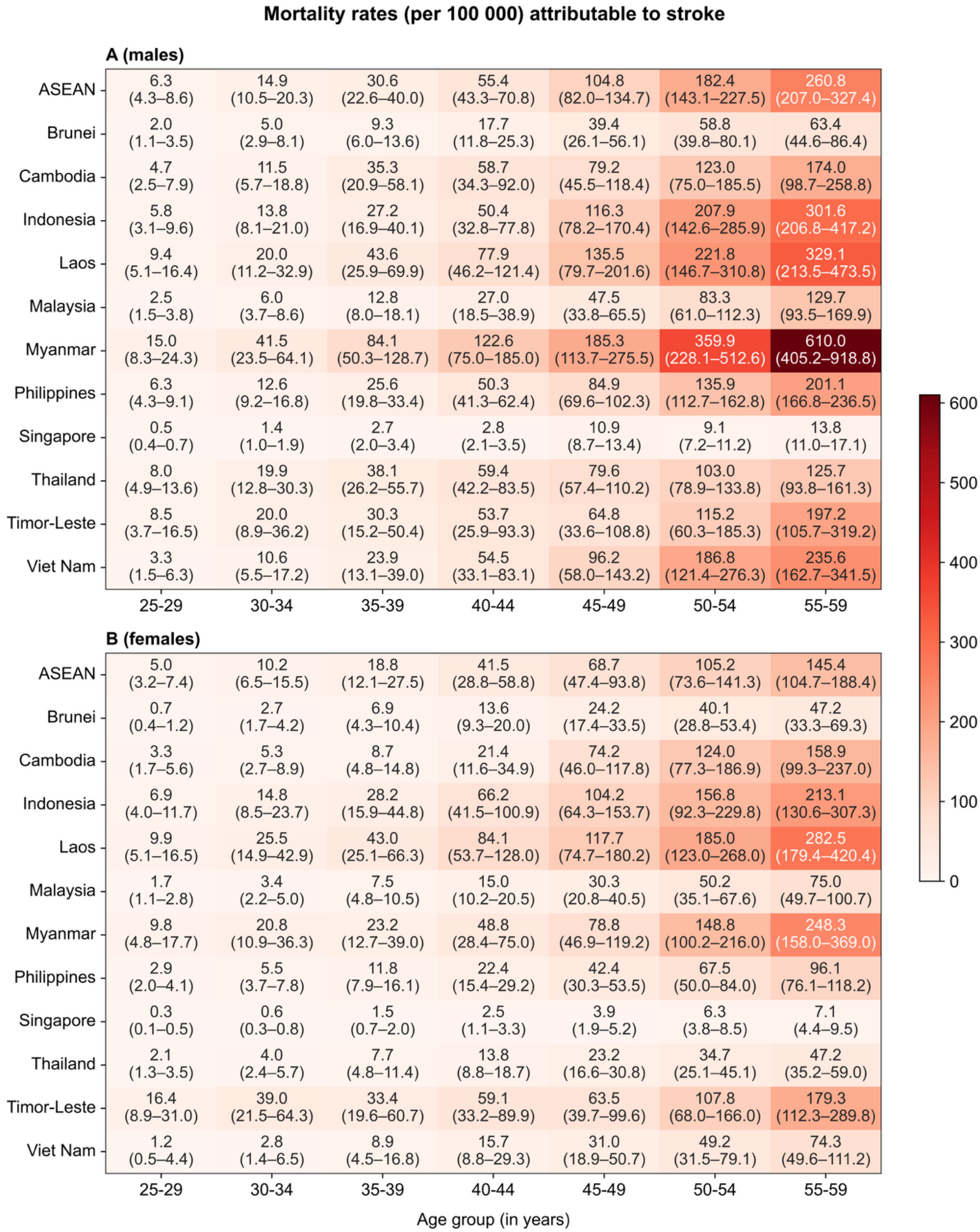

Figure S4 | Mortality rates per 100 000 population (95% UI) attributable to stroke in ASEAN and its member states for **A**) males and **B**) females, among adults aged 25–59 years, 2023.

Table S1 | Sex-disaggregated life expectancy at birth (in years) in 1990 and 2023, and its percentage change from 1990 to 2023, in ASEAN and its member states

Life Expectancy at Birth						
	**Males**			**Females**		
**Location**	**1990<br>(years, 95% UI)**	**2023<br>(years, 95% UI)**	**Percentage change<br>1990-2023<br>(%, 95% UI)**	**1990<br>(year, 95% UI)**	**2023<br>(year, 95% UI)**	**Percentage change<br>1990-2023<br>(%, 95% UI)**
**ASEAN**	62·5<br>(62·2 - 62·9)	70·1<br>(69·6 - 70·7)	12·2	69·1<br>(68·7 - 69·4)	76·2<br>(75·7 - 76·7)	10·3
**Brunei**	70·2<br>(69·9 - 70·6)	76·9<br>(76·2 - 77·5)	9·5	73·8<br>(73·5 - 74·2)	79·4<br>(78·8 - 80·0)	7·6
**Cambodia**	55·2<br>(54·2 - 56·1)	70·8<br>(69·5 - 72·0)	28·3	61·3<br>(60·4 - 62·1)	75·2<br>(74·1 - 76·3)	22·7
**Indonesia**	62·5<br>(61·6 - 63·3)	70·0<br>(68·7 - 71·3)	12·0	67·9<br>(67·0 - 68·8)	75·2<br>(74·0 - 76·4)	10·8
**Laos**	48·9<br>(47·9 - 50·1)	65·2<br>(63·9 - 66·5)	33·3	52·7<br>(51·6 - 53·8)	67·2<br>(66·0 - 68·4)	27·5
**Malaysia**	69·3<br>(68·9 - 69·7)	74·6<br>(74·2 - 75·1)	7·6	73·7<br>(73·3 - 74·1)	78·5<br>(78·1 - 78·9)	6·5
**Myanmar**	51·0<br>(50·1 - 51·9)	61·6<br>(60·1 - 63·0)	20·8	59·2<br>(58·3- 60·0)	69·4<br>(68·2 - 70·5)	17·2
**Philippines**	65·6<br>(65·4 - 65·9)	68·9<br>(68·6 - 69·2)	5·0	72·2<br>(71·9 - 72·6)	75·4<br>(75·1 - 75·6)	4·4
**Singapore**	72·9<br>(72·7 - 73·1)	83·1<br>(82·9 - 83·4)	14·0	78·3<br>(78·1 - 78·5)	87·5<br>(87·3 - 87·8)	11·7
**Thailand**	68·6<br>(68·4 - 68·8)	73·7<br>(73·4 - 73·9)	7·4	75·3<br>(75·2 - 75·5)	81·4<br>(81·2 - 81·7)	8·1
**Timor-Leste**	56·8<br>(56·0 - 57·7)	69·6<br>(68·4 - 70·9)	22·5	58·2<br>(57·2 - 59·0)	70·1<br>(68·9 - 71·3)	20·5
**Viet Nam**	64·9<br>(64·3 - 65·4)	71·9<br>(70·6 - 73·2)	10·8	73·5<br>(72·9 - 74·1)	80·2<br>(79·0 - 81·5)	9·1

95% UI=95% Uncertainty Interval. ASEAN=Association of Southeast Asian Nations. LE= Life Expectancy

Table S2 | Sex-disaggregated all-cause probability of death from birth to age 70 years (70q0) in 1990 and 2023, and its percentage change from 1990 to 2023, in ASEAN and its member states

All-cause probability of death from birth to age 70 years						
	Males			Females		
Location	1990 (%)	2023 (%)	Percentage change 1990-2023 (%)	1990 (%)	2023 (%)	Percentage change 1990-2023 (%)
ASEAN	52·1	40·3	-22·6	36·2	26·9	-25·7
Brunei	39·3	25·3	-35·6	31·2	18·5	-40·7
Cambodia	58·1	36·9	-36·4	46·2	27·4	-40·7
Indonesia	52·6	41·6	-21·0	39·2	30·6	-21·9
Laos	69·5	48·8	-29·8	60·2	42·6	-29·2
Malaysia	42·3	32·2	-23·9	30·6	21·2	-30·7
Myanmar	72·8	56·9	-21·8	51·3	37·3	-27·3
Philippines	45·0	41·6	-7·6	28·7	26·3	-8·4
Singapore	35·0	13·4	-61·7	21·5	7·5	-65·1
Thailand	41·5	34·3	-17·3	26·7	17·6	-34·1
Timor-Leste	56·1	38·4	-31·5	50·7	35·4	-30·2
Viet Nam	48·0	36·4	-24·2	28·8	20·5	-28·8

ASEAN=Association of Southeast Asian Nations

Table S3 | Relative contribution (%) of top five Level 3 causes of death to the total mortality by sex and age groups in ASEAN, 2023

Age group (in years)	Males					Females				
	Top 1	Top 2	Top 3	Top 4	Top 5	Top 1	Top 2	Top 3	Top 4	Top 5
<1	Neonatal disorders 42·9% (36·7 - 49·1)	Congenital birth defects 16·7% (11·0 - 24·0)	Lower respiratory infections 12·2% (8·0 - 18·5)	COVID-19 5·6% (4·7 - 6·7)	Diarrheal diseases 4·7% (2·0 - 9·2)	Neonatal disorders 39·8% (34·1 - 45·8)	Congenital birth defects 18·1% (12·1 - 25·1)	Lower respiratory infections 13·2% (8·6 - 18·3)	COVID-19 4·9% (4·0 - 6·1)	Diarrheal diseases 4·4% (2·0 - 8·7)
2-4	Drowning 15·5% (7·5 - 22·3)	Lower respiratory infections 10·5% (7·2 - 15·2)	Dengue 6·9% (1·8 - 16·1)	Congenital birth defects 6·7% (4·6 - 10·0)	Pertussis 4·8% (1·7 - 9·1)	Lower respiratory infections 15·0% (10·2 - 19·7)	Congenital birth defects 7·8% (5·0 - 11·2)	Pertussis 7·4% (3·1 - 14·1)	Drowning 6·9% (4·5 - 10·1)	Diarrheal diseases 5·4% (2·9 - 9·2)
5-9	Drowning 15·6% (9·6 - 21·1)	Lower respiratory infections 9·1% (6·6 - 12·4)	Road injuries 8·1% (4·4 - 11·6)	Congenital birth defects 5·5% (4·0 - 7·7)	Typhoid and paratyphoid 4·8% (2·2 - 8·5)	Lower respiratory infections 9·6% (6·5 - 14·5)	Drowning 9·0% (6·0 - 12·4)	Dengue 8·0% (2·6 - 19·3)	Congenital birth defects 6·2% (4·3 - 8·2)	Road injuries 6·0% (3·2 - 8·9)
10-14	Road injuries 14·9% (8·5 - 19·9)	Drowning 11·2% (8·0 - 15·0)	Lower respiratory infections 6·5% (4·9 - 8·5)	Congenital birth defects 5·5% (4·0 - 7·6)	Leukemia 4·7% (3·5 - 6·5)	Drowning 8·7% (6·2 - 11·4)	Road injuries 8·6% (4·7 - 11·7)	Lower respiratory infections 6·9% (4·9 - 9·7)	Congenital birth defects 6·4% (4·3 - 8·6)	Leukemia 5·1% (3·9 - 6·7)
15-19	Road injuries 25·0% (17·0 - 31·0)	Conflict and terrorism 10·6% (4·5 - 21·2)	Drowning 4·6% (3·0 - 6·9)	Self-harm 4·2% (2·6 - 5·8)	Interpersonal violence 4·0% (2·7 - 6·1)	Road injuries 14·4% (8·2 - 19·9)	Maternal disorders 6·3% (5·1 - 7·9)	Tuberculosis 5·9% (3·8 - 8·3)	Chronic kidney disease 4·3% (2·6 - 6·1)	Lower respiratory infections 4·0% (2·6 - 6·3)
20-24	Road injuries 24·3% (18·2 - 30·1)	Conflict and terrorism 6·2% (2·6 - 13·3)	Tuberculosis 5·1% (3·4 - 7·3)	Self-harm 5·0% (3·3 - 6·5)	Interpersonal violence 4·5% (3·4 - 6·3)	Road injuries 10·6% (6·3 - 14·1)	Maternal disorders 10·3% (8·5 - 12·5)	Tuberculosis 6·8% (4·7 - 9·2)	Chronic kidney disease 5·0% (3·1 - 7·0)	Ischemic heart disease 4·5% (2·9 - 6·6)
25-29	Road injuries 17·7% (13·5 - 22·2)	Tuberculosis 6·4% (4·5 - 9·2)	Ischemic heart disease 5·5% (4·1 - 7·5)	Cirrhosis and other chronic liver diseases 5·4% (3·9 - 7·2)	HIV/AIDS 5·3% (2·6 - 8·3)	Maternal disorders 9·6% (8·1 - 11·4)	Road injuries 7·9% (4·9 - 10·5)	Tuberculosis 6·9% (4·8 - 9·3)	Ischemic heart disease 5·6% (3·6 - 8·1)	HIV/AIDS 5·1% (2·7 - 7·9)
30-34	Road injuries 13·1% (9·2 - 18·1)	Ischemic heart disease 8·3% (6·1 - 10·8)	Cirrhosis and other chronic liver diseases 8·1% (5·9 - 10·7)	Stroke 6·1% (4·4 - 8·1)	Tuberculosis 5·9% (4·3 - 8·3)	Maternal disorders 8·0% (6·7 - 9·5)	Ischemic heart disease 7·9% (5·2 - 11·1)	Stroke 6·5% (4·2 - 9·3)	Road injuries 6·0% (3·7 - 8·2)	Tuberculosis 5·4% (3·8 - 7·3)

Age group (in years)	Males					Females				
	Top 1	Top 2	Top 3	Top 4	Top 5	Top 1	Top 2	Top 3	Top 4	Top 5
35-39	Ischemic heart disease 10·7% (8·3 - 13·5)	Cirrhosis and other chronic liver diseases 10·0% (7·7 - 12·6)	Road injuries 9·4% (7·0 - 12·4)	Stroke 9·2% (7·1 - 11·8)	Tuberculosis 5·2% (3·8 - 7·5)	Stroke 9·3% (6·6 - 12·8)	Ischemic heart disease 9·0% (6·1 - 12·3)	Breast cancer 7·9% (6·2 - 9·8)	Maternal disorders 5·1% (4·2 - 6·1)	Chronic kidney disease 5·0% (3·2 - 6·5)
40-44	Ischemic heart disease 13·3% (10·8 - 16·4)	Stroke 12·2% (9·9 - 15·1)	Cirrhosis and other chronic liver diseases 10·4% (8·4 - 12·9)	Road injuries 7·0% (5·0 - 9·5)	Tuberculosis 4·5% (3·2 - 6·2)	Stroke 13·0% (9·7 - 17·1)	Ischemic heart disease 11·4% (7·7 - 15·2)	Breast cancer 9·9% (7·7 - 12·3)	Chronic kidney disease 5·1% (3·6 - 6·5)	Cirrhosis and other chronic liver diseases 4·5% (3·3 - 6·3)
45-49	Ischemic heart disease 15·6% (12·9 - 19·0)	Stroke 15·2% (12·5 - 18·4)	Cirrhosis and other chronic liver diseases 9·5% (7·6 - 11·5)	Road injuries 5·2% (3·7 - 7·2)	Tuberculosis 4·1% (3·0 - 5·6)	Stroke 16·0% (11·8 - 20·3)	Ischemic heart disease 12·5% (8·9 - 16·3)	Breast cancer 9·9% (8·0 - 12·1)	Chronic kidney disease 4·7% (3·6 - 6·0)	Cirrhosis and other chronic liver diseases 4·2% (3·1 - 5·8)
50-54	Stroke 17·5% (14·7 - 20·7)	Ischemic heart disease 16·6% (14·0 - 19·8)	Cirrhosis and other chronic liver diseases 7·8% (6·3 - 9·3)	Diabetes mellitus 4·8% (3·5 - 6·5)	Tracheal, bronchus, and lung cancer 4·1% (3·2 - 5·4)	Stroke 17·8% (13·4 - 22·6)	Ischemic heart disease 13·1% (9·3 - 16·7)	Breast cancer 8·6% (7·0 - 10·3)	Diabetes mellitus 5·2% (3·8 - 7·3)	Chronic kidney disease 4·7% (3·6 - 6·0)
55-59	Stroke 18·4% (15·3 - 22·0)	Ischemic heart disease 17·2% (14·3 - 20·3)	Cirrhosis and other chronic liver diseases 6·3% (4·9 - 7·6)	Diabetes mellitus 5·3% (3·7 - 7·3)	Tracheal, bronchus, and lung cancer 4·6% (3·6 - 5·9)	Stroke 18·1% (13·7 - 22·1)	Ischemic heart disease 13·6% (10·1 - 17·0)	Breast cancer 7·0% (5·9 - 8·4)	Diabetes mellitus 6·5% (4·8 - 8·8)	Chronic kidney disease 4·8% (3·7 - 6·2)
60-64	Stroke 19·8% (16·7 - 23·2)	Ischemic heart disease 16·5% (13·7 - 19·7)	Diabetes mellitus 5·4% (4·0 - 7·3)	Tracheal, bronchus, and lung cancer 5·3% (4·0 - 6·9)	Cirrhosis and other chronic liver diseases 4·8% (3·8 - 6·0)	Stroke 19·3% (15·3 - 23·9)	Ischemic heart disease 13·7% (10·2 - 17·3)	Diabetes mellitus 7·4% (5·6 - 9·8)	Breast cancer 4·8% (3·9 - 5·7)	Chronic kidney disease 4·2% (3·2 - 5·9)
65-69	Stroke 19·7% (16·7 - 22·9)	Ischemic heart disease 15·7% (13·1 - 18·6)	Chronic obstructive pulmonary disease 6·0% (4·6 - 7·5)	Diabetes mellitus 6·0% (4·5 - 8·0)	Tracheal, bronchus, and lung cancer 5·2% (4·1 - 6·8)	Stroke 20·4% (16·4 - 24·5)	Ischemic heart disease 14·1% (11·0 - 17·1)	Diabetes mellitus 8·0% (5·9 - 10·6)	Chronic obstructive pulmonary disease 4·3% (2·3 - 6·3)	Chronic kidney disease 4·2% (3·2 - 5·6)
70-74	Stroke 20·6% (17·5 - 23·8)	Ischemic heart disease	Chronic obstructive pulmonary disease	Diabetes mellitus 5·5% (4·1 - 7·3)	Lower respiratory infections	Stroke 21·6% (17·6 - 25·6)	Ischemic heart disease	Diabetes mellitus 7·5% (5·7 - 10·3)	Lower respiratory infections	Chronic obstructive pulmonary disease

Age group (in years)	Males					Females				
	Top 1	Top 2	Top 3	Top 4	Top 5	Top 1	Top 2	Top 3	Top 4	Top 5
		15·4% (13·0 - 18·2)	7·3% (5·7 - 9·1)		5·3% (4·4 - 6·3)		14·2% (11·0 - 17·4)		5·0% (3·9 - 6·3)	4·9% (2·7 - 7·7)
75-79	Stroke 20·6% (17·3 - 23·7)	Ischemic heart disease 14·7% (12·4 - 17·5)	Chronic obstructive pulmonary disease 8·3% (6·6 - 10·1)	Lower respiratory infections 6·5% (5·5 - 7·7)	Diabetes mellitus 5·2% (3·9 - 6·9)	Stroke 21·2% (18·1 - 24·8)	Ischemic heart disease 14·3% (11·0 - 17·6)	Diabetes mellitus 6·9% (5·0 - 9·1)	Lower respiratory infections 6·4% (5·0 - 7·9)	Chronic obstructive pulmonary disease 5·5% (3·4 - 8·8)
80-84	Stroke 19·2% (15·9 - 23·0)	Ischemic heart disease 14·6% (12·0 - 17·7)	Chronic obstructive pulmonary disease 8·9% (7·1 - 10·8)	Lower respiratory infections 8·0% (6·7 - 9·6)	Diabetes mellitus 4·2% (3·2 - 5·7)	Stroke 20·1% (16·4 - 24·6)	Ischemic heart disease 14·7% (11·3 - 18·3)	Alzheimer's disease and other dementias 7·6% (1·9 - 19·1)	Lower respiratory infections 7·6% (6·0 - 9·6)	Chronic obstructive pulmonary disease 5·7% (3·3 - 9·2)
85-89	Stroke 16·2% (12·9 - 19·8)	Ischemic heart disease 14·5% (11·6 - 17·9)	Chronic obstructive pulmonary disease 9·3% (7·2 - 11·4)	Lower respiratory infections 9·2% (7·7 - 11·1)	Alzheimer's disease and other dementias 6·1% (1·4 - 16·9)	Stroke 17·3% (13·1 - 21·7)	Ischemic heart disease 14·2% (10·8 - 18·0)	Alzheimer's disease and other dementias 10·5% (2·8 - 26·8)	Lower respiratory infections 8·4% (6·3 - 10·7)	Chronic obstructive pulmonary disease 6·2% (3·4 - 9·4)
90-94	Ischemic heart disease 13·9% (10·4 - 18·1)	Stroke 13·1% (10·0 - 16·8)	Lower respiratory infections 10·1% (8·2 - 12·6)	Alzheimer's disease and other dementias 9·5% (2·3 - 25·2)	Chronic obstructive pulmonary disease 8·9% (6·4 - 11·3)	Alzheimer's disease and other dementias 14·8% (4·0 - 35·8)	Stroke 14·4% (9·9 - 18·9)	Ischemic heart disease 13·3% (9·2 - 17·4)	Lower respiratory infections 9·3% (6·7 - 12·2)	Chronic obstructive pulmonary disease 6·0% (3·3 - 10·3)
95+	Ischemic heart disease 13·4% (9·0 - 18·1)	Alzheimer's disease and other dementias 12·1% (2·7 - 31·0)	Stroke 11·4% (7·9 - 15·4)	Lower respiratory infections 10·4% (8·1 - 13·2)	Chronic obstructive pulmonary disease 7·9% (5·4 - 10·4)	Alzheimer's disease and other dementias 18·7% (4·8 - 43·9)	Stroke 13·0% (7·9 - 19·7)	Ischemic heart disease 12·0% (7·7 - 16·7)	Lower respiratory infections 8·5% (5·8 - 11·6)	Chronic obstructive pulmonary disease 5·3% (2·9 - 9·0)

Table S4 | Absolute number of top five Level 3 causes of death by sex and age groups in ASEAN, 2023

	Males					Females				
Age group	Top 1	Top 2	Top 3	Top 4	Top 5	Top 1	Top 2	Top 3	Top 4	Top 5
<1	Neonatal disorders<br>51 200<br>(42 700–60 300)	Congenital birth defects<br>19 900<br>(13 100–28 400)	Lower respiratory infections<br>14 500<br>(9 170–21 800)	COVID-19<br>6 680<br>(5 480–8 200)	Diarrheal diseases<br>5 610<br>(2 300–10 700)	Neonatal disorders<br>34 300<br>(28 900–42 200)	Congenital birth defects<br>15 600<br>(10 400–22 300)	Lower respiratory infections<br>11 300<br>(7 350–15 500)	COVID-19<br>4 230<br>(3 360–5 370)	Diarrheal diseases<br>3 810<br>(1 670–7 420)
2-4	Drowning<br>2 940<br>(1 390–4 360)	Lower respiratory infections<br>2 000<br>(1 320–3 010)	Dengue<br>1 330<br>(320–3 380)	Congenital birth defects<br>1 280<br>(874–1 880)	Pertussis<br>907<br>(343–1 890)	Lower respiratory infections<br>2 010<br>(1 330–2 750)	Congenital birth defects<br>1 050<br>(677–1 580)	Pertussis<br>996<br>(411–1 890)	Drowning<br>918<br>(588–1 330)	Diarrheal diseases<br>722<br>(374–1 250)
5-9	Drowning<br>2 490<br>(1 500–3 340)	Lower respiratory infections<br>1 450<br>(1 010–2 020)	Road injuries<br>1 300<br>(715–1 840)	Congenital birth defects<br>880<br>(618–1 250)	Typhoid and paratyphoid<br>763<br>(357–1 350)	Lower respiratory infections<br>1 210<br>(791–1 810)	Drowning<br>1 120<br>(746–1 600)	Dengue<br>999<br>(313–2 430)	Congenital birth defects<br>771<br>(526–1 070)	Road injuries<br>754<br>(391–1 150)
10-14	Road injuries<br>2 260<br>(1 270–3 040)	Drowning<br>1 690<br>(1 190–2 200)	Lower respiratory infections<br>984<br>(733–1 310)	Congenital birth defects<br>833<br>(577–1 180)	Leukemia<br>719<br>(515–1 000)	Drowning<br>970<br>(672–1 280)	Road injuries<br>960<br>(518–1 350)	Lower respiratory infections<br>763<br>(556–1 080)	Congenital birth defects<br>707<br>(483–982)	Leukemia<br>564<br>(418–790)
15-19	Road injuries<br>8 230<br>(5 740–10 800)	Conflict and terrorism<br>3 550<br>(1 450–7 520)	Drowning<br>1 490<br>(1 000–2 120)	Self-harm<br>1 370<br>(859–1 950)	Interpersonal violence<br>1 300<br>(849–2 030)	Road injuries<br>3 040<br>(1 630–4 270)	Maternal disorders<br>1 330<br>(984–1 740)	Tuberculosis<br>1 240<br>(773–1 800)	Chronic kidney disease<br>905<br>(539–1 310)	Lower respiratory infections<br>846<br>(564–1 310)
20-24	Road injuries<br>10 400<br>(7 680–13 700)	Conflict and terrorism<br>2 670<br>(1 090–5 650)	Tuberculosis<br>2 170<br>(1 420–3 220)	Self-harm<br>2 150<br>(1 420–2 860)	Interpersonal violence<br>1 940<br>(1 430–2 640)	Road injuries<br>2 970<br>(1 790–4 060)	Maternal disorders<br>2 890<br>(2 220–3 710)	Tuberculosis<br>1 910<br>(1 240–2 780)	Chronic kidney disease<br>1 400<br>(863–2 020)	Ischemic heart disease<br>1 280<br>(766–1 930)
25-29	Road injuries<br>8 720<br>(6 640–11 600)	Tuberculosis<br>3 160<br>(2 200–4 640)	Ischemic heart disease<br>2 730<br>(1 980–3 730)	Cirrhosis and other chronic liver diseases<br>2 670<br>(1 830–3 640)	HIV/AIDS<br>2 630<br>(1 270–4 080)	Maternal disorders<br>3 020<br>(2 370–3 770)	Road injuries<br>2 490<br>(1 500–3 380)	Tuberculosis<br>2 190<br>(1 410–3 080)	Ischemic heart disease<br>1 750<br>(1 120–2 620)	HIV/AIDS<br>1 600<br>(858–2 520)
30-34	Road injuries<br>8 930<br>(6 350–12 600)	Ischemic heart disease<br>5 660<br>(4 110–7 640)	Cirrhosis and other chronic liver diseases<br>5 520<br>(3 940–7 690)	Stroke<br>4 170<br>(2 950–5 690)	Tuberculosis<br>4 030<br>(2 770–5 850)	Maternal disorders<br>3 430<br>(2 690–4 360)	Ischemic heart disease<br>3 360<br>(2 120–4 850)	Stroke<br>2 760<br>(1 770–4 200)	Road injuries<br>2 570<br>(1 640–3 590)	Tuberculosis<br>2 320<br>(1 540–3 180)
35-39	Ischemic heart disease	Cirrhosis and other chronic liver diseases	Road injuries<br>8 190<br>(6 140–11 100)	Stroke<br>8 070<br>(5 970–10 500)	Tuberculosis<br>4 550<br>(3 220–6 330)	Stroke<br>4 840<br>(3 110–7 070)	Ischemic heart disease	Breast cancer<br>4 100<br>(3 100–5 190)	Maternal disorders<br>2 670<br>(2 090–3 320)	Chronic kidney disease

Age group	Males Top 1	Males Top 2	Males Top 3	Males Top 4	Males Top 5	Females Top 1	Females Top 2	Females Top 3	Females Top 4	Females Top 5
	9 300 (6 910–12 100)	8 690 (6 610–11 200)					4 680 (3 010–6 870)			2 580 (1 610–3 490)
40-44	Ischemic heart disease 15 000 (11 600–18 900)	Stroke 13 800 (10 800–17 600)	Cirrhosis and other chronic liver diseases 11 800 (8 860–15 300)	Road injuries 7 900 (5 440–10 900)	Tuberculosis 5 030 (3 420–6 920)	Stroke 10 200 (7 070–14 400)	Ischemic heart disease 8 920 (5 750–12 700)	Breast cancer 7 680 (5 890–10 100)	Chronic kidney disease 3 950 (2 760–5 400)	Cirrhosis and other chronic liver diseases 3 530 (2 440–5 120)
45-49	Ischemic heart disease 24 000 (19 000–31 500)	Stroke 23 300 (18 200–29 900)	Cirrhosis and other chronic liver diseases 14 700 (11 300–18 800)	Road injuries 7 980 (5 280–11 100)	Tuberculosis 6 300 (4 500–8 780)	Stroke 15 300 (10 600–21 000)	Ischemic heart disease 12 000 (8 060–16 100)	Breast cancer 9 470 (7 440–12 100)	Chronic kidney disease 4 480 (3 320–5 970)	Cirrhosis and other chronic liver diseases 4 050 (2 780–5 780)
50-54	Stroke 36 600 (28 700–45 600)	Ischemic heart disease 34 800 (27 200–43 400)	Cirrhosis and other chronic liver diseases 16 200 (12 700–20 400)	Diabetes mellitus 10 000 (6 980–14 100)	Tracheal, bronchus, and lung cancer 8 630 (6 550–11 200)	Stroke 21 500 (15 100–28 900)	Ischemic heart disease 15 800 (11 000–21 600)	Breast cancer 10 400 (8 000–12 900)	Diabetes mellitus 6 290 (4 270–8 760)	Chronic kidney disease 5 630 (4 250–7 570)
55-59	Stroke 45 100 (35 800–56 600)	Ischemic heart disease 42 200 (33 300–52 300)	Cirrhosis and other chronic liver diseases 15 400 (11 600–19 700)	Diabetes mellitus 12 900 (9 130–19 000)	Tracheal, bronchus, and lung cancer 11 400 (8 630–15 200)	Stroke 26 500 (19 100–34 300)	Ischemic heart disease 19 900 (14 300–27 000)	Breast cancer 10 300 (8 320–12 800)	Diabetes mellitus 9 530 (6 840–12 900)	Chronic kidney disease 7 010 (5 180–9 420)
60-64	Stroke 59 900 (47 300–75 300)	Ischemic heart disease 49 900 (39 100–61 900)	Diabetes mellitus 16 500 (11 700–22 700)	Tracheal, bronchus, and lung cancer 16 200 (12 000–21 500)	Cirrhosis and other chronic liver diseases 14 600 (10 800–19 100)	Stroke 36 300 (27 100–47 500)	Ischemic heart disease 25 700 (19 000–34 100)	Diabetes mellitus 13 800 (10 200–19 000)	Breast cancer 8 920 (7 230–11 100)	Chronic kidney disease 7 870 (5 870–11 000)
65-69	Stroke 59 100 (47 000–72 600)	Ischemic heart disease 47 100 (38 400–59 100)	Chronic obstructive pulmonary disease 17 900 (13 600–23 500)	Diabetes mellitus 17 900 (12 500–25 000)	Tracheal, bronchus, and lung cancer 15 600 (12 000–20 800)	Stroke 41 300 (31 700–51 800)	Ischemic heart disease 28 500 (22 200–35 700)	Diabetes mellitus 16 200 (11 900–22 200)	Chronic obstructive pulmonary disease 8 690 (4 480–13 800)	Chronic kidney disease 8 470 (6 410–11 700)
70-74	Stroke 59 100 (47 900–73 000)	Ischemic heart disease 44 200 (36 500–54 200)	Chronic obstructive pulmonary disease 21 000 (15 400–27 400)	Diabetes mellitus 15 700 (11 200–22 100)	Lower respiratory infections 15 200 (12 200–18 800)	Stroke 46 000 (36 300–56 400)	Ischemic heart disease 30 300 (23 600–38 400)	Diabetes mellitus 16 000 (11 800–21 800)	Lower respiratory infections 10 600 (7 950–13 600)	Chronic obstructive pulmonary disease 10 400 (5 860–16 300)
75-79	Stroke 45 100 (36 900–53 900)	Ischemic heart disease 32 400 (26 100–39 800)	Chronic obstructive pulmonary disease 18 200 (14 000–23 100)	Lower respiratory infections 14 300 (12 200–17 000)	Diabetes mellitus 11 400 (8 410–15 500)	Stroke 40 300 (33 300–49 500)	Ischemic heart disease 27 100 (21 300–33 700)	Diabetes mellitus 13 100 (9 280–18 500)	Lower respiratory infections 12 100 (9 270–14 800)	Chronic obstructive pulmonary disease 10 500 (6 470–17 300)

Age group	Males					Females				
	Top 1	Top 2	Top 3	Top 4	Top 5	Top 1	Top 2	Top 3	Top 4	Top 5
80-84	Stroke 33 700 (26 700–42 100)	Ischemic heart disease 25 600 (19 900–32 600)	Chronic obstructive pulmonary disease 15 700 (12 400–19 700)	Lower respiratory infections 14 100 (12 000–16 800)	Diabetes mellitus 7 420 (5 270–10 300)	Stroke 39 200 (31 600–48 800)	Ischemic heart disease 28 600 (21 900–35 700)	Alzheimer's disease and other dementias 14 900 (3 570–37 900)	Lower respiratory infections 14 800 (12 000–18 500)	Chronic obstructive pulmonary disease 11 100 (6 260–18 000)
85-89	Stroke 18 800 (14 500–23 400)	Ischemic heart disease 16 900 (13 200–21 600)	Chronic obstructive pulmonary disease 10 800 (8 230–13 400)	Lower respiratory infections 10 700 (9 060–12 900)	Alzheimer's disease and other dementias 7 050 (1 610–20 300)	Stroke 29 400 (21 600–37 900)	Ischemic heart disease 24 100 (18 300–30 900)	Alzheimer's disease and other dementias 17 900 (4 730–45 900)	Lower respiratory infections 14 300 (10 700–18 100)	Chronic obstructive pulmonary disease 10 500 (5 680–15 500)
90-94	Ischemic heart disease 8 640 (6 050–11 500)	Stroke 8 150 (5 830–10 900)	Lower respiratory infections 6 250 (5 110–7 560)	Alzheimer's disease and other dementias 5 870 (1 410–15 700)	Chronic obstructive pulmonary disease 5 490 (3 720–7 070)	Alzheimer's disease and other dementias 16 600 (4 510–41 400)	Stroke 16 100 (11 300–21 600)	Ischemic heart disease 14 800 (10 500–19 700)	Lower respiratory infections 10 400 (7 360–13 200)	Chronic obstructive pulmonary disease 6 700 (3 810–11 200)
95+	Ischemic heart disease 4 290 (2 790–5 860)	Alzheimer's disease and other dementias 3 880 (860–9 950)	Stroke 3 650 (2 520–5 150)	Lower respiratory infections 3 350 (2 560–4 210)	Chronic obstructive pulmonary disease 2 550 (1 740–3 400)	Alzheimer's disease and other dementias 13 200 (3 340–31 300)	Stroke 9 170 (5 610–13 800)	Ischemic heart disease 8 470 (5 190–11 800)	Lower respiratory infections 6 000 (4 130–8 080)	Chronic obstructive pulmonary disease 3 760 (2 160–6 460)

Table S5 | Mortality rates (per 100 000) for the top five Level 3 causes of death by sex and age groups in ASEAN, 2023

Age group (in years)	Males					Females				
	Top 1	Top 2	Top 3	Top 4	Top 5	Top 1	Top 2	Top 3	Top 4	Top 5
<1	Neonatal disorders 960·3 (801·4 - 1131·2)	Congenital birth defects 372·9 (245·5 - 533·3)	Lower respiratory infections 272·6 (172·0 - 407·9)	COVID-19 125·2 (102·7 - 153·8)	Diarrheal diseases 105·2 (43·1 - 200·6)	Neonatal disorders 684·6 (577·3 - 842·3)	Congenital birth defects 311·3 (208·6 - 444·8)	Lower respiratory infections 225·8 (146·9 - 310·1)	COVID-19 84·6 (67·1 - 107·2)	Diarrheal diseases 76·2 (33·4 - 148·2)
2-4	Drowning 17·7 (8·4 - 26·2)	Lower respiratory infections 12·0 (7·9 - 18·1)	Dengue 8·0 (1·9 - 20·3)	Congenital birth defects 7·7 (5·3 - 11·3)	Pertussis 5·5 (2·1 - 11·4)	Lower respiratory infections 12·8 (8·5 - 17·6)	Congenital birth defects 6·7 (4·3 - 10·1)	Pertussis 6·4 (2·6 - 12·1)	Drowning 5·9 (3·8 - 8·5)	Diarrheal diseases 4·6 (2·4 - 8·0)
5-9	Drowning 8·6 (5·2 - 11·5)	Lower respiratory infections 5·0 (3·5 - 7·0)	Road injuries 4·5 (2·5 - 6·3)	Congenital birth defects 3·0 (2·1 - 4·3)	Typhoid and paratyphoid 2·6 (1·2 - 4·6)	Lower respiratory infections 4·4 (2·9 - 6·6)	Drowning 4·1 (2·7 - 5·8)	Dengue 3·6 (1·1 - 8·9)	Congenital birth defects 2·8 (1·9 - 3·9)	Road injuries 2·8 (1·4 - 4·2)
10-14	Road injuries 7·7 (4·3 - 10·3)	Drowning 5·7 (4·1 - 7·5)	Lower respiratory infections 3·3 (2·5 - 4·5)	Congenital birth defects 2·8 (2·0 - 4·0)	Leukemia 2·4 (1·7 - 3·4)	Drowning 3·5 (2·4 - 4·6)	Road injuries 3·5 (1·9 - 4·8)	Lower respiratory infections 2·7 (2·0 - 3·9)	Congenital birth defects 2·5 (1·7 - 3·5)	Leukemia 2·0 (1·5 - 2·8)
15-19	Road injuries 28·9 (20·1 - 38·0)	Conflict and terrorism 12·5 (5·1 - 26·4)	Drowning 5·2 (3·5 - 7·4)	Self-harm 4·8 (3·0 - 6·8)	Interpersonal violence 4·6 (3·0 - 7·1)	Road injuries 11·2 (6·0 - 15·8)	Maternal disorders 4·9 (3·6 - 6·4)	Tuberculosis 4·6 (2·9 - 6·7)	Chronic kidney disease 3·3 (2·0 - 4·9)	Lower respiratory infections 3·1 (2·1 - 4·9)
20-24	Road injuries 37·0 (27·3 - 48·9)	Conflict and terrorism 9·5 (3·9 - 20·1)	Tuberculosis 7·7 (5·0 - 11·5)	Self-harm 7·6 (5·1 - 10·2)	Interpersonal violence 6·9 (5·1 - 9·4)	Road injuries 11·0 (6·6 - 15·1)	Maternal disorders 10·8 (8·2 - 13·8)	Tuberculosis 7·1 (4·6 - 10·3)	Chronic kidney disease 5·2 (3·2 - 7·5)	Ischemic heart disease 4·8 (2·8 - 7·2)
25-29	Road injuries 30·9 (23·5 - 41·0)	Tuberculosis 11·2 (7·8 - 16·4)	Ischemic heart disease 9·7 (7·0 - 13·2)	Cirrhosis and other chronic liver diseases 9·4 (6·5 - 12·9)	HIV/AIDS 9·3 (4·5 - 14·4)	Maternal disorders 11·1 (8·7 - 13·9)	Road injuries 9·1 (5·5 - 12·4)	Tuberculosis 8·0 (5·2 - 11·3)	Ischemic heart disease 6·4 (4·1 - 9·6)	HIV/AIDS 5·9 (3·1 - 9·2)
30-34	Road injuries 31·9 (22·7 - 45·1)	Ischemic heart disease 20·2 (14·7 - 27·3)	Cirrhosis and other chronic liver diseases 19·7 (14·1 - 27·5)	Stroke 14·9 (10·5 - 20·3)	Tuberculosis 14·4 (9·9 - 20·9)	Maternal disorders 12·7 (9·9 - 16·1)	Ischemic heart disease 12·4 (7·8 - 17·9)	Stroke 10·2 (6·5 - 15·5)	Road injuries 9·5 (6·1 - 13·3)	Tuberculosis 8·6 (5·7 - 11·7)

Age group (in years)	Males					Females				
	Top 1	Top 2	Top 3	Top 4	Top 5	Top 1	Top 2	Top 3	Top 4	Top 5
35-39	Ischemic heart disease 35·3 (26·2 - 45·9)	Cirrhosis and other chronic liver diseases 33·0 (25·1 - 42·4)	Road injuries 31·1 (23·3 - 42·1)	Stroke 30·6 (22·6 - 40·0)	Tuberculosis 17·3 (12·2 - 24·0)	Stroke 18·8 (12·1 - 27·5)	Ischemic heart disease 18·2 (11·7 - 26·7)	Breast cancer 16·0 (12·1 - 20·2)	Maternal disorders 10·4 (8·1 - 12·9)	Chronic kidney disease 10·0 (6·3 - 13·6)
40-44	Ischemic heart disease 60·2 (46·6 - 75·8)	Stroke 55·4 (43·3 - 70·8)	Cirrhosis and other chronic liver diseases 47·2 (35·6 - 61·3)	Road injuries 31·7 (21·8 - 43·9)	Tuberculosis 20·2 (13·7 - 27·8)	Stroke 41·5 (28·8 - 58·8)	Ischemic heart disease 36·4 (23·5 - 51·8)	Breast cancer 31·3 (24·0 - 41·2)	Chronic kidney disease 16·1 (11·2 - 22·1)	Cirrhosis and other chronic liver diseases 14·4 (10·0 - 20·9)
45-49	Ischemic heart disease 108·1 (85·3 - 141·7)	Stroke 104·8 (82·0 - 134·7)	Cirrhosis and other chronic liver diseases 66·0 (50·8 - 84·5)	Road injuries 35·9 (23·8 - 50·0)	Tuberculosis 28·3 (20·2 - 39·5)	Stroke 68·7 (47·4 - 93·8)	Ischemic heart disease 53·5 (36·1 - 72·1)	Breast cancer 42·4 (33·3 - 54·1)	Chronic kidney disease 20·1 (14·9 - 26·7)	Cirrhosis and other chronic liver diseases 18·1 (12·4 - 25·9)
50-54	Stroke 182·4 (143·1 - 227·5)	Ischemic heart disease 173·5 (135·5 - 216·1)	Cirrhosis and other chronic liver diseases 80·9 (63·1 - 101·7)	Diabetes mellitus 49·8 (34·8 - 70·5)	Tracheal, bronchus, and lung cancer 43·0 (32·6 - 55·6)	Stroke 105·2 (73·6 - 141·3)	Ischemic heart disease 77·0 (53·5 - 105·5)	Breast cancer 50·7 (39·1 - 63·2)	Diabetes mellitus 30·7 (20·9 - 42·8)	Chronic kidney disease 27·5 (20·8 - 37·0)
55-59	Stroke 260·8 (207·0 - 327·4)	Ischemic heart disease 244·4 (192·4 - 302·9)	Cirrhosis and other chronic liver diseases 88·9 (67·4 - 114·2)	Diabetes mellitus 74·6 (52·8 - 109·8)	Tracheal, bronchus, and lung cancer 65·8 (49·9 - 88·0)	Stroke 145·4 (104·7 - 188·4)	Ischemic heart disease 109·3 (78·7 - 148·3)	Breast cancer 56·3 (45·6 - 70·2)	Diabetes mellitus 52·3 (37·6 - 70·9)	Chronic kidney disease 38·5 (28·4 - 51·7)
60-64	Stroke 420·7 (332·1 - 529·3)	Ischemic heart disease 350·7 (274·4 - 435·2)	Diabetes mellitus 115·7 (82·5 - 159·7)	Tracheal, bronchus, and lung cancer 113·5 (84·1 - 151·3)	Cirrhosis and other chronic liver diseases 102·5 (75·9 - 134·4)	Stroke 235·1 (175·8 - 307·9)	Ischemic heart disease 166·3 (122·8 - 221·0)	Diabetes mellitus 89·6 (65·7 - 123·2)	Breast cancer 57·8 (46·8 - 71·9)	Chronic kidney disease 51·0 (38·0 - 71·4)
65-69	Stroke 561·7 (446·9 - 690·6)	Ischemic heart disease 447·7 (365·1 - 561·7)	Chronic obstructive pulmonary disease 170·6 (129·4 - 223·6)	Diabetes mellitus 170·6 (119·3 - 238·2)	Tracheal, bronchus, and lung cancer 148·7 (114·1 - 198·1)	Stroke 345·9 (265·7 - 433·6)	Ischemic heart disease 238·8 (185·8 - 299·4)	Diabetes mellitus 135·3 (99·8 - 186·0)	Chronic obstructive pulmonary disease 72·8 (37·5 - 115·8)	Chronic kidney disease 70·9 (53·7 - 98·0)
70-74	Stroke 871·7 (705·7 - 1075·2)	Ischemic heart disease	Chronic obstructive pulmonary disease	Diabetes mellitus 231·3 (165·0 - 325·1)	Lower respiratory infections	Stroke 571·1 (450·5 - 700·8)	Ischemic heart disease	Diabetes mellitus 198·3 (146·0 - 270·3)	Lower respiratory infections	Chronic obstructive pulmonary disease

Age group (in years)	Males					Females				
	Top 1	Top 2	Top 3	Top 4	Top 5	Top 1	Top 2	Top 3	Top 4	Top 5
		651·4<br>(538·6 - 798·9)	309·4<br>(226·5 - 404·1)		223·4<br>(179·8 - 277·3)		375·9<br>(293·2 - 477·4)		131·7<br>(98·7 - 169·4)	129·0<br>(72·8 - 202·0)
75-79	Stroke<br>1190·6<br>(974·2 - 1422·0)	Ischemic heart disease<br>854·1<br>(689·0 - 1051·6)	Chronic obstructive pulmonary disease<br>481·3<br>(370·3 - 609·8)	Lower respiratory infections<br>378·5<br>(322·9 - 448·4)	Diabetes mellitus<br>301·8<br>(222·0 - 409·0)	Stroke<br>808·2<br>(668·0 - 992·5)	Ischemic heart disease<br>544·2<br>(427·9 - 675·9)	Diabetes mellitus<br>263·1<br>(186·2 - 370·6)	Lower respiratory infections<br>241·9<br>(186·0 - 297·8)	Chronic obstructive pulmonary disease<br>211·1<br>(129·8 - 346·4)
80-84	Stroke<br>1675·0<br>(1328·4 - 2091·5)	Ischemic heart disease<br>1274·0<br>(990·8 - 1621·0)	Chronic obstructive pulmonary disease<br>781·3<br>(616·8 - 979·0)	Lower respiratory infections<br>699·8<br>(597·3 - 833·4)	Diabetes mellitus<br>368·5<br>(261·6 - 512·4)	Stroke<br>1297·2<br>(1045·4 - 1617·4)	Ischemic heart disease<br>948·7<br>(725·7 - 1183·6)	Alzheimer's disease and other dementias<br>492·5<br>(118·1 - 1255·9)	Lower respiratory infections<br>491·8<br>(396·6 - 612·5)	Chronic obstructive pulmonary disease<br>368·9<br>(207·2 - 595·7)
85-89	Stroke<br>1982·6<br>(1524·7 - 2461·9)	Ischemic heart disease<br>1773·5<br>(1392·0 - 2270·3)	Chronic obstructive pulmonary disease<br>1133·8<br>(866·6 - 1409·8)	Lower respiratory infections<br>1121·0<br>(953·0 - 1352·6)	Alzheimer's disease and other dementias<br>742·0<br>(169·9 - 2139·2)	Stroke<br>1759·9<br>(1290·7 - 2268·8)	Ischemic heart disease<br>1442·0<br>(1096·7 - 1847·8)	Alzheimer's disease and other dementias<br>1073·6<br>(283·2 - 2748·9)	Lower respiratory infections<br>853·8<br>(643·7 - 1082·9)	Chronic obstructive pulmonary disease<br>628·9<br>(340·1 - 930·8)
90-94	Ischemic heart disease<br>2225·6<br>(1558·2 - 2958·2)	Stroke<br>2098·9<br>(1502·1 - 2799·9)	Lower respiratory infections<br>1611·0<br>(1315·7 - 1946·4)	Alzheimer's disease and other dementias<br>1512·2<br>(363·5 - 4052·2)	Chronic obstructive pulmonary disease<br>1414·4<br>(958·7 - 1821·3)	Alzheimer's disease and other dementias<br>2149·1<br>(585·3 - 5368·4)	Stroke<br>2090·4<br>(1465·7 - 2798·9)	Ischemic heart disease<br>1923·9<br>(1364·3 - 2554·0)	Lower respiratory infections<br>1349·2<br>(955·7 - 1714·3)	Chronic obstructive pulmonary disease<br>869·3<br>(494·1 - 1454·3)
95+	Ischemic heart disease<br>3160·0<br>(2054·7 - 4319·2)	Alzheimer's disease and other dementias<br>2858·7<br>(633·6 - 7330·6)	Stroke<br>2687·4<br>(1859·7 - 3791·6)	Lower respiratory infections<br>2465·8<br>(1888·9 - 3105·5)	Chronic obstructive pulmonary disease<br>1880·1<br>(1282·0 - 2506·5)	Alzheimer's disease and other dementias<br>3909·7<br>(988·6 - 9260·0)	Stroke<br>2716·2<br>(1661·7 - 4088·7)	Ischemic heart disease<br>2508·2<br>(1535·7 - 3483·1)	Lower respiratory infections<br>1776·8<br>(1221·8 - 2391·5)	Chronic obstructive pulmonary disease<br>1112·6<br>(638·9 - 1913·9)

Table S6 | Mortality rates (per 100 000) for the top five Level 3 causes of death by sex and age groups in Brunei, 2023

Age group (in years)	Males					Females				
	Top 1	Top 2	Top 3	Top 4	Top 5	Top 1	Top 2	Top 3	Top 4	Top 5
<1	Neonatal disorders 332·3 (267·6 - 396·8)	Congenital birth defects 268·1 (212·2 - 332·0)	Endocrine, metabolic, blood, and immune disorders 29·0 (14·7 - 45·3)	Lower respiratory infections 21·8 (13·4 - 34·8)	Foreign body 10·5 (3·8 - 19·3)	Neonatal disorders 333·9 (273·4 - 417·3)	Congenital birth defects 328·4 (263·1 - 408·5)	Lower respiratory infections 24·4 (15·4 - 34·4)	Endocrine, metabolic, blood, and immune disorders 22·8 (12·9 - 35·9)	Foreign body 13·9 (6·1 - 23·8)
2-4	Congenital birth defects 5·1 (3·5 - 7·0)	Drowning 3·5 (2·2 - 5·5)	Road injuries 2·6 (1·7 - 4·0)	Endocrine, metabolic, blood, and immune disorders 2·4 (1·4 - 3·7)	Lower respiratory infections 1·7 (1·2 - 2·6)	Congenital birth defects 3·2 (2·1 - 4·6)	Road injuries 1·1 (0·7 - 1·7)	Lower respiratory infections 1·0 (0·6 - 1·4)	Eye cancer 0·9 (0·2 - 2·2)	Endocrine, metabolic, blood, and immune disorders 0·8 (0·5 - 1·4)
5-9	Drowning 2·6 (1·6 - 4·1)	Road injuries 1·9 (1·1 - 2·9)	Congenital birth defects 1·7 (1·1 - 2·5)	Brain and central nervous system cancer 1·3 (0·9 - 1·9)	Leukemia 1·2 (0·8 - 1·8)	Congenital birth defects 1·5 (1·1 - 2·1)	Road injuries 1·3 (0·8 - 2·1)	Brain and central nervous system cancer 0·9 (0·6 - 1·4)	Leukemia 0·9 (0·6 - 1·3)	Lower respiratory infections 0·8 (0·5 - 1·3)
10-14	Congenital birth defects 1·8 (1·2 - 2·6)	Leukemia 1·7 (1·1 - 2·4)	Brain and central nervous system cancer 1·5 (1·0 - 2·1)	Drowning 1·3 (0·8 - 2·0)	Lower respiratory infections 0·9 (0·6 - 1·3)	Congenital birth defects 1·7 (1·2 - 2·3)	Leukemia 1·5 (1·1 - 2·1)	Brain and central nervous system cancer 1·1 (0·8 - 1·8)	Lower respiratory infections 0·9 (0·5 - 1·4)	Road injuries 0·8 (0·5 - 1·4)
15-19	Road injuries 6·1 (3·6 - 9·6)	Self-harm 2·0 (1·2 - 3·0)	Drowning 1·8 (1·2 - 2·8)	Leukemia 1·7 (1·1 - 2·3)	Brain and central nervous system cancer 1·2 (0·8 - 1·7)	Road injuries 4·1 (2·4 - 7·3)	Leukemia 2·0 (1·4 - 2·9)	Congenital birth defects 1·4 (0·9 - 2·1)	Endocrine, metabolic, blood, and immune disorders 1·2 (0·7 - 1·9)	Diabetes mellitus 1·1 (0·6 - 1·8)
20-24	Road injuries 13·6 (8·6 - 20·0)	Self-harm 6·1 (3·6 - 8·9)	Drowning 3·1 (1·9 - 4·6)	Diabetes mellitus 2·3 (1·2 - 3·8)	Leukemia 2·2 (1·5 - 3·1)	Road injuries 3·9 (2·5 - 6·0)	Diabetes mellitus 1·9 (1·1 - 3·1)	Leukemia 1·7 (1·2 - 2·3)	Self-harm 1·7 (1·1 - 2·4)	Endocrine, metabolic, blood, and immune disorders 1·2 (0·7 - 1·8)
25-29	Road injuries 15·3 (10·5 - 22·6)	Self-harm 7·0 (4·4 - 9·9)	Ischemic heart disease 6·2 (3·8 - 9·2)	Drowning 3·9 (2·4 - 5·4)	Diabetes mellitus 3·4 (1·9 - 6·0)	Diabetes mellitus 2·4 (1·4 - 3·9)	Maternal disorders 2·3 (1·3 - 3·6)	Road injuries 1·8 (1·1 - 3·1)	Self-harm 1·7 (1·1 - 2·4)	Leukemia 1·5 (1·1 - 2·1)

Age group (in years)	Males					Females				
	Top 1	Top 2	Top 3	Top 4	Top 5	Top 1	Top 2	Top 3	Top 4	Top 5
30-34	Ischemic heart disease 14·9 (10·2 - 20·3)	Road injuries 14·4 (9·5 - 22·0)	Self-harm 6·8 (4·1 - 9·7)	Diabetes mellitus 6·0 (3·4 - 9·9)	Stroke 5·0 (2·9 - 8·1)	Breast cancer 6·2 (4·3 - 8·4)	Cervical cancer 3·6 (2·1 - 5·4)	Diabetes mellitus 3·2 (1·9 - 5·2)	Road injuries 2·9 (1·7 - 4·9)	Maternal disorders 2·7 (1·7 - 4·1)
35-39	Ischemic heart disease 27·7 (20·2 - 36·8)	Diabetes mellitus 13·7 (8·1 - 22·9)	Road injuries 11·6 (7·3 - 18·7)	Stroke 9·3 (6·0 - 13·6)	Self-harm 9·2 (5·6 - 12·7)	Breast cancer 14·9 (10·3 - 20·6)	Diabetes mellitus 7·8 (4·9 - 12·5)	Cervical cancer 7·3 (4·6 - 10·6)	Stroke 6·9 (4·3 - 10·4)	Ischemic heart disease 4·7 (2·7 - 7·3)
40-44	Ischemic heart disease 45·5 (34·2 - 58·8)	Diabetes mellitus 20·6 (11·9 - 35·2)	Stroke 17·7 (11·8 - 25·3)	Road injuries 11·8 (7·5 - 18·2)	Cirrhosis and other chronic liver diseases 9·9 (6·7 - 13·9)	Breast cancer 24·9 (18·7 - 33·2)	Stroke 13·6 (9·3 - 20·0)	Cervical cancer 11·0 (7·0 - 17·2)	Diabetes mellitus 9·8 (6·3 - 15·1)	Ischemic heart disease 7·7 (4·7 - 11·5)
45-49	Ischemic heart disease 112·4 (84·4 - 146·4)	Diabetes mellitus 46·3 (26·9 - 75·4)	Stroke 39·4 (26·1 - 56·1)	Cirrhosis and other chronic liver diseases 21·3 (14·7 - 29·4)	Colon and rectum cancer 20·8 (14·4 - 30·0)	Breast cancer 39·3 (29·2 - 52·5)	Stroke 24·2 (17·4 - 33·5)	Diabetes mellitus 17·7 (11·4 - 27·0)	Cervical cancer 15·0 (9·9 - 22·6)	Ischemic heart disease 14·8 (9·3 - 21·1)
50-54	Ischemic heart disease 151·5 (120·8 - 195·3)	Diabetes mellitus 69·2 (42·4 - 112·2)	Stroke 58·8 (39·8 - 80·1)	Colon and rectum cancer 36·0 (25·2 - 49·2)	Tracheal, bronchus, and lung cancer 30·4 (20·5 - 42·0)	Breast cancer 59·3 (43·5 - 77·6)	Stroke 40·1 (28·8 - 53·4)	Diabetes mellitus 34·3 (22·5 - 49·9)	Tracheal, bronchus, and lung cancer 26·0 (18·3 - 34·7)	Ischemic heart disease 25·0 (16·2 - 34·9)
55-59	Ischemic heart disease 168·6 (124·8 - 206·0)	Diabetes mellitus 105·2 (63·4 - 166·8)	Stroke 63·4 (44·6 - 86·4)	Tracheal, bronchus, and lung cancer 46·5 (33·8 - 62·8)	Colon and rectum cancer 46·3 (32·6 - 63·5)	Breast cancer 60·3 (42·4 - 78·6)	Diabetes mellitus 55·2 (36·3 - 80·3)	Stroke 47·2 (33·3 - 69·3)	Ischemic heart disease 38·8 (26·4 - 52·5)	Tracheal, bronchus, and lung cancer 37·0 (26·3 - 50·1)
60-64	Ischemic heart disease 188·4 (148·5 - 236·1)	Diabetes mellitus 160·2 (101·9 - 255·5)	Stroke 91·9 (65·9 - 126·6)	Tracheal, bronchus, and lung cancer 80·1 (57·6 - 116·3)	Colon and rectum cancer 68·6 (47·9 - 92·7)	Diabetes mellitus 112·2 (76·5 - 155·6)	Stroke 70·9 (52·0 - 98·8)	Ischemic heart disease 69·2 (48·6 - 91·0)	Tracheal, bronchus, and lung cancer 61·2 (42·4 - 81·5)	Breast cancer 59·5 (41·3 - 78·8)
65-69	Ischemic heart disease 244·8 (188·1 - 307·3)	Diabetes mellitus 213·5 (128·8 - 340·4)	Stroke 130·1 (95·1 - 180·4)	Tracheal, bronchus, and lung cancer 123·5 (90·6 - 172·0)	Colon and rectum cancer 89·4 (65·5 - 119·9)	Diabetes mellitus 146·6 (95·3 - 212·7)	Stroke 105·0 (73·5 - 150·6)	Ischemic heart disease 101·9 (72·0 - 132·1)	Tracheal, bronchus, and lung cancer 84·1 (61·1 - 111·9)	Chronic kidney disease 67·9 (46·7 - 95·6)

Age group (in years)	Males					Females				
	Top 1	Top 2	Top 3	Top 4	Top 5	Top 1	Top 2	Top 3	Top 4	Top 5
70-74	Ischemic heart disease 336·7 (253·5 - 434·3)	Diabetes mellitus 278·8 (170·2 - 443·5)	Stroke 212·3 (155·7 - 277·2)	Tracheal, bronchus, and lung cancer 181·8 (137·3 - 240·9)	Lower respiratory infections 138·7 (95·5 - 209·3)	Diabetes mellitus 286·5 (193·4 - 425·4)	Stroke 212·7 (159·5 - 299·9)	Ischemic heart disease 206·0 (154·2 - 281·3)	Chronic obstructive pulmonary disease 157·8 (97·1 - 284·9)	Tracheal, bronchus, and lung cancer 142·5 (98·1 - 187·9)
75-79	Ischemic heart disease 744·2 (569·6 - 977·7)	Stroke 504·3 (360·8 - 667·2)	Diabetes mellitus 487·2 (288·0 - 806·4)	Tracheal, bronchus, and lung cancer 337·2 (243·6 - 459·3)	Lower respiratory infections 335·9 (225·4 - 458·0)	Stroke 507·2 (380·7 - 670·5)	Diabetes mellitus 457·6 (301·3 - 668·1)	Ischemic heart disease 451·8 (332·1 - 585·8)	Chronic obstructive pulmonary disease 301·2 (191·5 - 531·5)	Chronic kidney disease 277·3 (188·7 - 379·8)
80-84	Ischemic heart disease 1101·2 (840·3 - 1421·7)	Stroke 796·0 (603·0 - 1046·5)	Lower respiratory infections 674·2 (450·5 - 938·2)	Diabetes mellitus 642·8 (376·9 - 1020·8)	Chronic obstructive pulmonary disease 584·8 (392·0 - 799·5)	Stroke 812·2 (581·6 - 1093·0)	Ischemic heart disease 739·6 (524·2 - 947·3)	Diabetes mellitus 658·6 (415·4 - 996·4)	Chronic obstructive pulmonary disease 516·5 (310·0 - 1013·9)	Alzheimer's disease and other dementias 485·5 (118·0 - 1244·1)
85-89	Ischemic heart disease 1518·6 (1143·2 - 1978·0)	Lower respiratory infections 1215·4 (862·7 - 1689·2)	Stroke 1080·8 (801·6 - 1489·9)	Chronic obstructive pulmonary disease 927·5 (648·5 - 1309·8)	Diabetes mellitus 896·6 (473·2 - 1541·6)	Stroke 1371·1 (970·0 - 1884·2)	Ischemic heart disease 1193·7 (781·1 - 1625·1)	Alzheimer's disease and other dementias 1151·5 (280·3 - 2760·4)	Diabetes mellitus 952·1 (578·8 - 1429·5)	Chronic obstructive pulmonary disease 907·6 (518·9 - 1747·5)
90-94	Ischemic heart disease 2832·0 (2008·2 - 3867·1)	Lower respiratory infections 2430·0 (1608·2 - 3538·5)	Stroke 1913·5 (1303·3 - 2653·4)	Chronic obstructive pulmonary disease 1454·1 (978·7 - 2007·1)	Alzheimer's disease and other dementias 1427·3 (327·1 - 4208·7)	Alzheimer's disease and other dementias 2554·0 (668·9 - 6802·9)	Stroke 2236·6 (1504·4 - 3368·2)	Ischemic heart disease 2144·8 (1409·0 - 2973·6)	Chronic obstructive pulmonary disease 1484·1 (797·0 - 3219·7)	Diabetes mellitus 1427·7 (831·7 - 2244·0)
95+	Ischemic heart disease 3934·7 (2489·2 - 5442·0)	Lower respiratory infections 3876·8 (2533·8 - 5693·4)	Stroke 2498·0 (1542·4 - 3757·4)	Alzheimer's disease and other dementias 2261·0 (492·1 - 6111·4)	Chronic kidney disease 1897·3 (1128·4 - 2907·3)	Alzheimer's disease and other dementias 4862·9 (1224·1 - 11759·0)	Stroke 3356·1 (1938·3 - 5088·9)	Ischemic heart disease 3207·7 (1867·8 - 4545·3)	Chronic obstructive pulmonary disease 2311·2 (1182·6 - 4884·7)	Diabetes mellitus 2119·7 (1171·8 - 3187·8)

Table S7 | Mortality rates (per 100 000) for the top five Level 3 causes of death by sex and age groups in Cambodia, 2023

Age group (in years)	Males					Females				
	Top 1	Top 2	Top 3	Top 4	Top 5	Top 1	Top 2	Top 3	Top 4	Top 5
<1	Neonatal disorders 840·2 (611·3 - 1096·5)	Lower respiratory infections 493·5 (283·6 - 804·0)	Congenital birth defects 314·0 (179·2 - 525·1)	COVID-19 83·1 (53·5 - 130·0)	Diarrheal diseases 74·9 (25·3 - 164·3)	Neonatal disorders 524·7 (386·8 - 736·3)	Lower respiratory infections 334·3 (207·7 - 482·3)	Congenital birth defects 283·2 (157·8 - 432·5)	Other unspecified infectious diseases 59·6 (19·2 - 136·8)	COVID-19 49·0 (32·8 - 71·7)
2-4	Lower respiratory infections 18·3 (9·4 - 31·7)	Drowning 16·0 (7·9 - 29·0)	Congenital birth defects 7·9 (4·4 - 13·8)	Road injuries 5·4 (2·5 - 9·5)	Pertussis 4·1 (0·4 - 11·2)	Lower respiratory infections 25·0 (14·4 - 37·7)	Drowning 10·3 (4·8 - 19·7)	Congenital birth defects 9·4 (4·8 - 16·7)	Pertussis 6·3 (0·6 - 18·0)	Road injuries 4·3 (1·8 - 8·2)
5-9	Drowning 14·5 (7·3 - 25·1)	Lower respiratory infections 8·9 (4·7 - 13·9)	Road injuries 7·0 (3·0 - 12·5)	Congenital birth defects 3·6 (2·0 - 5·9)	Falls 2·4 (1·2 - 3·9)	Drowning 12·0 (5·9 - 21·1)	Lower respiratory infections 11·7 (5·6 - 20·9)	Road injuries 6·8 (3·1 - 11·6)	Congenital birth defects 3·9 (2·2 - 6·5)	Pertussis 2·2 (0·2 - 6·5)
10-14	Drowning 10·1 (5·8 - 17·3)	Road injuries 8·2 (4·5 - 12·9)	Lower respiratory infections 5·8 (3·4 - 9·6)	Congenital birth defects 3·1 (1·8 - 5·3)	Leukemia 2·3 (1·4 - 3·6)	Drowning 7·8 (3·8 - 13·7)	Lower respiratory infections 6·2 (3·4 - 10·1)	Road injuries 4·9 (2·2 - 8·9)	Congenital birth defects 3·5 (2·0 - 5·7)	Rheumatic heart disease 2·5 (1·2 - 4·4)
15-19	Road injuries 38·8 (22·8 - 63·0)	Drowning 13·2 (6·6 - 23·5)	Lower respiratory infections 6·7 (4·1 - 11·2)	Interpersonal violence 6·0 (2·4 - 12·3)	Falls 5·4 (2·8 - 8·8)	Road injuries 8·1 (3·7 - 14·2)	Lower respiratory infections 5·7 (3·1 - 10·1)	Tuberculosis 4·7 (2·0 - 8·4)	Congenital birth defects 3·0 (1·7 - 4·8)	Drowning 2·8 (1·3 - 5·7)
20-24	Road injuries 49·6 (27·7 - 75·2)	Drowning 10·8 (5·6 - 18·5)	Interpersonal violence 9·2 (4·2 - 19·2)	Tuberculosis 8·4 (4·7 - 15·1)	Lower respiratory infections 7·8 (4·6 - 12·8)	Tuberculosis 8·0 (3·0 - 14·3)	Road injuries 7·9 (4·0 - 14·5)	HIV/AIDS 6·0 (1·9 - 12·6)	Lower respiratory infections 6·0 (3·1 - 10·3)	Other unspecified infectious diseases 3·1 (1·3 - 6·4)
25-29	Road injuries 33·9 (18·3 - 58·3)	Tuberculosis 12·2 (6·7 - 21·0)	Ischemic heart disease 11·3 (6·4 - 17·8)	Interpersonal violence 9·0 (4·2 - 19·0)	Lower respiratory infections 8·7 (5·0 - 13·7)	HIV/AIDS 11·2 (2·4 - 25·2)	Tuberculosis 9·9 (4·1 - 19·5)	Lower respiratory infections 6·7 (3·5 - 11·4)	Road injuries 6·1 (3·0 - 11·0)	Rheumatic heart disease 3·4 (1·2 - 7·4)
30-34	Road injuries 34·7 (19·2 - 54·4)	Ischemic heart disease 23·5 (14·3 - 35·9)	Tuberculosis 16·4 (8·6 - 27·9)	Cirrhosis and other chronic liver diseases 14·7 (8·2 - 23·7)	Lower respiratory infections 13·6 (7·9 - 22·5)	HIV/AIDS 16·2 (3·4 - 32·7)	Tuberculosis 10·9 (4·5 - 22·0)	Lower respiratory infections 7·6 (4·2 - 14·2)	Stroke 5·3 (2·7 - 8·9)	Ischemic heart disease 5·1 (2·5 - 9·1)

Age group (in years)	Males					Females				
	Top 1	Top 2	Top 3	Top 4	Top 5	Top 1	Top 2	Top 3	Top 4	Top 5
35-39	Ischemic heart disease 62·8 (37·7 - 96·8)	Road injuries 52·2 (29·6 - 83·9)	Stroke 35·3 (20·9 - 58·1)	Cirrhosis and other chronic liver diseases 29·7 (17·9 - 44·7)	Tuberculosis 24·6 (13·7 - 42·2)	HIV/AIDS 20·3 (3·1 - 42·2)	Tuberculosis 12·6 (5·3 - 27·0)	Lower respiratory infections 8·9 (4·9 - 14·8)	Stroke 8·7 (4·8 - 14·8)	Ischemic heart disease 7·1 (3·4 - 11·9)
40-44	Ischemic heart disease 95·2 (56·2 - 142·4)	Stroke 58·7 (34·3 - 92·0)	Cirrhosis and other chronic liver diseases 48·8 (27·6 - 76·1)	Road injuries 47·2 (27·4 - 72·6)	Tuberculosis 32·9 (17·4 - 62·0)	HIV/AIDS 27·7 (3·5 - 55·0)	Stroke 21·4 (11·6 - 34·9)	Tuberculosis 17·4 (7·0 - 34·8)	Ischemic heart disease 16·8 (8·6 - 27·7)	Breast cancer 16·5 (10·5 - 25·4)
45-49	Ischemic heart disease 112·5 (67·8 - 156·7)	Stroke 79·2 (45·5 - 118·4)	Cirrhosis and other chronic liver diseases 64·7 (40·1 - 104·2)	Tuberculosis 43·3 (23·5 - 79·6)	Road injuries 37·0 (20·7 - 61·9)	Stroke 74·2 (46·0 - 117·8)	Ischemic heart disease 52·4 (29·0 - 85·1)	Breast cancer 46·9 (30·0 - 72·1)	HIV/AIDS 37·9 (7·9 - 87·2)	Tuberculosis 26·7 (11·1 - 55·3)
50-54	Ischemic heart disease 160·4 (104·5 - 230·9)	Stroke 123·0 (75·0 - 185·5)	Cirrhosis and other chronic liver diseases 84·4 (50·3 - 136·4)	Tuberculosis 60·1 (32·2 - 113·0)	Lower respiratory infections 37·9 (21·5 - 64·7)	Stroke 124·0 (77·3 - 186·9)	Ischemic heart disease 85·1 (45·3 - 131·8)	Breast cancer 62·0 (39·8 - 91·3)	Tuberculosis 37·0 (13·7 - 83·9)	Diabetes mellitus 36·7 (19·7 - 57·4)
55-59	Ischemic heart disease 223·3 (143·1 - 322·2)	Stroke 174·0 (98·7 - 258·8)	Cirrhosis and other chronic liver diseases 99·1 (56·4 - 152·8)	Tuberculosis 80·4 (42·2 - 160·4)	Chronic obstructive pulmonary disease 64·3 (37·5 - 105·1)	Stroke 158·9 (99·3 - 237·0)	Ischemic heart disease 112·1 (58·1 - 179·5)	Diabetes mellitus 62·8 (35·5 - 100·1)	Breast cancer 61·8 (37·6 - 97·1)	Lower respiratory infections 47·5 (24·4 - 85·9)
60-64	Ischemic heart disease 281·4 (181·8 - 403·4)	Stroke 244·3 (149·2 - 353·5)	Chronic obstructive pulmonary disease 136·7 (84·6 - 221·4)	Tuberculosis 116·2 (64·5 - 206·1)	Cirrhosis and other chronic liver diseases 109·5 (59·3 - 182·5)	Stroke 219·8 (146·2 - 324·5)	Ischemic heart disease 155·7 (84·6 - 241·8)	Diabetes mellitus 100·6 (57·4 - 167·2)	Lower respiratory infections 67·9 (33·2 - 126·6)	Tuberculosis 65·3 (28·3 - 134·3)
65-69	Ischemic heart disease 398·0 (245·0 - 596·3)	Stroke 377·5 (241·0 - 520·5)	Chronic obstructive pulmonary disease 262·9 (152·2 - 432·0)	Tuberculosis 160·6 (89·0 - 299·9)	Lower respiratory infections 138·5 (80·5 - 233·9)	Stroke 340·7 (207·0 - 508·6)	Ischemic heart disease 228·6 (124·2 - 368·4)	Diabetes mellitus 148·8 (83·0 - 244·7)	Lower respiratory infections 101·6 (47·5 - 185·9)	Tuberculosis 87·4 (38·0 - 166·7)

Age group (in years)	Males					Females				
	Top 1	Top 2	Top 3	Top 4	Top 5	Top 1	Top 2	Top 3	Top 4	Top 5
70-74	Stroke 630·5 (388·5 - 915·2)	Ischemic heart disease 623·3 (409·9 - 907·1)	Chronic obstructive pulmonary disease 507·1 (292·5 - 795·8)	Lower respiratory infections 235·2 (127·6 - 391·3)	Tuberculosis 220·0 (120·3 - 395·1)	Stroke 576·2 (385·1 - 808·0)	Ischemic heart disease 356·8 (200·8 - 567·4)	Lower respiratory infections 214·6 (109·0 - 386·6)	Diabetes mellitus 211·7 (117·1 - 350·9)	Chronic obstructive pulmonary disease 134·3 (68·4 - 234·6)
75-79	Stroke 1029·6 (630·7 - 1564·7)	Ischemic heart disease 1011·2 (610·9 - 1545·5)	Chronic obstructive pulmonary disease 853·6 (504·4 - 1377·6)	Lower respiratory infections 408·4 (232·0 - 662·1)	Tuberculosis 296·7 (165·8 - 503·1)	Stroke 1006·4 (655·8 - 1423·6)	Ischemic heart disease 611·7 (325·9 - 985·9)	Lower respiratory infections 433·4 (217·5 - 752·0)	Diabetes mellitus 291·7 (157·8 - 486·1)	Chronic obstructive pulmonary disease 236·9 (126·5 - 426·5)
80-84	Ischemic heart disease 1704·7 (1031·3 - 2641·4)	Stroke 1562·1 (941·2 - 2314·4)	Chronic obstructive pulmonary disease 1346·4 (824·3 - 2172·4)	Lower respiratory infections 802·9 (459·0 - 1274·7)	Tuberculosis 368·3 (201·6 - 620·7)	Stroke 1861·6 (1177·3 - 2717·1)	Ischemic heart disease 1256·6 (698·6 - 2045·6)	Lower respiratory infections 1001·8 (510·3 - 1700·9)	Alzheimer's disease and other dementias 471·5 (107·5 - 1466·3)	Chronic obstructive pulmonary disease 391·6 (201·4 - 720·8)
85-89	Ischemic heart disease 2456·4 (1460·0 - 3866·6)	Stroke 2056·1 (1330·8 - 3102·4)	Chronic obstructive pulmonary disease 1987·9 (1184·1 - 3228·6)	Lower respiratory infections 1235·1 (708·4 - 1986·2)	Alzheimer's disease and other dementias 679·4 (149·3 - 2017·0)	Stroke 3020·1 (1849·4 - 4545·1)	Ischemic heart disease 2418·6 (1359·8 - 4033·4)	Lower respiratory infections 1864·3 (961·2 - 3190·6)	Alzheimer's disease and other dementias 1055·0 (246·0 - 3216·8)	Chronic obstructive pulmonary disease 664·6 (338·9 - 1195·3)
90-94	Ischemic heart disease 3153·2 (1879·1 - 4660·8)	Chronic obstructive pulmonary disease 2382·7 (1448·2 - 3826·6)	Stroke 2171·9 (1302·6 - 3515·1)	Lower respiratory infections 1888·0 (1145·2 - 3083·7)	Alzheimer's disease and other dementias 1370·9 (283·7 - 3752·5)	Ischemic heart disease 3952·6 (2162·6 - 6513·5)	Stroke 3838·9 (2263·7 - 6361·2)	Lower respiratory infections 3298·1 (1655·1 - 5733·4)	Alzheimer's disease and other dementias 2002·7 (454·9 - 5615·9)	Chronic obstructive pulmonary disease 931·8 (458·0 - 1645·9)
95+	Ischemic heart disease 4017·8 (2214·2 - 6299·1)	Lower respiratory infections 2964·3 (1629·0 - 4871·2)	Chronic obstructive pulmonary disease 2760·9 (1651·1 - 4281·4)	Stroke 2457·4 (1234·3 - 4285·6)	Alzheimer's disease and other dementias 2382·3 (500·5 - 6371·6)	Ischemic heart disease 6507·1 (3344·1 - 9851·9)	Lower respiratory infections 5297·7 (2651·5 - 8944·6)	Stroke 4722·3 (2581·9 - 8514·3)	Alzheimer's disease and other dementias 3238·9 (636·2 - 9032·9)	Hypertensive heart disease 1126·9 (409·6 - 2475·3)

Table S8 | Mortality rates (per 100 000) for the top five Level 3 causes of death by sex and age groups in Indonesia, 2023

Age group (in years)	Males Top 1	Males Top 2	Males Top 3	Males Top 4	Males Top 5	Females Top 1	Females Top 2	Females Top 3	Females Top 4	Females Top 5
<1	Neonatal disorders<br>955·4<br>(688·5 - 1235·3)	Congenital birth defects<br>426·2<br>(248·7 - 657·7)	Lower respiratory infections<br>229·5<br>(124·0 - 380·7)	COVID-19<br>169·1<br>(122·5 - 229·7)	Diarrheal diseases<br>137·8<br>(47·4 - 284·8)	Neonatal disorders<br>651·1<br>(495·9 - 880·8)	Congenital birth defects<br>319·3<br>(190·5 - 477·1)	Lower respiratory infections<br>213·9<br>(116·6 - 320·0)	Diarrheal diseases<br>123·1<br>(47·8 - 257·9)	COVID-19<br>117·0<br>(79·6 - 158·5)
2-4	Dengue<br>17·9<br>(3·9 - 45·8)	Lower respiratory infections<br>11·3<br>(5·7 - 18·5)	Drowning<br>9·5<br>(3·1 - 17·9)	Congenital birth defects<br>6·5<br>(3·9 - 10·3)	Diarrheal diseases<br>6·5<br>(2·3 - 13·6)	Lower respiratory infections<br>10·9<br>(6·1 - 16·8)	Dengue<br>9·3<br>(2·6 - 24·8)	Pertussis<br>7·1<br>(0·8 - 19·9)	Diarrheal diseases<br>5·6<br>(2·3 - 11·3)	Drowning<br>4·8<br>(2·5 - 8·3)
5-9	Road injuries<br>5·4<br>(2·3 - 8·6)	Dengue<br>4·7<br>(0·8 - 14·9)	Drowning<br>4·6<br>(2·2 - 8·3)	Lower respiratory infections<br>3·6<br>(1·9 - 5·9)	Typhoid and paratyphoid<br>3·2<br>(1·4 - 6·2)	Dengue<br>7·8<br>(2·1 - 18·8)	Lower respiratory infections<br>3·6<br>(1·8 - 6·8)	Road injuries<br>3·3<br>(1·4 - 5·9)	Typhoid and paratyphoid<br>2·8<br>(1·0 - 5·5)	Drowning<br>2·6<br>(1·3 - 4·8)
10-14	Road injuries<br>10·0<br>(5·1 - 15·1)	Drowning<br>3·3<br>(1·9 - 5·6)	COVID-19<br>3·1<br>(2·1 - 4·6)	Leukemia<br>2·8<br>(1·7 - 4·3)	Congenital birth defects<br>2·7<br>(1·6 - 4·5)	Road injuries<br>4·2<br>(1·8 - 6·8)	Cirrhosis and other chronic liver diseases<br>2·5<br>(1·3 - 4·0)	Leukemia<br>2·4<br>(1·5 - 3·7)	Lower respiratory infections<br>2·2<br>(1·2 - 3·7)	Tuberculosis<br>2·2<br>(1·2 - 3·5)
15-19	Road injuries<br>35·2<br>(20·6 - 51·8)	Cirrhosis and other chronic liver diseases<br>6·2<br>(3·6 - 10·3)	Tuberculosis<br>5·3<br>(2·9 - 9·0)	Drowning<br>3·4<br>(1·7 - 5·7)	Interpersonal violence<br>3·4<br>(1·4 - 7·8)	Road injuries<br>14·7<br>(7·3 - 23·0)	Tuberculosis<br>7·3<br>(4·1 - 11·9)	Maternal disorders<br>6·9<br>(4·5 - 9·7)	Cirrhosis and other chronic liver diseases<br>6·3<br>(3·5 - 9·6)	Dengue<br>4·3<br>(0·9 - 13·4)
20-24	Road injuries<br>38·5<br>(24·7 - 63·5)	Tuberculosis<br>10·8<br>(6·3 - 17·0)	Cirrhosis and other chronic liver diseases<br>10·0<br>(5·9 - 16·0)	Ischemic heart disease<br>4·8<br>(2·6 - 7·8)	COVID-19<br>4·6<br>(3·2 - 6·6)	Maternal disorders<br>13·3<br>(9·4 - 18·0)	Road injuries<br>11·8<br>(6·8 - 18·4)	Tuberculosis<br>10·6<br>(6·4 - 17·3)	Cirrhosis and other chronic liver diseases<br>8·9<br>(5·8 - 13·7)	COVID-19<br>6·1<br>(4·5 - 8·2)
25-29	Road injuries<br>26·7<br>(16·8 - 44·9)	Tuberculosis<br>14·0<br>(8·2 - 21·6)	Cirrhosis and other chronic liver diseases<br>13·4<br>(8·3 - 20·2)	Ischemic heart disease<br>8·9<br>(4·9 - 13·9)	COVID-19<br>5·9<br>(4·0 - 8·2)	Maternal disorders<br>14·1<br>(9·8 - 18·9)	Tuberculosis<br>11·4<br>(6·7 - 17·7)	Cirrhosis and other chronic liver diseases<br>10·0<br>(6·0 - 14·6)	Road injuries<br>8·8<br>(5·5 - 13·6)	Ischemic heart disease<br>8·1<br>(4·6 - 13·5)
30-34	Road injuries<br>26·2<br>(16·3 - 44·2)	Cirrhosis and other chronic liver diseases<br>22·5<br>(14·4 - 35·7)	Ischemic heart disease<br>20·8<br>(12·8 - 31·6)	Tuberculosis<br>16·6<br>(10·2 - 25·4)	Stroke<br>13·8<br>(8·1 - 21·0)	Ischemic heart disease<br>16·5<br>(9·8 - 25·9)	Maternal disorders<br>16·0<br>(10·8 - 23·0)	Cirrhosis and other chronic liver diseases<br>15·2<br>(9·7 - 22·1)	Stroke<br>14·8<br>(8·5 - 23·7)	Tuberculosis<br>12·0<br>(6·8 - 18·1)

Age group (in years)	Males					Females				
	Top 1	Top 2	Top 3	Top 4	Top 5	Top 1	Top 2	Top 3	Top 4	Top 5
35-39	Ischemic heart disease 34·2 (21·0 - 50·3)	Cirrhosis and other chronic liver diseases 29·5 (19·9 - 42·8)	Stroke 27·2 (16·9 - 40·1)	Road injuries 22·7 (13·8 - 40·2)	Tuberculosis 18·4 (11·6 - 27·2)	Stroke 28·2 (15·9 - 44·8)	Ischemic heart disease 25·7 (14·6 - 41·0)	Cirrhosis and other chronic liver diseases 17·5 (11·8 - 25·8)	Breast cancer 16·4 (10·7 - 24·2)	Maternal disorders 13·2 (8·8 - 18·7)
40-44	Ischemic heart disease 61·1 (38·0 - 92·2)	Stroke 50·4 (32·8 - 77·8)	Cirrhosis and other chronic liver diseases 39·7 (24·8 - 60·5)	Road injuries 23·0 (14·0 - 42·9)	Tuberculosis 22·3 (13·0 - 32·8)	Stroke 66·2 (41·5 - 100·9)	Ischemic heart disease 54·9 (32·8 - 84·1)	Breast cancer 37·3 (23·7 - 55·9)	Cirrhosis and other chronic liver diseases 26·2 (17·3 - 39·0)	Chronic kidney disease 20·6 (12·6 - 31·1)
45-49	Ischemic heart disease 129·6 (87·0 - 199·6)	Stroke 116·3 (78·2 - 170·4)	Cirrhosis and other chronic liver diseases 63·4 (40·7 - 95·0)	Tuberculosis 34·5 (22·3 - 51·4)	Road injuries 30·5 (17·9 - 58·1)	Stroke 104·2 (64·3 - 153·7)	Ischemic heart disease 75·5 (45·5 - 110·1)	Breast cancer 46·2 (29·4 - 70·1)	Cirrhosis and other chronic liver diseases 30·4 (18·7 - 45·7)	Chronic kidney disease 23·1 (15·0 - 34·1)
50-54	Ischemic heart disease 212·4 (141·4 - 302·7)	Stroke 207·9 (142·6 - 285·9)	Cirrhosis and other chronic liver diseases 82·4 (51·6 - 119·1)	Diabetes mellitus 57·2 (30·9 - 92·5)	Tuberculosis 49·3 (31·3 - 71·7)	Stroke 156·8 (92·3 - 229·8)	Ischemic heart disease 106·5 (63·2 - 160·6)	Breast cancer 52·5 (33·1 - 75·3)	Cirrhosis and other chronic liver diseases 37·0 (23·7 - 57·8)	Diabetes mellitus 32·6 (18·6 - 52·9)
55-59	Ischemic heart disease 302·2 (190·4 - 415·8)	Stroke 301·6 (206·8 - 417·2)	Cirrhosis and other chronic liver diseases 98·6 (59·9 - 152·2)	Diabetes mellitus 89·3 (46·1 - 153·9)	Tracheal, bronchus, and lung cancer 68·8 (41·9 - 105·2)	Stroke 213·1 (130·6 - 307·3)	Ischemic heart disease 144·2 (87·9 - 221·0)	Diabetes mellitus 54·7 (29·7 - 87·0)	Breast cancer 53·6 (35·0 - 76·6)	Cirrhosis and other chronic liver diseases 50·8 (30·7 - 81·3)
60-64	Stroke 496·1 (330·3 - 724·5)	Ischemic heart disease 437·8 (288·3 - 606·0)	Diabetes mellitus 141·5 (79·1 - 228·5)	Cirrhosis and other chronic liver diseases 131·9 (87·4 - 193·8)	Chronic obstructive pulmonary disease 112·7 (69·4 - 168·3)	Stroke 351·9 (218·3 - 502·1)	Ischemic heart disease 219·0 (137·7 - 322·6)	Diabetes mellitus 93·8 (53·2 - 160·0)	Cirrhosis and other chronic liver diseases 77·6 (48·4 - 117·3)	Breast cancer 56·0 (33·6 - 85·0)
65-69	Stroke 629·9 (441·6 - 894·8)	Ischemic heart disease 513·1 (346·6 - 752·5)	Diabetes mellitus 207·3 (119·2 - 346·9)	Chronic obstructive pulmonary disease 184·6 (113·8 - 288·2)	Cirrhosis and other chronic liver diseases 153·6 (93·2 - 219·3)	Stroke 487·0 (330·7 - 693·3)	Ischemic heart disease 285·1 (180·8 - 425·3)	Diabetes mellitus 134·3 (68·8 - 222·4)	Cirrhosis and other chronic liver diseases 91·7 (57·1 - 140·4)	Chronic obstructive pulmonary disease 77·9 (38·6 - 138·8)

Age group (in years)	Males					Females				
	Top 1	Top 2	Top 3	Top 4	Top 5	Top 1	Top 2	Top 3	Top 4	Top 5
70-74	Stroke 952·3 (680·0 - 1331·2)	Ischemic heart disease 734·0 (500·3 - 1032·6)	Chronic obstructive pulmonary disease 322·2 (195·6 - 505·9)	Diabetes mellitus 286·3 (160·1 - 474·5)	COVID-19 198·5 (144·0 - 259·8)	Stroke 741·7 (501·9 - 1037·8)	Ischemic heart disease 414·3 (272·3 - 596·4)	Diabetes mellitus 195·3 (100·5 - 322·4)	Chronic obstructive pulmonary disease 126·1 (62·0 - 223·3)	Cirrhosis and other chronic liver diseases 125·9 (78·3 - 190·3)
75-79	Stroke 1132·5 (772·3 - 1574·3)	Ischemic heart disease 883·9 (559·0 - 1248·8)	Chronic obstructive pulmonary disease 413·1 (259·2 - 631·5)	Diabetes mellitus 376·0 (202·1 - 619·2)	COVID-19 280·0 (211·7 - 372·4)	Stroke 903·0 (616·4 - 1267·0)	Ischemic heart disease 492·8 (320·9 - 746·1)	Diabetes mellitus 259·8 (139·0 - 438·1)	Chronic obstructive pulmonary disease 188·0 (89·3 - 407·1)	Diarrheal diseases 178·0 (75·2 - 313·0)
80-84	Stroke 1635·3 (1089·1 - 2365·9)	Ischemic heart disease 1286·6 (758·9 - 1934·0)	Chronic obstructive pulmonary disease 630·2 (391·0 - 950·5)	COVID-19 531·8 (394·9 - 719·3)	Diabetes mellitus 471·5 (243·8 - 799·2)	Stroke 1442·3 (1030·8 - 1970·4)	Ischemic heart disease 797·6 (487·1 - 1193·1)	Alzheimer's disease and other dementias 430·7 (89·7 - 1190·7)	Diabetes mellitus 350·3 (187·8 - 589·5)	Diarrheal diseases 329·9 (127·1 - 592·3)
85-89	Stroke 1986·3 (1324·4 - 2983·6)	Ischemic heart disease 1712·1 (1068·7 - 2667·9)	Chronic obstructive pulmonary disease 877·3 (512·3 - 1294·8)	COVID-19 771·0 (538·8 - 1026·9)	Alzheimer's disease and other dementias 691·5 (143·8 - 2015·1)	Stroke 1843·4 (1185·9 - 2686·9)	Ischemic heart disease 1006·9 (600·9 - 1630·5)	Alzheimer's disease and other dementias 944·3 (208·4 - 2606·6)	Diarrheal diseases 609·7 (226·0 - 1130·6)	Chronic obstructive pulmonary disease 424·8 (195·6 - 1007·8)
90-94	Ischemic heart disease 2766·9 (1744·9 - 4017·4)	Stroke 2582·9 (1600·5 - 3942·7)	COVID-19 1596·3 (1095·2 - 2199·6)	Alzheimer's disease and other dementias 1465·9 (312·9 - 4008·5)	Chronic obstructive pulmonary disease 1164·3 (675·9 - 1658·6)	Stroke 2446·4 (1553·7 - 3728·5)	Alzheimer's disease and other dementias 1863·9 (429·0 - 4740·7)	Ischemic heart disease 1550·3 (811·9 - 2522·5)	Diarrheal diseases 1040·6 (357·0 - 1842·6)	Chronic obstructive pulmonary disease 580·5 (243·4 - 1512·9)
95+	Ischemic heart disease 4958·0 (2822·2 - 7661·2)	Stroke 3999·5 (2241·8 - 6580·5)	Alzheimer's disease and other dementias 2937·0 (651·6 - 8118·7)	COVID-19 2363·5 (1642·1 - 3270·0)	Chronic obstructive pulmonary disease 1679·5 (951·2 - 2674·1)	Stroke 3540·6 (1927·0 - 5869·9)	Alzheimer's disease and other dementias 3425·2 (866·4 - 8923·3)	Ischemic heart disease 2618·7 (1345·4 - 4385·1)	Diarrheal diseases 1757·2 (621·5 - 3177·6)	Protein-energy malnutrition 1072·7 (637·5 - 1563·1)

Table S9 | Mortality rates (per 100 000) for the top five Level 3 causes of death by sex and age groups in Laos, 2023

Age group (in years)	Males					Females				
	Top 1	Top 2	Top 3	Top 4	Top 5	Top 1	Top 2	Top 3	Top 4	Top 5
<1	Neonatal disorders<br>2261·2<br>(1740·6 - 2892·4)	Lower respiratory infections<br>827·6<br>(419·0 - 1304·7)	Congenital birth defects<br>603·1<br>(335·9 - 1033·5)	Diarrheal diseases<br>220·2<br>(72·8 - 473·4)	COVID-19<br>139·1<br>(86·8 - 209·4)	Neonatal disorders<br>1660·1<br>(1253·0 - 2122·2)	Lower respiratory infections<br>676·0<br>(373·5 - 1021·8)	Congenital birth defects<br>562·7<br>(302·7 - 929·2)	Diarrheal diseases<br>145·1<br>(50·6 - 327·6)	Pertussis<br>131·4<br>(12·1 - 342·7)
2-4	Lower respiratory infections<br>33·0<br>(16·5 - 52·8)	Measles<br>13·0<br>(5·4 - 23·3)	Pertussis<br>10·3<br>(0·9 - 28·4)	Congenital birth defects<br>10·1<br>(5·5 - 18·3)	Diarrheal diseases<br>9·6<br>(4·0 - 20·9)	Lower respiratory infections<br>35·4<br>(19·2 - 54·7)	Pertussis<br>13·3<br>(1·4 - 34·1)	Measles<br>12·4<br>(4·8 - 24·8)	Congenital birth defects<br>9·7<br>(5·1 - 16·4)	Drowning<br>8·8<br>(4·2 - 15·7)
5-9	Lower respiratory infections<br>21·1<br>(12·9 - 31·5)	Meningitis<br>9·3<br>(4·8 - 19·3)	Drowning<br>5·5<br>(2·9 - 10·5)	Congenital birth defects<br>4·2<br>(2·5 - 6·9)	Road injuries<br>4·2<br>(2·0 - 7·1)	Lower respiratory infections<br>23·7<br>(14·5 - 38·6)	Diarrheal diseases<br>7·3<br>(2·8 - 13·5)	Meningitis<br>7·0<br>(3·7 - 11·9)	Drowning<br>6·8<br>(3·0 - 12·3)	Pertussis<br>4·0<br>(0·4 - 12·1)
10-14	Lower respiratory infections<br>16·3<br>(10·5 - 24·1)	Road injuries<br>8·2<br>(4·0 - 12·5)	Meningitis<br>8·1<br>(4·2 - 15·5)	Drowning<br>6·8<br>(3·6 - 11·8)	Congenital birth defects<br>4·0<br>(2·4 - 6·8)	Lower respiratory infections<br>21·0<br>(12·8 - 33·1)	Drowning<br>13·4<br>(7·3 - 22·8)	Meningitis<br>7·4<br>(4·0 - 12·9)	Diarrheal diseases<br>6·3<br>(2·5 - 12·4)	Road injuries<br>5·1<br>(2·4 - 9·3)
15-19	Road injuries<br>29·7<br>(18·0 - 44·9)	Lower respiratory infections<br>15·3<br>(9·2 - 24·3)	Interpersonal violence<br>8·0<br>(2·8 - 16·6)	Drowning<br>7·6<br>(3·8 - 13·7)	Meningitis<br>7·2<br>(3·9 - 14·1)	Lower respiratory infections<br>29·2<br>(16·0 - 48·5)	Road injuries<br>20·6<br>(10·1 - 37·2)	Maternal disorders<br>13·3<br>(5·9 - 25·1)	Drowning<br>13·1<br>(6·8 - 21·6)	Meningitis<br>9·7<br>(5·4 - 15·8)
20-24	Road injuries<br>34·2<br>(20·6 - 53·7)	Lower respiratory infections<br>15·1<br>(8·4 - 23·9)	Interpersonal violence<br>12·5<br>(5·2 - 22·8)	Self-harm<br>9·4<br>(4·8 - 15·3)	Tuberculosis<br>6·5<br>(3·0 - 10·2)	Lower respiratory infections<br>20·3<br>(11·7 - 32·0)	Maternal disorders<br>16·9<br>(9·2 - 27·7)	Road injuries<br>9·6<br>(4·7 - 16·5)	Tuberculosis<br>6·7<br>(1·9 - 12·1)	Diarrheal diseases<br>5·6<br>(2·3 - 10·5)
25-29	Road injuries<br>46·8<br>(26·0 - 73·4)	Lower respiratory infections<br>23·0<br>(14·3 - 36·2)	Interpersonal violence<br>17·0<br>(7·7 - 31·9)	Tuberculosis<br>11·6<br>(5·8 - 18·2)	Self-harm<br>11·0<br>(5·6 - 18·0)	Lower respiratory infections<br>29·8<br>(16·9 - 50·9)	Maternal disorders<br>21·8<br>(10·5 - 39·0)	Road injuries<br>14·4<br>(7·7 - 23·1)	Ischemic heart disease<br>12·0<br>(6·2 - 19·0)	Chronic kidney disease<br>10·6<br>(5·8 - 17·5)
30-34	Road injuries<br>43·0<br>(25·6 - 67·0)	Lower respiratory infections<br>33·0<br>(20·5 - 54·3)	Ischemic heart disease<br>21·3<br>(11·8 - 34·7)	Stroke<br>20·0<br>(11·2 - 32·9)	Interpersonal violence<br>17·5<br>(7·9 - 31·3)	Lower respiratory infections<br>36·9<br>(20·3 - 62·2)	Ischemic heart disease<br>27·7<br>(14·8 - 43·6)	Stroke<br>25·5<br>(14·9 - 42·9)	Maternal disorders<br>25·3<br>(12·7 - 43·1)	Chronic kidney disease<br>19·0<br>(10·2 - 28·9)
35-39	Road injuries<br>46·6<br>(27·3 - 74·5)	Lower respiratory infections<br>46·3<br>(27·6 - 71·7)	Stroke<br>43·6<br>(25·9 - 69·9)	Ischemic heart disease<br>41·5<br>(23·9 - 66·4)	Cirrhosis and other chronic liver diseases	Stroke<br>43·0<br>(25·1 - 66·3)	Lower respiratory infections<br>39·3<br>(22·2 - 65·1)	Ischemic heart disease<br>36·8<br>(21·5 - 59·3)	Breast cancer<br>28·8<br>(18·6 - 42·4)	Maternal disorders<br>21·9<br>(10·6 - 39·5)

Age group (in years)	Males					Females				
	Top 1	Top 2	Top 3	Top 4	Top 5	Top 1	Top 2	Top 3	Top 4	Top 5
					25·6 (15·9 - 37·8)					
40-44	Stroke 77·9 (46·2 - 121·4)	Ischemic heart disease 71·2 (41·4 - 112·8)	Lower respiratory infections 61·0 (38·5 - 98·5)	Road injuries 45·8 (26·9 - 75·2)	Cirrhosis and other chronic liver diseases 41·2 (24·5 - 63·5)	Stroke 84·1 (53·7 - 128·0)	Ischemic heart disease 64·9 (35·6 - 103·2)	Breast cancer 53·0 (32·9 - 79·0)	Lower respiratory infections 46·5 (23·8 - 81·5)	Chronic kidney disease 32·9 (18·4 - 52·7)
45-49	Stroke 135·5 (79·7 - 201·6)	Ischemic heart disease 112·9 (65·4 - 178·7)	Lower respiratory infections 77·6 (49·9 - 118·8)	Cirrhosis and other chronic liver diseases 58·0 (36·1 - 86·3)	Road injuries 47·3 (24·5 - 80·7)	Stroke 117·7 (74·7 - 180·2)	Ischemic heart disease 80·3 (46·1 - 118·9)	Lower respiratory infections 60·2 (30·7 - 104·7)	Breast cancer 59·9 (37·3 - 87·8)	Chronic kidney disease 35·7 (21·4 - 53·5)
50-54	Stroke 221·8 (146·7 - 310·8)	Ischemic heart disease 177·6 (113·6 - 252·2)	Lower respiratory infections 114·3 (68·7 - 187·2)	Cirrhosis and other chronic liver diseases 74·7 (46·2 - 121·0)	Chronic kidney disease 63·0 (37·3 - 99·5)	Stroke 185·0 (123·0 - 268·0)	Ischemic heart disease 121·3 (76·7 - 185·2)	Lower respiratory infections 87·7 (48·1 - 148·5)	Breast cancer 74·3 (44·6 - 114·3)	Chronic kidney disease 53·1 (31·3 - 84·8)
55-59	Stroke 329·1 (213·5 - 473·5)	Ischemic heart disease 273·4 (176·3 - 403·4)	Lower respiratory infections 162·5 (103·8 - 262·3)	Chronic kidney disease 94·1 (55·9 - 143·7)	Cirrhosis and other chronic liver diseases 90·6 (56·8 - 137·2)	Stroke 282·5 (179·4 - 420·4)	Ischemic heart disease 192·1 (111·6 - 299·5)	Lower respiratory infections 134·7 (68·2 - 245·7)	Breast cancer 88·3 (56·4 - 130·7)	Chronic kidney disease 80·4 (47·2 - 121·9)
60-64	Stroke 489·9 (325·0 - 726·2)	Ischemic heart disease 376·8 (245·2 - 534·9)	Lower respiratory infections 294·3 (180·0 - 465·9)	Chronic obstructive pulmonary disease 153·5 (92·2 - 244·6)	Chronic kidney disease 123·0 (72·1 - 187·0)	Stroke 458·9 (299·5 - 665·2)	Ischemic heart disease 301·9 (188·3 - 442·1)	Lower respiratory infections 217·3 (120·5 - 380·8)	Chronic kidney disease 109·0 (66·7 - 162·7)	Diabetes mellitus 101·1 (55·0 - 167·8)
65-69	Stroke 747·4 (463·0 - 1080·0)	Ischemic heart disease 544·1 (347·1 - 844·9)	Lower respiratory infections 452·2 (274·9 - 712·0)	Chronic obstructive pulmonary disease 297·5 (180·5 - 482·2)	Chronic kidney disease 172·5 (95·2 - 266·3)	Stroke 758·0 (495·1 - 1130·6)	Ischemic heart disease 488·9 (301·8 - 751·4)	Lower respiratory infections 346·8 (182·9 - 566·6)	Chronic kidney disease 161·3 (101·4 - 246·1)	Diabetes mellitus 154·7 (82·0 - 249·0)
70-74	Stroke 1156·8 (769·5 - 1633·4)	Ischemic heart disease 778·3 (492·2 - 1130·0)	Lower respiratory infections 765·9 (468·6 - 1207·4)	Chronic obstructive pulmonary disease 570·5 (331·2 - 908·7)	Chronic kidney disease 238·7 (139·4 - 356·7)	Stroke 1287·2 (854·5 - 1869·1)	Ischemic heart disease 766·2 (464·4 - 1144·9)	Lower respiratory infections 685·7 (376·0 - 1176·4)	Diarrheal diseases 229·4 (100·8 - 454·4)	Diabetes mellitus 216·6 (115·1 - 348·2)

Age group (in years)	Males					Females				
	**Top 1**	**Top 2**	**Top 3**	**Top 4**	**Top 5**	**Top 1**	**Top 2**	**Top 3**	**Top 4**	**Top 5**
75-79	Stroke 1713·7 (1136·8 - 2349·8)	Lower respiratory infections 1238·3 (759·2 - 2024·5)	Ischemic heart disease 1110·0 (709·7 - 1602·9)	Chronic obstructive pulmonary disease 919·1 (547·4 - 1480·2)	Chronic kidney disease 327·1 (188·6 - 511·0)	Stroke 2060·2 (1360·8 - 2931·1)	Ischemic heart disease 1194·7 (719·8 - 1842·6)	Lower respiratory infections 1190·3 (659·5 - 2150·3)	Diarrheal diseases 410·7 (188·4 - 784·2)	Diabetes mellitus 292·4 (158·7 - 495·7)
80-84	Stroke 2339·0 (1505·7 - 3334·5)	Lower respiratory infections 2206·4 (1355·9 - 3508·2)	Ischemic heart disease 1545·8 (880·4 - 2330·8)	Chronic obstructive pulmonary disease 1388·2 (813·9 - 2198·7)	Chronic kidney disease 446·2 (252·3 - 699·4)	Stroke 3294·2 (2292·0 - 4579·7)	Lower respiratory infections 2208·6 (1121·4 - 3603·4)	Ischemic heart disease 1933·3 (1211·4 - 2945·2)	Diarrheal diseases 679·7 (327·5 - 1282·2)	Hypertensive heart disease 463·8 (207·7 - 790·6)
85-89	Lower respiratory infections 2964·7 (1970·7 - 4415·6)	Stroke 2798·6 (1805·1 - 3947·7)	Ischemic heart disease 1960·8 (1162·5 - 3004·4)	Chronic obstructive pulmonary disease 1901·2 (1126·8 - 2883·4)	Diarrheal diseases 631·4 (211·8 - 1444·5)	Stroke 4903·2 (3221·8 - 6689·7)	Lower respiratory infections 3301·1 (1885·1 - 5670·4)	Ischemic heart disease 3035·6 (1808·7 - 4580·6)	Diarrheal diseases 1299·8 (579·0 - 2510·8)	Alzheimer's disease and other dementias 902·5 (203·9 - 2752·8)
90-94	Lower respiratory infections 4321·6 (2727·8 - 6220·4)	Stroke 2818·2 (1740·0 - 4195·8)	Ischemic heart disease 2413·7 (1462·5 - 3729·9)	Chronic obstructive pulmonary disease 2223·4 (1338·0 - 3630·9)	Alzheimer's disease and other dementias 1185·7 (249·9 - 3434·3)	Stroke 5849·5 (3885·0 - 8673·6)	Lower respiratory infections 5165·3 (2836·9 - 9271·3)	Ischemic heart disease 4239·4 (2455·1 - 6576·7)	Diarrheal diseases 1977·7 (821·3 - 3588·3)	Alzheimer's disease and other dementias 1603·3 (306·5 - 4778·2)
95+	Lower respiratory infections 6243·5 (3722·2 - 9973·0)	Ischemic heart disease 2925·4 (1516·8 - 4826·1)	Stroke 2880·4 (1584·8 - 4904·3)	Chronic obstructive pulmonary disease 2422·8 (1490·0 - 3811·4)	Alzheimer's disease and other dementias 1986·5 (393·4 - 5482·3)	Lower respiratory infections 7279·5 (3618·6 - 13172·5)	Stroke 7008·5 (4134·8 - 10441·1)	Ischemic heart disease 5730·7 (3260·3 - 9148·5)	Diarrheal diseases 2716·2 (1221·6 - 5057·2)	Alzheimer's disease and other dementias 2429·3 (520·5 - 7682·3)

Table S10 | Mortality rates (per 100 000) for the top five Level 3 causes of death by sex and age groups in Malaysia, 2023

Age group (in years)	Males					Females				
	Top 1	Top 2	Top 3	Top 4	Top 5	Top 1	Top 2	Top 3	Top 4	Top 5
<1	Neonatal disorders 259·9 (205·7 - 327·9)	Congenital birth defects 190·4 (143·1 - 249·7)	COVID-19 71·0 (54·0 - 89·3)	Lower respiratory infections 52·5 (31·0 - 85·6)	Diarrheal diseases 9·7 (4·5 - 18·7)	Neonatal disorders 202·6 (153·1 - 258·0)	Congenital birth defects 178·1 (136·4 - 232·0)	COVID-19 58·1 (43·5 - 74·4)	Lower respiratory infections 40·8 (25·0 - 59·2)	Diarrheal diseases 8·6 (4·7 - 15·5)
2-4	COVID-19 6·4 (4·6 - 8·8)	Lower respiratory infections 4·9 (3·0 - 7·9)	Congenital birth defects 4·3 (2·8 - 6·6)	Drowning 3·2 (1·8 - 4·7)	Road injuries 2·5 (1·3 - 4·0)	Lower respiratory infections 4·7 (2·7 - 7·1)	Congenital birth defects 4·3 (2·7 - 6·4)	COVID-19 4·1 (2·9 - 5·5)	Road injuries 2·3 (1·4 - 3·6)	Drowning 1·9 (1·0 - 3·1)
5-9	Lower respiratory infections 2·3 (1·4 - 3·6)	COVID-19 2·0 (1·5 - 2·7)	Drowning 1·9 (1·1 - 2·9)	Road injuries 1·7 (0·9 - 2·9)	Congenital birth defects 1·5 (1·0 - 2·2)	COVID-19 2·4 (1·8 - 3·2)	Lower respiratory infections 2·1 (1·2 - 3·3)	Congenital birth defects 1·2 (0·8 - 1·8)	Road injuries 1·1 (0·6 - 1·8)	Drowning 0·7 (0·4 - 1·1)
10-14	Road injuries 3·8 (2·4 - 5·9)	COVID-19 2·4 (1·7 - 3·2)	Drowning 2·4 (1·3 - 3·7)	Lower respiratory infections 2·0 (1·3 - 3·1)	Congenital birth defects 1·5 (1·0 - 2·2)	Lower respiratory infections 2·2 (1·4 - 3·1)	Road injuries 2·0 (1·2 - 3·3)	COVID-19 1·7 (1·2 - 2·2)	Congenital birth defects 1·6 (1·0 - 2·4)	Drowning 1·2 (0·7 - 1·8)
15-19	Road injuries 28·3 (19·4 - 39·1)	Drowning 3·6 (2·0 - 5·8)	Lower respiratory infections 2·9 (1·7 - 4·8)	Other transport injuries 2·8 (1·2 - 4·9)	Self-harm 2·4 (1·2 - 5·1)	Road injuries 4·7 (3·0 - 6·5)	Lower respiratory infections 1·9 (1·2 - 3·0)	COVID-19 1·6 (1·1 - 2·1)	Congenital birth defects 1·1 (0·7 - 1·7)	Chronic kidney disease 0·9 (0·5 - 1·4)
20-24	Road injuries 37·6 (27·4 - 48·8)	Lower respiratory infections 3·9 (2·3 - 6·0)	Other transport injuries 3·9 (2·0 - 6·2)	Self-harm 3·9 (2·2 - 7·7)	Drowning 3·3 (2·0 - 5·0)	Road injuries 5·9 (3·8 - 8·1)	Lower respiratory infections 2·2 (1·5 - 3·3)	COVID-19 1·9 (1·4 - 2·5)	Chronic kidney disease 1·4 (0·8 - 2·2)	Maternal disorders 1·3 (0·7 - 1·9)
25-29	Road injuries 26·5 (18·1 - 37·0)	Ischemic heart disease 5·7 (3·8 - 8·1)	Lower respiratory infections 5·2 (3·4 - 8·0)	Self-harm 4·4 (2·4 - 8·3)	HIV/AIDS 3·0 (0·0 - 6·9)	Road injuries 5·7 (3·6 - 8·0)	Lower respiratory infections 3·2 (2·1 - 4·5)	Maternal disorders 2·2 (1·4 - 3·5)	Chronic kidney disease 2·1 (1·4 - 3·1)	Ischemic heart disease 1·9 (1·1 - 2·9)
30-34	Road injuries 23·5 (16·5 - 31·8)	Ischemic heart disease 14·2 (9·4 - 20·1)	Lower respiratory infections 7·9 (5·2 - 11·5)	Stroke 6·0 (3·7 - 8·6)	HIV/AIDS 5·4 (0·0 - 11·8)	Breast cancer 5·7 (4·0 - 7·6)	Road injuries 5·4 (3·7 - 7·6)	Lower respiratory infections 4·5 (2·9 - 6·6)	Ischemic heart disease 4·0 (2·5 - 5·8)	Stroke 3·4 (2·2 - 5·0)
35-39	Ischemic heart disease 32·3 (23·3 - 42·5)	Road injuries 24·5 (17·1 - 36·7)	Lower respiratory infections 13·4 (9·2 - 18·6)	Stroke 12·8 (8·0 - 18·1)	HIV/AIDS 9·2 (0·0 - 20·8)	Breast cancer 13·1 (9·9 - 16·9)	Ischemic heart disease 7·6 (5·2 - 10·9)	Stroke 7·5 (4·8 - 10·5)	Lower respiratory infections 7·3 (4·8 - 10·2)	Road injuries 6·0 (4·2 - 9·0)

Age group (in years)	Males					Females				
	Top 1	Top 2	Top 3	Top 4	Top 5	Top 1	Top 2	Top 3	Top 4	Top 5
40-44	Ischemic heart disease 71·5 (56·4 - 89·3)	Road injuries 27·2 (18·9 - 39·8)	Stroke 27·0 (18·5 - 38·9)	Lower respiratory infections 24·2 (16·1 - 32·6)	Cirrhosis and other chronic liver diseases 13·5 (9·2 - 19·0)	Breast cancer 25·8 (19·8 - 34·2)	Ischemic heart disease 17·1 (12·0 - 24·1)	Stroke 15·0 (10·2 - 20·5)	Lower respiratory infections 11·0 (6·7 - 16·2)	Chronic kidney disease 8·5 (5·4 - 12·0)
45-49	Ischemic heart disease 142·1 (114·3 - 172·4)	Stroke 47·5 (33·8 - 65·5)	Lower respiratory infections 34·4 (23·7 - 50·6)	Road injuries 32·7 (22·0 - 47·8)	Cirrhosis and other chronic liver diseases 24·3 (17·4 - 33·4)	Breast cancer 46·5 (34·8 - 59·4)	Ischemic heart disease 34·2 (23·5 - 46·8)	Stroke 30·3 (20·8 - 40·5)	Lower respiratory infections 21·0 (14·3 - 30·6)	Chronic kidney disease 14·4 (9·9 - 20·1)
50-54	Ischemic heart disease 224·3 (187·3 - 266·6)	Stroke 83·3 (61·0 - 112·3)	Lower respiratory infections 64·9 (44·0 - 87·2)	Road injuries 36·4 (26·0 - 54·1)	Cirrhosis and other chronic liver diseases 35·3 (25·6 - 46·6)	Breast cancer 61·9 (48·0 - 79·5)	Ischemic heart disease 56·5 (37·9 - 80·2)	Stroke 50·2 (35·1 - 67·6)	Lower respiratory infections 36·7 (23·7 - 51·0)	Chronic kidney disease 24·1 (16·6 - 32·5)
55-59	Ischemic heart disease 334·9 (277·4 - 396·7)	Stroke 129·7 (93·5 - 169·9)	Lower respiratory infections 95·5 (66·8 - 130·9)	Tracheal, bronchus, and lung cancer 57·2 (40·0 - 75·2)	Cirrhosis and other chronic liver diseases 47·3 (35·0 - 63·1)	Ischemic heart disease 94·9 (68·0 - 119·5)	Breast cancer 75·2 (59·2 - 95·7)	Stroke 75·0 (49·7 - 100·7)	Lower respiratory infections 63·5 (41·1 - 96·4)	Chronic kidney disease 38·8 (25·0 - 54·3)
60-64	Ischemic heart disease 453·4 (386·3 - 543·0)	Stroke 200·1 (146·9 - 260·2)	Lower respiratory infections 164·4 (115·8 - 233·0)	Tracheal, bronchus, and lung cancer 98·9 (72·3 - 129·2)	Chronic kidney disease 64·2 (38·5 - 91·7)	Ischemic heart disease 170·5 (131·7 - 214·3)	Stroke 123·6 (84·6 - 163·0)	Lower respiratory infections 107·6 (71·8 - 146·9)	Breast cancer 81·0 (62·0 - 103·1)	Chronic kidney disease 61·4 (38·6 - 85·0)
65-69	Ischemic heart disease 594·6 (485·3 - 714·2)	Stroke 288·0 (210·4 - 385·9)	Lower respiratory infections 273·7 (186·2 - 377·0)	Tracheal, bronchus, and lung cancer 143·6 (106·5 - 184·9)	Chronic kidney disease 89·7 (52·2 - 133·6)	Ischemic heart disease 270·9 (208·6 - 341·0)	Stroke 196·6 (142·9 - 252·8)	Lower respiratory infections 170·7 (111·5 - 234·8)	Chronic kidney disease 94·1 (58·2 - 133·7)	Breast cancer 84·4 (61·9 - 103·1)
70-74	Ischemic heart disease 816·2 (669·9 - 966·3)	Stroke 453·9 (338·4 - 600·8)	Lower respiratory infections 446·4 (302·2 - 630·1)	Tracheal, bronchus, and lung cancer 189·3 (138·2 - 255·0)	Chronic obstructive pulmonary disease 166·4 (121·5 - 228·1)	Ischemic heart disease 426·0 (336·9 - 537·8)	Stroke 340·5 (251·0 - 436·2)	Lower respiratory infections 317·2 (204·7 - 438·9)	Chronic kidney disease 128·9 (77·6 - 185·0)	Diabetes mellitus 116·5 (77·1 - 171·3)
75-79	Ischemic heart disease	Lower respiratory infections	Stroke 626·8 (467·6 - 823·7)	Chronic obstructive pulmonary disease	Tracheal, bronchus, and lung cancer	Ischemic heart disease	Stroke 625·2 (476·5 - 830·2)	Lower respiratory infections	Chronic kidney disease	Diabetes mellitus 161·6 (104·8 - 230·2)

Age group (in years)	Males Top 1	Males Top 2	Males Top 3	Males Top 4	Males Top 5	Females Top 1	Females Top 2	Females Top 3	Females Top 4	Females Top 5
	988·3 (797·4 - 1229·9)	735·1 (526·9 - 1020·7)		279·1 (203·5 - 368·9)	212·8 (157·4 - 280·6)	704·7 (529·3 - 883·7)		577·7 (387·7 - 793·3)	196·2 (113·5 - 282·0)	
80-84	Ischemic heart disease 1716·5 (1356·3 - 2173·0)	Lower respiratory infections 1202·1 (838·0 - 1689·2)	Stroke 1184·7 (862·5 - 1518·8)	Chronic obstructive pulmonary disease 448·8 (327·9 - 571·6)	Chronic kidney disease 335·2 (226·2 - 471·0)	Ischemic heart disease 1464·5 (1069·1 - 1915·3)	Stroke 1315·1 (958·2 - 1745·2)	Lower respiratory infections 1270·9 (820·1 - 1838·8)	Alzheimer's disease and other dementias 526·1 (120·8 - 1374·3)	Chronic kidney disease 327·8 (186·0 - 479·4)
85-89	Ischemic heart disease 2521·9 (1763·3 - 3509·1)	Lower respiratory infections 2077·9 (1276·9 - 3097·8)	Stroke 1701·4 (1101·3 - 2389·6)	Alzheimer's disease and other dementias 748·5 (141·4 - 2144·5)	Chronic obstructive pulmonary disease 708·8 (462·2 - 1044·5)	Ischemic heart disease 2678·8 (1832·0 - 3637·1)	Lower respiratory infections 2350·8 (1418·5 - 3551·4)	Stroke 2315·6 (1451·8 - 3282·9)	Alzheimer's disease and other dementias 1230·6 (285·1 - 3481·0)	Chronic kidney disease 544·0 (291·2 - 844·0)
90-94	Lower respiratory infections 3314·7 (2071·3 - 4894·7)	Ischemic heart disease 3252·2 (2008·7 - 4859·5)	Stroke 2017·5 (1242·5 - 2906·4)	Alzheimer's disease and other dementias 1536·0 (303·7 - 4317·9)	Chronic obstructive pulmonary disease 947·5 (576·7 - 1372·1)	Lower respiratory infections 4139·8 (2591·1 - 6193·0)	Ischemic heart disease 3979·7 (2514·8 - 5914·7)	Stroke 3152·4 (1840·7 - 4793·6)	Alzheimer's disease and other dementias 2457·9 (559·5 - 6483·7)	Chronic kidney disease 726·2 (340·7 - 1109·4)
95+	Lower respiratory infections 5029·4 (3057·5 - 7938·5)	Ischemic heart disease 4451·5 (2625·0 - 6784·0)	Alzheimer's disease and other dementias 2684·9 (515·6 - 7196·7)	Stroke 2568·5 (1594·0 - 3864·6)	Chronic obstructive pulmonary disease 1229·5 (759·6 - 1863·5)	Lower respiratory infections 6205·0 (3742·7 - 10039·1)	Ischemic heart disease 5756·0 (3571·7 - 9387·7)	Stroke 4252·7 (2091·6 - 6670·6)	Alzheimer's disease and other dementias 4244·0 (973·2 - 11583·2)	Urinary diseases and male infertility 974·2 (653·7 - 1386·5)

Table S11 | Mortality rates (per 100 000) for the top five Level 3 causes of death by sex and age groups in Myanmar, 2023

Age group (in years)	Males Top 1	Males Top 2	Males Top 3	Males Top 4	Males Top 5	Females Top 1	Females Top 2	Females Top 3	Females Top 4	Females Top 5
<1	Neonatal disorders 1953·6 (1502·6 - 2454·6)	Lower respiratory infections 662·7 (353·6 - 1106·4)	Congenital birth defects 535·5 (290·6 - 877·6)	Diarrheal diseases 206·8 (80·1 - 481·6)	Paralytic ileus and intestinal obstruction 124·3 (64·0 - 206·1)	Neonatal disorders 1384·2 (1036·1 - 1772·1)	Lower respiratory infections 578·9 (332·4 - 855·9)	Congenital birth defects 527·8 (307·2 - 868·4)	Pertussis 97·2 (10·3 - 247·1)	Meningitis 81·6 (31·7 - 179·8)
2-4	Drowning 31·7 (7·3 - 56·0)	Measles 16·5 (6·3 - 34·1)	Congenital birth defects 13·0 (7·6 - 23·3)	Lower respiratory infections 13·0 (6·5 - 23·6)	Pertussis 12·8 (1·4 - 37·4)	Lower respiratory infections 20·2 (11·4 - 32·5)	Pertussis 13·9 (1·8 - 38·0)	Measles 13·4 (5·2 - 27·4)	Congenital birth defects 11·5 (5·8 - 22·2)	Meningitis 8·3 (3·5 - 18·0)
5-9	Conflict and terrorism 12·6 (4·9 - 27·3)	Drowning 11·7 (5·1 - 19·6)	Lower respiratory infections 9·5 (5·5 - 15·0)	Congenital birth defects 4·8 (2·8 - 8·0)	Typhoid and paratyphoid 4·4 (1·8 - 9·4)	Conflict and terrorism 7·7 (3·0 - 16·6)	Lower respiratory infections 5·9 (3·2 - 10·8)	Pertussis 5·4 (0·6 - 14·2)	Congenital birth defects 5·3 (2·9 - 8·5)	Drowning 5·1 (2·8 - 8·6)
10-14	Drowning 6·6 (3·8 - 10·8)	Lower respiratory infections 5·2 (3·1 - 8·0)	Road injuries 4·8 (2·2 - 8·4)	Congenital birth defects 3·6 (2·3 - 5·9)	Conflict and terrorism 3·2 (1·3 - 7·0)	Conflict and terrorism 7·1 (2·8 - 15·3)	Congenital birth defects 4·3 (2·4 - 7·0)	Drowning 3·2 (1·7 - 5·8)	Lower respiratory infections 3·0 (1·8 - 5·7)	Leukemia 2·2 (1·4 - 3·2)
15-19	Conflict and terrorism 132·2 (51·4 - 285·7)	Road injuries 29·7 (17·7 - 47·5)	Drowning 10·5 (5·3 - 17·4)	Falls 6·4 (3·2 - 11·6)	Self-harm 5·5 (2·9 - 9·1)	Road injuries 14·5 (6·5 - 24·0)	Conflict and terrorism 13·3 (5·2 - 28·7)	Maternal disorders 8·9 (4·8 - 15·4)	Chronic kidney disease 7·2 (4·0 - 12·0)	Ischemic heart disease 6·2 (3·4 - 10·5)
20-24	Conflict and terrorism 103·6 (40·2 - 223·8)	Road injuries 34·4 (18·6 - 53·1)	Tuberculosis 9·3 (4·8 - 16·6)	Self-harm 9·2 (4·9 - 14·9)	Drowning 7·4 (4·2 - 12·1)	Maternal disorders 28·0 (14·3 - 49·2)	Road injuries 22·1 (10·1 - 34·6)	Conflict and terrorism 17·5 (6·8 - 37·8)	Chronic kidney disease 15·6 (8·0 - 25·6)	Ischemic heart disease 15·0 (7·7 - 23·5)
25-29	Road injuries 39·8 (20·2 - 65·8)	Conflict and terrorism 27·8 (10·8 - 60·0)	Tuberculosis 19·8 (11·2 - 38·3)	Cirrhosis and other chronic liver diseases 19·1 (9·7 - 32·1)	Alcohol use disorders 15·8 (8·2 - 28·5)	Maternal disorders 22·7 (12·3 - 38·3)	HIV/AIDS 21·4 (5·4 - 44·2)	Ischemic heart disease 15·4 (8·6 - 24·7)	Road injuries 13·2 (5·3 - 23·1)	Chronic kidney disease 12·4 (6·9 - 21·3)
30-34	Cirrhosis and other chronic liver diseases 73·0 (38·5 - 118·4)	Road injuries 52·5 (25·3 - 90·0)	Alcohol use disorders 48·2 (23·8 - 84·9)	Stroke 41·5 (23·5 - 64·1)	Tuberculosis 37·6 (19·2 - 64·7)	HIV/AIDS 35·4 (9·4 - 68·9)	Ischemic heart disease 31·5 (17·2 - 53·0)	Maternal disorders 27·9 (15·3 - 47·1)	Breast cancer 22·4 (12·7 - 33·6)	Stroke 20·8 (10·9 - 36·3)

Age group (in years)	Males					Females				
	Top 1	Top 2	Top 3	Top 4	Top 5	Top 1	Top 2	Top 3	Top 4	Top 5
35-39	Cirrhosis and other chronic liver diseases 140·9 (83·7 - 218·7)	Alcohol use disorders 86·7 (43·2 - 145·4)	Stroke 84·1 (50·3 - 128·7)	Ischemic heart disease 55·5 (29·8 - 94·9)	Road injuries 53·9 (23·1 - 89·5)	HIV/AIDS 32·8 (6·6 - 69·7)	Ischemic heart disease 27·5 (14·0 - 47·8)	Stroke 23·2 (12·7 - 39·0)	Breast cancer 22·6 (14·2 - 33·1)	Maternal disorders 14·8 (7·1 - 25·9)
40-44	Cirrhosis and other chronic liver diseases 156·2 (89·4 - 237·8)	Stroke 122·6 (75·0 - 185·0)	Alcohol use disorders 87·7 (46·5 - 150·1)	Ischemic heart disease 70·3 (40·8 - 117·9)	Tuberculosis 47·5 (26·8 - 85·2)	Ischemic heart disease 50·1 (29·0 - 80·2)	Stroke 48·8 (28·4 - 75·0)	Breast cancer 39·3 (25·1 - 55·6)	HIV/AIDS 33·3 (5·5 - 62·1)	Diabetes mellitus 20·5 (8·0 - 42·8)
45-49	Stroke 185·3 (113·7 - 275·5)	Cirrhosis and other chronic liver diseases 156·9 (93·0 - 232·5)	Ischemic heart disease 92·2 (53·6 - 147·2)	Alcohol use disorders 85·2 (41·7 - 155·1)	Tuberculosis 49·1 (28·7 - 78·5)	Stroke 78·8 (46·9 - 119·2)	Ischemic heart disease 68·0 (41·7 - 102·3)	Breast cancer 45·6 (29·9 - 63·3)	Diabetes mellitus 30·7 (14·8 - 56·5)	HIV/AIDS 28·8 (6·8 - 57·1)
50-54	Stroke 359·9 (228·1 - 512·6)	Ischemic heart disease 167·7 (99·3 - 261·4)	Cirrhosis and other chronic liver diseases 135·3 (78·5 - 206·3)	Alcohol use disorders 88·7 (44·0 - 160·0)	Diabetes mellitus 76·9 (44·2 - 118·9)	Stroke 148·8 (100·2 - 216·0)	Ischemic heart disease 100·0 (57·7 - 159·6)	Breast cancer 57·5 (36·3 - 81·7)	Diabetes mellitus 50·9 (26·5 - 87·7)	Chronic kidney disease 28·7 (16·4 - 45·5)
55-59	Stroke 610·0 (405·2 - 918·8)	Ischemic heart disease 263·6 (159·3 - 384·9)	Cirrhosis and other chronic liver diseases 128·2 (74·0 - 201·0)	Diabetes mellitus 110·2 (63·4 - 194·5)	Chronic obstructive pulmonary disease 103·1 (65·5 - 159·2)	Stroke 248·3 (158·0 - 369·0)	Ischemic heart disease 173·6 (107·9 - 264·7)	Diabetes mellitus 86·4 (45·8 - 142·7)	Breast cancer 72·6 (44·0 - 102·2)	Chronic obstructive pulmonary disease 52·4 (19·8 - 98·4)
60-64	Stroke 953·5 (665·5 - 1386·0)	Ischemic heart disease 368·7 (225·6 - 540·4)	Chronic obstructive pulmonary disease 204·8 (121·1 - 309·3)	Diabetes mellitus 158·1 (90·3 - 263·4)	Hypertensive heart disease 142·4 (62·4 - 255·7)	Stroke 416·9 (264·2 - 596·7)	Ischemic heart disease 279·5 (188·6 - 414·9)	Diabetes mellitus 143·3 (80·5 - 227·4)	Chronic obstructive pulmonary disease 125·8 (47·0 - 211·8)	Breast cancer 70·9 (45·1 - 107·0)
65-69	Stroke 1465·4 (963·2 - 2083·0)	Ischemic heart disease 525·6 (335·4 - 823·4)	Chronic obstructive pulmonary disease 382·9 (240·8 - 592·3)	Diabetes mellitus 231·9 (130·7 - 357·5)	Hypertensive heart disease 216·5 (95·2 - 387·7)	Stroke 679·4 (456·7 - 975·6)	Ischemic heart disease 469·7 (279·8 - 677·5)	Chronic obstructive pulmonary disease 244·6 (92·6 - 413·9)	Diabetes mellitus 224·2 (129·3 - 381·1)	Hypertensive heart disease 88·0 (34·6 - 149·9)

Age group (in years)	Males					Females				
	Top 1	Top 2	Top 3	Top 4	Top 5	Top 1	Top 2	Top 3	Top 4	Top 5
70-74	Stroke 2291·0 (1533·2 - 3147·8)	Ischemic heart disease 754·1 (473·7 - 1171·8)	Chronic obstructive pulmonary disease 698·1 (436·5 - 1084·6)	Hypertensive heart disease 352·9 (159·9 - 646·0)	Diabetes mellitus 302·1 (168·1 - 483·6)	Stroke 1218·1 (781·2 - 1767·3)	Ischemic heart disease 784·2 (477·7 - 1200·8)	Chronic obstructive pulmonary disease 442·1 (154·7 - 741·8)	Diabetes mellitus 301·0 (176·0 - 483·3)	Hypertensive heart disease 169·9 (73·7 - 306·5)
75-79	Stroke 3311·8 (2385·9 - 4461·3)	Chronic obstructive pulmonary disease 1156·2 (716·1 - 1847·0)	Ischemic heart disease 1087·6 (684·9 - 1767·9)	Hypertensive heart disease 521·6 (228·9 - 930·4)	Diabetes mellitus 425·9 (234·2 - 694·0)	Stroke 2018·2 (1333·1 - 2858·9)	Ischemic heart disease 1292·1 (747·3 - 1954·1)	Chronic obstructive pulmonary disease 750·6 (278·2 - 1199·6)	Diabetes mellitus 415·8 (230·7 - 688·1)	Hypertensive heart disease 286·2 (118·1 - 509·3)
80-84	Stroke 4308·8 (2870·8 - 6050·5)	Chronic obstructive pulmonary disease 1938·8 (1167·7 - 2910·8)	Ischemic heart disease 1659·0 (1030·6 - 2635·2)	Hypertensive heart disease 685·4 (324·2 - 1197·8)	Lower respiratory infections 651·7 (395·3 - 1070·4)	Stroke 3165·1 (1971·3 - 4728·2)	Ischemic heart disease 2240·7 (1337·0 - 3265·6)	Chronic obstructive pulmonary disease 1331·9 (467·8 - 2137·1)	Diabetes mellitus 540·3 (308·1 - 917·0)	Hypertensive heart disease 472·7 (217·7 - 790·2)
85-89	Stroke 4315·9 (2753·5 - 6219·2)	Chronic obstructive pulmonary disease 3297·8 (2114·8 - 4934·2)	Ischemic heart disease 2219·3 (1295·4 - 3396·4)	Lower respiratory infections 1014·2 (602·5 - 1560·1)	Diabetes mellitus 828·5 (470·9 - 1355·5)	Stroke 4300·5 (2583·5 - 6564·6)	Ischemic heart disease 3497·1 (2108·3 - 5424·1)	Chronic obstructive pulmonary disease 2506·8 (849·3 - 4041·1)	Alzheimer's disease and other dementias 975·3 (234·2 - 2596·6)	Lower respiratory infections 785·0 (378·8 - 1376·0)
90-94	Chronic obstructive pulmonary disease 4381·2 (2765·5 - 6375·7)	Stroke 3680·3 (2170·7 - 5610·4)	Ischemic heart disease 2944·9 (1750·5 - 4422·7)	Lower respiratory infections 1665·4 (1008·1 - 2682·9)	Alzheimer's disease and other dementias 1476·9 (308·4 - 4019·7)	Ischemic heart disease 5162·9 (3064·9 - 8162·2)	Stroke 4974·1 (2866·6 - 7892·6)	Chronic obstructive pulmonary disease 3437·2 (1280·3 - 5529·3)	Alzheimer's disease and other dementias 1866·2 (394·6 - 4849·4)	Lower respiratory infections 1391·1 (733·9 - 2610·0)
95+	Chronic obstructive pulmonary disease 5852·6 (3621·0 - 9010·1)	Ischemic heart disease 3331·9 (1746·2 - 5678·9)	Stroke 2837·6 (1369·1 - 4827·1)	Alzheimer's disease and other dementias 2805·1 (600·8 - 8080·8)	Lower respiratory infections 2781·3 (1644·2 - 4456·9)	Ischemic heart disease 6541·6 (3582·7 - 10854·5)	Stroke 5371·2 (2680·8 - 8789·7)	Chronic obstructive pulmonary disease 4660·5 (1974·5 - 7478·5)	Alzheimer's disease and other dementias 3503·2 (843·8 - 9432·3)	Lower respiratory infections 2603·4 (1214·3 - 4911·3)

Table S12 | Mortality rates (per 100 000) for the top five Level 3 causes of death by sex and age groups in Philippines, 2023

Age group (in years)	Males					Females				
	Top 1	Top 2	Top 3	Top 4	Top 5	Top 1	Top 2	Top 3	Top 4	Top 5
<1	Neonatal disorders 1110·0 (903·1 - 1337·6)	Congenital birth defects 337·2 (248·6 - 441·2)	Lower respiratory infections 265·7 (194·0 - 364·9)	COVID-19 171·4 (136·5 - 208·7)	Diarrheal diseases 97·5 (56·6 - 159·4)	Neonatal disorders 860·7 (710·8 - 1034·0)	Congenital birth defects 301·4 (223·6 - 399·7)	Lower respiratory infections 212·2 (160·1 - 262·5)	COVID-19 115·4 (94·1 - 146·2)	Diarrheal diseases 62·7 (38·2 - 100·5)
2-4	Lower respiratory infections 17·9 (11·1 - 25·7)	Drowning 11·7 (6·9 - 17·2)	Congenital birth defects 10·0 (6·7 - 15·4)	Diarrheal diseases 8·5 (4·8 - 14·6)	COVID-19 7·9 (5·9 - 10·4)	Lower respiratory infections 20·7 (13·5 - 29·8)	Congenital birth defects 11·6 (7·4 - 17·2)	Diarrheal diseases 8·7 (5·2 - 14·0)	Drowning 7·0 (4·1 - 10·6)	Typhoid and paratyphoid 7·0 (3·4 - 13·6)
5-9	Lower respiratory infections 7·1 (5·1 - 10·4)	Drowning 6·7 (4·6 - 9·0)	Congenital birth defects 4·7 (3·3 - 6·4)	Typhoid and paratyphoid 3·8 (1·7 - 6·9)	Road injuries 3·1 (1·9 - 4·8)	Lower respiratory infections 6·2 (3·9 - 8·8)	Congenital birth defects 4·5 (3·1 - 6·1)	Drowning 3·8 (2·4 - 5·5)	Typhoid and paratyphoid 3·6 (1·6 - 6·5)	COVID-19 2·7 (2·2 - 3·4)
10-14	Lower respiratory infections 4·8 (3·7 - 6·4)	Drowning 4·7 (3·5 - 6·0)	Road injuries 4·0 (2·5 - 5·7)	Congenital birth defects 3·9 (2·9 - 5·2)	COVID-19 2·9 (2·5 - 3·3)	Lower respiratory infections 3·8 (2·6 - 5·4)	Congenital birth defects 3·6 (2·5 - 4·8)	Drowning 2·6 (1·7 - 3·9)	Leukemia 2·1 (1·6 - 2·9)	Typhoid and paratyphoid 2·1 (0·8 - 4·1)
15-19	Road injuries 15·2 (10·4 - 19·9)	Interpersonal violence 6·8 (4·9 - 8·9)	Self-harm 5·8 (2·6 - 8·5)	Lower respiratory infections 4·8 (3·5 - 6·4)	Tuberculosis 4·4 (3·2 - 6·0)	Lower respiratory infections 3·4 (2·2 - 4·8)	Tuberculosis 3·1 (2·1 - 4·2)	Road injuries 2·9 (1·9 - 4·5)	Self-harm 2·7 (1·8 - 3·6)	Maternal disorders 2·6 (2·1 - 3·4)
20-24	Road injuries 24·6 (17·4 - 31·6)	Interpersonal violence 13·4 (10·4 - 16·9)	Self-harm 9·1 (4·5 - 12·2)	Ischemic heart disease 8·8 (6·6 - 11·5)	Tuberculosis 7·3 (5·7 - 9·2)	Maternal disorders 5·9 (4·8 - 7·7)	Tuberculosis 4·6 (3·4 - 6·1)	Lower respiratory infections 4·1 (2·8 - 6·1)	Chronic kidney disease 3·7 (2·7 - 5·0)	Ischemic heart disease 3·6 (2·4 - 5·0)
25-29	Road injuries 23·9 (15·7 - 30·5)	Interpersonal violence 18·9 (15·3 - 22·8)	Ischemic heart disease 17·6 (13·9 - 22·6)	Tuberculosis 11·5 (8·8 - 14·8)	Self-harm 9·3 (5·8 - 12·4)	Maternal disorders 7·9 (6·3 - 10·2)	Tuberculosis 6·1 (4·5 - 8·0)	Ischemic heart disease 5·9 (4·1 - 7·8)	Chronic kidney disease 5·2 (3·9 - 6·7)	Lower respiratory infections 5·1 (3·4 - 7·5)
30-34	Ischemic heart disease 30·9 (25·3 - 37·5)	Interpersonal violence 24·2 (19·1 - 29·5)	Road injuries 22·2 (14·8 - 29·9)	Tuberculosis 14·6 (10·7 - 18·8)	Stroke 12·6 (9·2 - 16·8)	Ischemic heart disease 10·5 (7·7 - 13·7)	Maternal disorders 10·0 (8·1 - 13·5)	Chronic kidney disease 7·4 (5·0 - 9·8)	Tuberculosis 7·3 (5·4 - 9·7)	Breast cancer 6·8 (5·2 - 8·7)
35-39	Ischemic heart disease 52·8 (44·7 - 62·4)	Interpersonal violence 29·1 (22·0 - 38·3)	Stroke 25·6 (19·8 - 33·4)	Road injuries 22·0 (14·0 - 29·5)	Tuberculosis 19·2 (14·5 - 24·0)	Ischemic heart disease 18·4 (13·5 - 23·5)	Breast cancer 16·3 (12·9 - 19·9)	Stroke 11·8 (7·9 - 16·1)	Maternal disorders 11·3 (9·4 - 15·2)	Chronic kidney disease 10·5 (7·5 - 13·9)

Age group (in years)	Males					Females				
	Top 1	Top 2	Top 3	Top 4	Top 5	Top 1	Top 2	Top 3	Top 4	Top 5
40-44	Ischemic heart disease 93·0 (80·2 - 107·3)	Stroke 50·3 (41·3 - 62·4)	Interpersonal violence 29·7 (22·8 - 38·1)	Cirrhosis and other chronic liver diseases 26·8 (20·5 - 34·8)	Tuberculosis 25·8 (20·1 - 33·4)	Ischemic heart disease 31·7 (23·9 - 39·4)	Breast cancer 30·8 (25·1 - 36·6)	Stroke 22·4 (15·4 - 29·2)	Chronic kidney disease 15·3 (11·7 - 19·4)	Diabetes mellitus 11·4 (7·3 - 17·0)
45-49	Ischemic heart disease 152·1 (131·8 - 169·7)	Stroke 84·9 (69·6 - 102·3)	Cirrhosis and other chronic liver diseases 40·1 (30·9 - 50·1)	Diabetes mellitus 37·4 (27·7 - 49·3)	Tuberculosis 36·9 (28·7 - 46·1)	Ischemic heart disease 54·8 (43·0 - 66·9)	Breast cancer 49·9 (42·0 - 59·0)	Stroke 42·4 (30·3 - 53·5)	Chronic kidney disease 22·8 (17·6 - 29·0)	Diabetes mellitus 21·8 (13·7 - 31·2)
50-54	Ischemic heart disease 244·7 (216·4 - 273·3)	Stroke 135·9 (112·7 - 162·8)	Diabetes mellitus 70·2 (53·0 - 91·5)	Chronic kidney disease 55·8 (39·9 - 72·0)	Cirrhosis and other chronic liver diseases 55·0 (43·4 - 67·6)	Ischemic heart disease 89·1 (71·7 - 108·0)	Stroke 67·5 (50·0 - 84·0)	Breast cancer 67·0 (55·6 - 79·1)	Diabetes mellitus 42·0 (25·9 - 58·6)	Chronic kidney disease 35·3 (28·3 - 44·9)
55-59	Ischemic heart disease 364·6 (322·2 - 405·6)	Stroke 201·1 (166·8 - 236·5)	Diabetes mellitus 113·4 (87·5 - 150·6)	Chronic kidney disease 85·7 (60·5 - 110·5)	Tuberculosis 72·3 (57·6 - 89·0)	Ischemic heart disease 138·7 (112·7 - 164·3)	Stroke 96·1 (76·1 - 118·2)	Breast cancer 78·8 (66·6 - 91·3)	Diabetes mellitus 73·9 (49·0 - 105·1)	Chronic kidney disease 54·2 (42·0 - 70·3)
60-64	Ischemic heart disease 501·3 (446·6 - 553·2)	Stroke 284·6 (240·5 - 337·2)	Diabetes mellitus 160·4 (121·6 - 212·9)	Lower respiratory infections 121·0 (98·1 - 150·5)	Chronic kidney disease 113·5 (81·9 - 148·1)	Ischemic heart disease 214·3 (176·9 - 250·7)	Stroke 143·6 (113·1 - 173·8)	Diabetes mellitus 118·0 (81·4 - 158·2)	Breast cancer 78·6 (64·8 - 93·1)	Chronic kidney disease 74·1 (58·5 - 94·0)
65-69	Ischemic heart disease 747·8 (666·2 - 833·4)	Stroke 435·1 (363·0 - 516·4)	Diabetes mellitus 234·5 (179·4 - 309·8)	Lower respiratory infections 217·1 (176·5 - 265·5)	Chronic obstructive pulmonary disease 188·2 (150·5 - 242·8)	Ischemic heart disease 352·1 (294·0 - 418·4)	Stroke 237·3 (191·0 - 295·7)	Diabetes mellitus 180·3 (124·2 - 246·7)	Lower respiratory infections 109·7 (79·5 - 149·3)	Chronic kidney disease 109·1 (87·0 - 143·1)
70-74	Ischemic heart disease 1082·5 (965·3 - 1219·9)	Stroke 650·0 (545·2 - 772·6)	Lower respiratory infections 415·6 (342·1 - 502·1)	Chronic obstructive pulmonary disease 363·6 (294·9 - 458·5)	Diabetes mellitus 301·0 (228·8 - 393·7)	Ischemic heart disease 572·9 (470·4 - 679·5)	Stroke 408·1 (333·8 - 499·9)	Diabetes mellitus 257·9 (180·3 - 349·1)	Lower respiratory infections 228·3 (171·5 - 299·5)	Chronic kidney disease 150·8 (112·2 - 196·0)
75-79	Ischemic heart disease 1629·1 (1462·5 - 1826·2)	Stroke 976·9 (815·7 - 1172·5)	Lower respiratory infections 760·5 (626·8 - 929·6)	Chronic obstructive pulmonary disease	Diabetes mellitus 390·8 (295·9 - 527·1)	Ischemic heart disease 962·7 (778·4 - 1140·1)	Stroke 680·8 (567·5 - 824·8)	Lower respiratory infections 461·3 (350·1 - 600·3)	Diabetes mellitus 351·3 (235·5 - 481·4)	Hypertensive heart disease 243·4 (171·2 - 338·0)

Age group (in years)	Males					Females				
	Top 1	Top 2	Top 3	Top 4	Top 5	Top 1	Top 2	Top 3	Top 4	Top 5
				618·6 (499·6 - 772·4)						
80-84	Ischemic heart disease 2481·9 (2203·7 - 2817·0)	Lower respiratory infections 1460·6 (1202·7 - 1764·4)	Stroke 1341·1 (1119·5 - 1639·9)	Chronic obstructive pulmonary disease 936·4 (747·0 - 1183·6)	Diabetes mellitus 466·9 (343·5 - 636·1)	Ischemic heart disease 1762·9 (1420·2 - 2096·1)	Stroke 1118·9 (880·6 - 1389·5)	Lower respiratory infections 1002·8 (744·8 - 1299·6)	Alzheimer's disease and other dementias 454·4 (110·9 - 1194·1)	Diabetes mellitus 453·8 (310·0 - 629·4)
85-89	Ischemic heart disease 4680·3 (4017·9 - 5258·8)	Lower respiratory infections 2508·2 (2065·3 - 3064·5)	Stroke 2300·6 (1820·1 - 2878·2)	Chronic obstructive pulmonary disease 1273·6 (1007·8 - 1671·2)	Chronic kidney disease 905·6 (641·4 - 1236·2)	Ischemic heart disease 3494·6 (2741·4 - 4095·7)	Lower respiratory infections 2042·9 (1515·4 - 2588·4)	Stroke 1912·6 (1502·5 - 2489·7)	Alzheimer's disease and other dementias 994·1 (242·6 - 2823·1)	Hypertensive heart disease 814·8 (580·4 - 1276·9)
90-94	Ischemic heart disease 5584·7 (4563·4 - 6545·7)	Lower respiratory infections 4111·7 (3304·2 - 5220·0)	Stroke 2173·2 (1597·5 - 2890·4)	Chronic obstructive pulmonary disease 1447·6 (1047·3 - 1896·3)	Alzheimer's disease and other dementias 1434·0 (311·3 - 4061·2)	Ischemic heart disease 5294·8 (4122·2 - 6384·2)	Lower respiratory infections 3873·2 (2600·1 - 5419·8)	Stroke 2310·8 (1732·7 - 3122·3)	Alzheimer's disease and other dementias 1950·3 (458·0 - 5537·3)	Hypertensive heart disease 1061·0 (719·7 - 1661·5)
95+	Ischemic heart disease 7059·5 (5128·0 - 9359·4)	Lower respiratory infections 6491·6 (4510·0 - 8867·6)	Alzheimer's disease and other dementias 2641·7 (574·9 - 7246·4)	Stroke 2329·0 (1544·5 - 3539·4)	Chronic obstructive pulmonary disease 1772·1 (1195·4 - 2624·5)	Ischemic heart disease 7635·1 (5192·3 - 10327·6)	Lower respiratory infections 6123·8 (3850·1 - 8891·5)	Alzheimer's disease and other dementias 3326·9 (680·2 - 9317·1)	Stroke 2787·9 (1874·4 - 4079·3)	Hypertensive heart disease 1251·1 (716·5 - 1910·9)

Table S13 | Mortality rates (per 100 000) for the top five Level 3 causes of death by sex and age groups in Singapore, 2023

Age group (in years)	Males					Females				
	Top 1	Top 2	Top 3	Top 4	Top 5	Top 1	Top 2	Top 3	Top 4	Top 5
<1	Congenital birth defects 70·6 (58·1 - 100·9)	Neonatal disorders 55·3 (40·6 - 64·5)	Lower respiratory infections 13·7 (8·0 - 17·9)	COVID-19 2·7 (1·8 - 3·8)	Endocrine, metabolic, blood, and immune disorders 2·4 (1·6 - 3·1)	Congenital birth defects 88·3 (66·8 - 117·0)	Neonatal disorders 50·9 (31·9 - 65·3)	Lower respiratory infections 9·3 (4·2 - 14·4)	COVID-19 2·1 (1·3 - 3·4)	Foreign body 1·7 (1·1 - 2·4)
2-4	Congenital birth defects 0·8 (0·6 - 1·7)	COVID-19 0·4 (0·2 - 0·6)	Leukemia 0·3 (0·2 - 0·3)	Lower respiratory infections 0·3 (0·2 - 0·4)	Brain and central nervous system cancer 0·2 (0·1 - 0·3)	Congenital birth defects 2·0 (1·2 - 3·4)	Lower respiratory infections 0·7 (0·3 - 1·0)	Leukemia 0·4 (0·3 - 0·6)	Brain and central nervous system cancer 0·3 (0·2 - 0·5)	COVID-19 0·2 (0·1 - 0·3)
5-9	Congenital birth defects 0·9 (0·6 - 1·4)	Brain and central nervous system cancer 0·8 (0·6 - 1·0)	Leukemia 0·7 (0·5 - 0·9)	Lower respiratory infections 0·6 (0·4 - 0·8)	Drowning 0·3 (0·2 - 0·5)	Congenital birth defects 0·9 (0·5 - 1·5)	Lower respiratory infections 0·8 (0·6 - 1·0)	Brain and central nervous system cancer 0·5 (0·4 - 0·7)	Leukemia 0·5 (0·3 - 0·6)	COVID-19 0·3 (0·2 - 0·4)
10-14	Leukemia 1·5 (1·1 - 1·8)	Self-harm 1·5 (1·2 - 2·0)	Brain and central nervous system cancer 1·3 (1·1 - 1·6)	Congenital birth defects 1·2 (0·9 - 1·7)	Lower respiratory infections 0·9 (0·6 - 1·2)	Congenital birth defects 1·0 (0·6 - 1·4)	Leukemia 1·0 (0·8 - 1·3)	Self-harm 1·0 (0·8 - 1·3)	Brain and central nervous system cancer 0·9 (0·7 - 1·2)	Lower respiratory infections 0·8 (0·6 - 1·0)
15-19	Self-harm 10·8 (9·1 - 12·9)	Road injuries 3·0 (2·3 - 3·7)	Leukemia 1·5 (1·1 - 1·9)	Brain and central nervous system cancer 1·2 (1·0 - 1·6)	Lower respiratory infections 1·1 (0·8 - 1·4)	Self-harm 7·7 (6·4 - 9·0)	Leukemia 1·4 (1·1 - 1·7)	Lower respiratory infections 1·0 (0·7 - 1·3)	Brain and central nervous system cancer 0·8 (0·6 - 1·0)	Congenital birth defects 0·7 (0·4 - 1·2)
20-24	Self-harm 21·4 (18·3 - 24·7)	Road injuries 8·2 (6·7 - 9·9)	Leukemia 2·0 (1·5 - 2·5)	Falls 1·8 (1·4 - 2·3)	Ischemic heart disease 1·8 (1·3 - 2·4)	Self-harm 7·0 (6·0 - 8·2)	Leukemia 1·0 (0·8 - 1·2)	Lower respiratory infections 0·7 (0·5 - 0·9)	Road injuries 0·6 (0·4 - 0·7)	Brain and central nervous system cancer 0·5 (0·4 - 0·6)
25-29	Self-harm 27·0 (23·0 - 31·6)	Road injuries 8·2 (6·9 - 9·9)	Ischemic heart disease 3·7 (2·8 - 4·7)	Falls 2·6 (2·0 - 3·2)	Leukemia 2·5 (2·0 - 3·1)	Self-harm 6·2 (5·3 - 7·3)	Breast cancer 0·9 (0·7 - 1·2)	Leukemia 0·8 (0·6 - 0·9)	Lower respiratory infections 0·7 (0·5 - 0·9)	Ischemic heart disease 0·6 (0·3 - 0·8)

Age group (in years)	Males					Females				
	Top 1	Top 2	Top 3	Top 4	Top 5	Top 1	Top 2	Top 3	Top 4	Top 5
30-34	Self-harm 14·0 (11·9 - 16·7)	Ischemic heart disease 6·7 (5·4 - 8·1)	Road injuries 3·3 (2·7 - 4·0)	Falls 1·6 (1·3 - 2·0)	Leukemia 1·6 (1·3 - 2·0)	Self-harm 4·0 (3·4 - 4·8)	Breast cancer 2·4 (2·0 - 2·9)	Ischemic heart disease 0·9 (0·4 - 1·2)	Colon and rectum cancer 0·8 (0·6 - 0·9)	Leukemia 0·8 (0·6 - 1·0)
35-39	Ischemic heart disease 13·6 (11·4 - 16·1)	Self-harm 13·3 (11·0 - 15·3)	Stroke 2·7 (2·0 - 3·4)	Colon and rectum cancer 2·0 (1·6 - 2·4)	Road injuries 2·0 (1·6 - 2·4)	Breast cancer 5·1 (4·1 - 6·1)	Self-harm 4·0 (3·3 - 4·8)	Ischemic heart disease 2·2 (1·1 - 2·9)	Colon and rectum cancer 1·9 (1·5 - 2·3)	Cervical cancer 1·7 (1·4 - 2·1)
40-44	Ischemic heart disease 14·1 (11·8 - 16·5)	Self-harm 6·9 (5·8 - 8·4)	Stroke 2·8 (2·1 - 3·5)	Cirrhosis and other chronic liver diseases 2·2 (1·8 - 2·6)	Colon and rectum cancer 2·2 (1·7 - 2·8)	Breast cancer 6·2 (5·2 - 7·7)	Self-harm 3·6 (2·9 - 4·2)	Ischemic heart disease 3·1 (1·5 - 4·0)	Stroke 2·5 (1·1 - 3·3)	Colon and rectum cancer 2·0 (1·6 - 2·4)
45-49	Ischemic heart disease 49·7 (42·9 - 56·9)	Self-harm 12·4 (10·4 - 15·0)	Stroke 10·9 (8·7 - 13·4)	Colon and rectum cancer 7·5 (6·1 - 8·9)	Tracheal, bronchus, and lung cancer 5·3 (3·4 - 7·7)	Breast cancer 15·1 (12·8 - 18·2)	Ischemic heart disease 7·6 (4·0 - 9·7)	Self-harm 5·1 (4·2 - 6·3)	Tracheal, bronchus, and lung cancer 4·6 (3·8 - 5·6)	Colon and rectum cancer 4·5 (3·7 - 5·5)
50-54	Ischemic heart disease 60·4 (52·6 - 69·0)	Colon and rectum cancer 11·3 (9·7 - 13·2)	Self-harm 9·8 (8·1 - 11·7)	Stroke 9·1 (7·2 - 11·2)	Lower respiratory infections 7·2 (5·1 - 9·4)	Breast cancer 26·0 (21·9 - 30·6)	Ischemic heart disease 14·0 (8·7 - 17·1)	Tracheal, bronchus, and lung cancer 9·4 (7·6 - 11·2)	Colon and rectum cancer 7·9 (6·5 - 9·4)	Self-harm 7·3 (6·0 - 8·8)
55-59	Ischemic heart disease 115·7 (101·3 - 132·3)	Colon and rectum cancer 22·1 (18·5 - 25·7)	Tracheal, bronchus, and lung cancer 20·6 (14·1 - 29·4)	Lower respiratory infections 16·7 (12·3 - 21·4)	Self-harm 15·1 (12·6 - 18·0)	Breast cancer 32·1 (27·4 - 37·3)	Ischemic heart disease 23·0 (15·8 - 27·8)	Colon and rectum cancer 15·0 (13·0 - 17·9)	Tracheal, bronchus, and lung cancer 14·7 (12·4 - 17·7)	Ovarian cancer 9·8 (8·3 - 11·5)
60-64	Ischemic heart disease 175·0 (150·7 - 196·8)	Lower respiratory infections 43·6 (32·3 - 56·8)	Colon and rectum cancer 41·4 (35·1 - 48·1)	Tracheal, bronchus, and lung cancer 39·6 (27·1 - 55·1)	Liver cancer 25·4 (21·8 - 29·2)	Ischemic heart disease 46·1 (33·5 - 53·4)	Breast cancer 41·2 (35·1 - 48·6)	Tracheal, bronchus, and lung cancer 28·6 (24·0 - 33·1)	Colon and rectum cancer 26·2 (22·8 - 30·3)	Lower respiratory infections 20·7 (15·3 - 25·2)
65-69	Ischemic heart disease 290·2 (254·3 - 331·7)	Lower respiratory infections 95·3 (70·8 - 123·3)	Tracheal, bronchus, and lung cancer 84·0 (58·3 - 116·2)	Colon and rectum cancer 72·6 (62·0 - 85·8)	Liver cancer 44·8 (38·5 - 51·5)	Ischemic heart disease 74·1 (55·8 - 87·2)	Breast cancer 54·0 (46·3 - 61·2)	Tracheal, bronchus, and lung cancer 46·7 (39·7 - 54·2)	Colon and rectum cancer 41·4 (35·9 - 47·7)	Lower respiratory infections 40·2 (31·3 - 47·7)

Age group (in years)	Males					Females				
	Top 1	Top 2	Top 3	Top 4	Top 5	Top 1	Top 2	Top 3	Top 4	Top 5
70-74	Ischemic heart disease 395·0 (340·3 - 447·9)	Lower respiratory infections 217·2 (170·0 - 267·8)	Tracheal, bronchus, and lung cancer 117·2 (79·1 - 163·5)	Colon and rectum cancer 94·3 (81·6 - 108·2)	Stroke 65·7 (54·1 - 77·9)	Ischemic heart disease 152·9 (123·0 - 176·7)	Lower respiratory infections 104·8 (79·7 - 123·8)	Tracheal, bronchus, and lung cancer 67·0 (57·5 - 76·6)	Breast cancer 65·6 (55·4 - 75·8)	Colon and rectum cancer 61·8 (53·8 - 70·8)
75-79	Ischemic heart disease 630·1 (534·8 - 730·9)	Lower respiratory infections 504·9 (394·7 - 626·4)	Tracheal, bronchus, and lung cancer 203·1 (135·9 - 284·5)	Colon and rectum cancer 147·0 (126·7 - 169·9)	Stroke 111·9 (90·4 - 133·2)	Ischemic heart disease 306·7 (244·8 - 360·3)	Lower respiratory infections 231·7 (178·9 - 278·7)	Alzheimer's disease and other dementias 127·1 (30·3 - 314·5)	Tracheal, bronchus, and lung cancer 105·8 (88·9 - 123·3)	Chronic kidney disease 104·2 (88·6 - 122·0)
80-84	Lower respiratory infections 1234·8 (971·8 - 1539·7)	Ischemic heart disease 1142·8 (934·9 - 1348·2)	Alzheimer's disease and other dementias 368·8 (85·9 - 927·4)	Tracheal, bronchus, and lung cancer 298·8 (193·2 - 420·8)	Colon and rectum cancer 221·2 (185·2 - 260·9)	Ischemic heart disease 578·1 (435·8 - 697·1)	Lower respiratory infections 574·9 (420·5 - 692·7)	Alzheimer's disease and other dementias 453·0 (115·9 - 1078·6)	Chronic kidney disease 200·5 (153·0 - 241·0)	Stroke 181·6 (132·1 - 226·4)
85-89	Lower respiratory infections 2015·6 (1556·0 - 2526·2)	Ischemic heart disease 1336·5 (1062·6 - 1596·9)	Alzheimer's disease and other dementias 720·1 (161·4 - 1899·3)	Chronic obstructive pulmonary disease 311·3 (187·3 - 399·3)	Tracheal, bronchus, and lung cancer 285·0 (178·2 - 404·2)	Lower respiratory infections 1287·9 (890·0 - 1565·4)	Alzheimer's disease and other dementias 1055·9 (285·3 - 2443·5)	Ischemic heart disease 993·6 (685·9 - 1240·0)	Stroke 313·8 (214·8 - 405·3)	Chronic kidney disease 259·1 (187·4 - 317·5)
90-94	Lower respiratory infections 3781·7 (2830·4 - 4755·4)	Ischemic heart disease 2174·9 (1697·6 - 2677·6)	Alzheimer's disease and other dementias 1340·6 (300·7 - 3532·2)	Chronic obstructive pulmonary disease 603·8 (360·8 - 772·7)	Chronic kidney disease 495·7 (375·2 - 596·0)	Lower respiratory infections 2621·9 (1861·4 - 3214·8)	Alzheimer's disease and other dementias 2054·9 (548·0 - 4705·3)	Ischemic heart disease 1759·0 (1173·9 - 2209·0)	Stroke 566·5 (361·5 - 728·2)	Urinary diseases and male infertility 528·9 (371·2 - 672·4)
95+	Lower respiratory infections 6650·2 (4977·6 - 8376·7)	Ischemic heart disease 3463·8 (2556·9 - 4328·5)	Alzheimer's disease and other dementias 2575·1 (584·9 - 6331·4)	Chronic obstructive pulmonary disease 1279·1 (771·0 - 1628·4)	Chronic kidney disease 931·8 (691·2 - 1126·3)	Lower respiratory infections 5167·5 (3444·0 - 6487·7)	Alzheimer's disease and other dementias 4039·6 (1007·8 - 9175·3)	Ischemic heart disease 3002·2 (1885·0 - 3780·5)	Urinary diseases and male infertility 1161·8 (758·6 - 1500·6)	Stroke 1076·5 (664·0 - 1428·7)

Table S14 | Mortality rates (per 100 000) for the top five Level 3 causes of death by sex and age groups in Thailand, 2023

Age group (in years)	Males: Top 1	Males: Top 2	Males: Top 3	Males: Top 4	Males: Top 5	Females: Top 1	Females: Top 2	Females: Top 3	Females: Top 4	Females: Top 5
<1	Neonatal disorders 276·2 (227·3 - 323·3)	Congenital birth defects 152·0 (103·4 - 210·5)	Lower respiratory infections 61·4 (47·1 - 78·4)	COVID-19 21·4 (16·2 - 27·8)	Diarrheal diseases 20·3 (9·2 - 36·1)	Neonatal disorders 206·7 (168·2 - 246·0)	Congenital birth defects 141·2 (97·9 - 185·7)	Lower respiratory infections 47·0 (36·3 - 60·2)	Diarrheal diseases 17·7 (6·9 - 37·4)	COVID-19 14·1 (10·3 - 18·4)
2-4	Drowning 10·4 (6·0 - 15·7)	Congenital birth defects 5·7 (3·8 - 8·3)	Road injuries 4·7 (2·6 - 7·2)	Lower respiratory infections 4·6 (2·7 - 7·2)	Leukemia 3·0 (1·8 - 5·3)	Congenital birth defects 4·2 (2·7 - 5·9)	Drowning 3·9 (2·1 - 6·0)	Lower respiratory infections 3·8 (2·4 - 5·7)	Road injuries 3·4 (1·7 - 5·5)	Measles 2·6 (1·0 - 4·9)
5-9	Drowning 8·4 (4·9 - 12·6)	Road injuries 4·9 (3·1 - 7·2)	Lower respiratory infections 3·5 (1·9 - 5·2)	Congenital birth defects 2·7 (1·9 - 3·6)	Leukemia 2·5 (1·7 - 3·7)	Drowning 3·7 (2·1 - 5·9)	Road injuries 3·5 (2·0 - 5·3)	Lower respiratory infections 2·7 (1·7 - 4·0)	Congenital birth defects 2·4 (1·6 - 3·2)	Leukemia 1·7 (1·2 - 2·4)
10-14	Road injuries 16·0 (6·7 - 23·2)	Drowning 7·5 (4·9 - 12·0)	Lower respiratory infections 2·9 (1·8 - 4·4)	Leukemia 2·8 (1·8 - 4·6)	Congenital birth defects 2·6 (1·8 - 3·7)	Road injuries 6·1 (2·5 - 9·6)	Drowning 3·9 (2·4 - 6·0)	Congenital birth defects 2·0 (1·4 - 2·9)	Leukemia 1·8 (1·2 - 2·6)	Lower respiratory infections 1·8 (1·2 - 2·6)
15-19	Road injuries 49·0 (25·0 - 67·1)	Self-harm 11·3 (7·3 - 16·6)	Interpersonal violence 10·6 (5·6 - 19·5)	Drowning 6·0 (3·4 - 10·9)	HIV/AIDS 4·2 (0·0 - 10·5)	Road injuries 13·9 (5·8 - 20·4)	HIV/AIDS 3·3 (0·0 - 8·0)	Self-harm 3·0 (1·9 - 4·8)	Other musculoskeletal disorders 2·3 (1·5 - 3·5)	Leukemia 1·9 (1·3 - 2·8)
20-24	Road injuries 64·7 (35·5 - 85·0)	Self-harm 21·4 (14·8 - 29·4)	Interpersonal violence 16·4 (9·6 - 25·5)	HIV/AIDS 14·1 (0·0 - 32·8)	Drowning 6·2 (3·9 - 9·4)	Road injuries 13·2 (5·9 - 18·7)	HIV/AIDS 6·1 (0·0 - 15·4)	Self-harm 3·4 (2·1 - 5·2)	Other musculoskeletal disorders 3·1 (1·8 - 4·6)	Chronic kidney disease 2·5 (1·5 - 3·9)
25-29	Road injuries 63·2 (35·6 - 86·1)	HIV/AIDS 31·1 (0·0 - 68·9)	Self-harm 30·8 (20·7 - 43·1)	Interpersonal violence 19·3 (11·5 - 29·0)	Stroke 8·0 (4·9 - 13·6)	Road injuries 11·6 (5·1 - 17·8)	HIV/AIDS 10·5 (0·0 - 23·2)	Self-harm 4·3 (2·7 - 6·2)	Chronic kidney disease 3·5 (2·2 - 5·3)	Other musculoskeletal disorders 3·4 (1·9 - 5·2)
30-34	Road injuries 65·7 (34·4 - 90·7)	HIV/AIDS 45·6 (0·0 - 105·2)	Self-harm 36·0 (19·9 - 49·4)	Interpersonal violence 19·9 (10·6 - 29·9)	Stroke 19·9 (12·8 - 30·3)	HIV/AIDS 12·8 (0·0 - 30·1)	Road injuries 11·4 (5·4 - 17·1)	Breast cancer 6·5 (4·7 - 9·0)	Chronic kidney disease 5·0 (3·3 - 7·2)	Self-harm 4·6 (2·9 - 6·6)

Age group (in years)	Males					Females				
	Top 1	Top 2	Top 3	Top 4	Top 5	Top 1	Top 2	Top 3	Top 4	Top 5
35-39	Road injuries 63·4 (37·4 - 86·4)	HIV/AIDS 51·3 (0·0 - 112·1)	Stroke 38·1 (26·2 - 55·7)	Cirrhosis and other chronic liver diseases 37·2 (24·9 - 53·0)	Self-harm 32·8 (20·0 - 45·9)	HIV/AIDS 18·1 (0·0 - 41·4)	Breast cancer 12·7 (8·8 - 17·0)	Road injuries 10·5 (5·7 - 14·8)	Stroke 7·7 (4·8 - 11·4)	Cervical cancer 7·1 (4·3 - 11·1)
40-44	Road injuries 62·5 (37·8 - 82·3)	Stroke 59·4 (42·2 - 83·5)	Cirrhosis and other chronic liver diseases 59·0 (42·2 - 77·3)	HIV/AIDS 54·8 (0·0 - 126·3)	Ischemic heart disease 41·4 (28·5 - 60·6)	Breast cancer 22·2 (16·8 - 30·0)	HIV/AIDS 17·7 (0·0 - 42·9)	Stroke 13·8 (8·8 - 18·7)	Cervical cancer 12·3 (8·3 - 20·7)	Road injuries 11·7 (6·7 - 16·1)
45-49	Stroke 79·6 (57·4 - 110·2)	HIV/AIDS 74·4 (0·0 - 164·6)	Cirrhosis and other chronic liver diseases 68·7 (51·6 - 90·9)	Road injuries 58·4 (35·9 - 77·6)	Ischemic heart disease 57·6 (40·7 - 81·4)	Breast cancer 32·2 (24·5 - 41·7)	Stroke 23·2 (16·6 - 30·8)	HIV/AIDS 18·5 (0·0 - 46·7)	Cervical cancer 15·9 (10·5 - 26·7)	Chronic kidney disease 15·9 (11·4 - 21·7)
50-54	Stroke 103·0 (78·9 - 133·8)	Ischemic heart disease 80·4 (59·7 - 103·3)	Cirrhosis and other chronic liver diseases 77·2 (56·2 - 99·9)	HIV/AIDS 61·2 (0·0 - 135·0)	Road injuries 51·7 (35·7 - 71·2)	Breast cancer 41·2 (32·1 - 51·8)	Stroke 34·7 (25·1 - 45·1)	Chronic kidney disease 24·8 (17·7 - 33·8)	Cervical cancer 20·6 (13·0 - 35·0)	Diabetes mellitus 20·0 (13·6 - 29·2)
55-59	Stroke 125·7 (93·8 - 161·3)	Ischemic heart disease 100·2 (73·1 - 130·6)	Cirrhosis and other chronic liver diseases 79·5 (56·5 - 103·1)	Tracheal, bronchus, and lung cancer 73·7 (53·7 - 108·4)	Liver cancer 54·2 (36·7 - 85·4)	Breast cancer 48·6 (37·8 - 61·8)	Stroke 47·2 (35·2 - 59·0)	Chronic kidney disease 38·0 (26·4 - 50·9)	Diabetes mellitus 34·4 (22·1 - 52·1)	Ischemic heart disease 29·2 (19·3 - 41·0)
60-64	Stroke 157·7 (119·3 - 202·2)	Ischemic heart disease 127·1 (94·0 - 164·4)	Tracheal, bronchus, and lung cancer 111·3 (82·3 - 173·5)	Cirrhosis and other chronic liver diseases 78·7 (57·4 - 105·8)	Lower respiratory infections 67·9 (41·8 - 98·3)	Stroke 65·4 (50·0 - 83·4)	Diabetes mellitus 60·7 (41·3 - 88·5)	Chronic kidney disease 53·2 (39·0 - 70·1)	Ischemic heart disease 44·5 (33·1 - 58·4)	Breast cancer 44·3 (33·3 - 57·2)
65-69	Stroke 215·9 (166·1 - 285·3)	Ischemic heart disease 170·5 (128·1 - 226·5)	Tracheal, bronchus, and lung cancer 159·6 (121·9 - 237·0)	Lower respiratory infections 127·2 (78·2 - 183·1)	Diabetes mellitus 101·2 (74·2 - 134·7)	Stroke 112·3 (87·0 - 147·6)	Diabetes mellitus 103·0 (69·1 - 145·8)	Chronic kidney disease 89·2 (63·0 - 118·0)	Ischemic heart disease 77·3 (55·9 - 102·3)	Tracheal, bronchus, and lung cancer 60·1 (43·8 - 86·4)
70-74	Stroke 334·9 (250·2 - 429·9)	Lower respiratory infections 273·1 (162·5 - 395·6)	Ischemic heart disease 252·4 (185·5 - 337·1)	Tracheal, bronchus, and lung cancer	Diabetes mellitus 141·7 (100·0 - 189·8)	Stroke 215·4 (165·9 - 275·6)	Diabetes mellitus 162·5 (112·5 - 226·1)	Lower respiratory infections 148·9 (92·0 - 215·8)	Chronic kidney disease 148·3 (98·2 - 202·2)	Ischemic heart disease 142·5 (102·4 - 193·3)

Age group (in years)	Males					Females				
	Top 1	Top 2	Top 3	Top 4	Top 5	Top 1	Top 2	Top 3	Top 4	Top 5
				229·9 (174·7 - 332·3)						
75-79	Lower respiratory infections 466·5 (297·5 - 660·5)	Stroke 460·7 (332·2 - 579·5)	Ischemic heart disease 334·8 (252·4 - 441·1)	Tracheal, bronchus, and lung cancer 286·2 (217·9 - 405·4)	Chronic obstructive pulmonary disease 280·5 (204·0 - 364·0)	Stroke 362·2 (280·8 - 462·3)	Lower respiratory infections 276·2 (166·5 - 392·6)	Ischemic heart disease 240·2 (168·3 - 313·4)	Diabetes mellitus 220·6 (141·4 - 313·9)	Chronic kidney disease 208·0 (136·7 - 284·2)
80-84	Lower respiratory infections 820·6 (528·3 - 1148·8)	Stroke 734·7 (529·2 - 961·1)	Ischemic heart disease 549·8 (402·2 - 734·1)	Chronic obstructive pulmonary disease 538·5 (390·1 - 696·1)	Tracheal, bronchus, and lung cancer 373·7 (260·6 - 524·9)	Stroke 712·4 (497·1 - 967·0)	Lower respiratory infections 538·8 (341·2 - 788·2)	Ischemic heart disease 498·8 (353·6 - 670·3)	Alzheimer's disease and other dementias 487·6 (115·6 - 1202·7)	Chronic kidney disease 348·2 (228·5 - 486·9)
85-89	Lower respiratory infections 1223·8 (807·0 - 1741·8)	Other neurological disorders 916·7 (436·5 - 1867·4)	Stroke 852·1 (590·7 - 1165·0)	Chronic obstructive pulmonary disease 728·0 (520·6 - 1000·3)	Ischemic heart disease 696·8 (478·4 - 970·2)	Other neurological disorders 1264·6 (563·0 - 2366·0)	Stroke 908·7 (584·0 - 1321·0)	Alzheimer's disease and other dementias 875·3 (225·2 - 2203·0)	Lower respiratory infections 844·6 (520·3 - 1265·3)	Ischemic heart disease 630·2 (393·6 - 892·1)
90-94	Other neurological disorders 1726·6 (967·9 - 2824·0)	Lower respiratory infections 1610·8 (1049·0 - 2291·2)	Alzheimer's disease and other dementias 1252·2 (288·1 - 3415·1)	Chronic obstructive pulmonary disease 949·6 (600·5 - 1312·0)	Stroke 721·2 (457·7 - 1019·6)	Other neurological disorders 2066·0 (1030·8 - 3619·3)	Alzheimer's disease and other dementias 1781·9 (440·8 - 4222·2)	Lower respiratory infections 1452·1 (875·9 - 2141·0)	Stroke 997·7 (554·7 - 1590·6)	Ischemic heart disease 756·0 (500·8 - 1119·8)
95+	Lower respiratory infections 2847·2 (1669·6 - 4174·9)	Alzheimer's disease and other dementias 2547·5 (572·4 - 6494·3)	Other neurological disorders 1878·6 (844·4 - 3480·7)	Chronic obstructive pulmonary disease 1535·1 (939·2 - 2265·2)	Stroke 1136·4 (637·2 - 1743·2)	Alzheimer's disease and other dementias 3477·5 (806·3 - 8580·2)	Other neurological disorders 3307·7 (1624·8 - 5684·6)	Lower respiratory infections 2619·4 (1346·8 - 4261·2)	Stroke 1588·9 (818·9 - 2743·1)	Ischemic heart disease 1315·4 (751·3 - 2165·0)

Table S15 | Mortality rates (per 100 000) for the top five Level 3 causes of death by sex and age groups in Timor-Leste, 2023

Age group (in years)	Males					Females				
	Top 1	Top 2	Top 3	Top 4	Top 5	Top 1	Top 2	Top 3	Top 4	Top 5
<1	Neonatal disorders 1618·2 (1237·8 - 2068·8)	Lower respiratory infections 521·2 (294·2 - 849·4)	Congenital birth defects 349·6 (185·9 - 581·3)	Diarrheal diseases 75·5 (22·8 - 178·6)	COVID-19 68·5 (42·5 - 105·5)	Neonatal disorders 1100·8 (836·6 - 1409·8)	Lower respiratory infections 508·1 (295·9 - 728·1)	Congenital birth defects 377·3 (209·9 - 631·8)	Protein-energy malnutrition 78·0 (49·2 - 114·6)	Pertussis 59·3 (6·6 - 167·3)
2-4	Lower respiratory infections 46·2 (23·5 - 74·3)	Drowning 14·1 (6·2 - 27·3)	Congenital birth defects 12·8 (7·0 - 21·8)	Protein-energy malnutrition 9·8 (3·6 - 19·1)	Meningitis 9·7 (3·9 - 18·9)	Lower respiratory infections 56·2 (32·3 - 91·8)	Congenital birth defects 13·3 (6·6 - 24·6)	Pertussis 10·6 (1·2 - 31·5)	Protein-energy malnutrition 9·9 (5·9 - 14·9)	Meningitis 9·0 (3·5 - 22·6)
5-9	Drowning 14·2 (6·9 - 24·7)	Lower respiratory infections 9·6 (5·5 - 15·4)	Road injuries 7·1 (3·7 - 12·2)	Congenital birth defects 5·5 (3·3 - 9·7)	Tuberculosis 4·1 (1·9 - 8·2)	Lower respiratory infections 9·4 (5·0 - 15·4)	Drowning 8·4 (3·7 - 15·5)	Congenital birth defects 6·0 (3·3 - 10·8)	Tuberculosis 5·0 (2·2 - 9·7)	Road injuries 4·7 (2·1 - 8·8)
10-14	Drowning 8·4 (4·4 - 14·1)	Road injuries 7·2 (4·2 - 11·8)	Lower respiratory infections 5·0 (3·1 - 8·0)	Congenital birth defects 3·7 (2·2 - 6·1)	Tuberculosis 3·6 (1·7 - 7·4)	Drowning 10·4 (5·1 - 17·8)	Tuberculosis 6·9 (2·8 - 12·8)	Road injuries 6·6 (3·0 - 11·4)	Lower respiratory infections 6·1 (3·4 - 10·2)	Congenital birth defects 5·6 (3·0 - 9·8)
15-19	Road injuries 21·6 (13·1 - 34·1)	Drowning 7·0 (3·6 - 12·3)	Tuberculosis 6·6 (3·0 - 12·7)	Interpersonal violence 6·2 (2·5 - 14·1)	Self-harm 5·7 (3·0 - 9·2)	Maternal disorders 18·5 (8·9 - 30·2)	Road injuries 14·2 (6·7 - 25·3)	Tuberculosis 12·5 (4·8 - 23·6)	Rheumatic heart disease 6·0 (2·3 - 10·7)	Lower respiratory infections 5·6 (2·7 - 10·7)
20-24	Road injuries 35·0 (20·7 - 55·1)	Tuberculosis 11·6 (5·8 - 20·8)	Self-harm 9·0 (4·8 - 14·5)	Interpersonal violence 8·7 (3·8 - 17·4)	Drowning 7·3 (3·8 - 12·8)	Maternal disorders 43·0 (22·7 - 65·3)	Tuberculosis 18·7 (7·0 - 34·5)	Road injuries 15·5 (8·0 - 26·6)	Rheumatic heart disease 7·3 (3·0 - 12·8)	Stroke 6·6 (3·5 - 11·7)
25-29	Road injuries 39·6 (24·8 - 60·3)	Tuberculosis 19·3 (9·9 - 34·5)	Ischemic heart disease 11·9 (5·9 - 20·0)	HIV/AIDS 11·3 (4·7 - 23·3)	Self-harm 9·9 (5·4 - 16·6)	Maternal disorders 61·3 (30·4 - 102·4)	Tuberculosis 25·8 (9·9 - 49·2)	Road injuries 21·7 (10·4 - 35·7)	HIV/AIDS 18·0 (7·6 - 34·9)	Stroke 16·4 (8·9 - 31·0)
30-34	Road injuries 41·8 (26·8 - 62·7)	Ischemic heart disease 25·4 (12·8 - 41·5)	Tuberculosis 25·3 (13·1 - 46·4)	Stroke 20·0 (8·9 - 36·2)	HIV/AIDS 19·9 (10·2 - 35·5)	Maternal disorders 79·6 (44·7 - 130·0)	Stroke 39·0 (21·5 - 64·3)	HIV/AIDS 30·6 (16·0 - 51·7)	Tuberculosis 29·6 (12·7 - 57·0)	Ischemic heart disease 28·5 (13·4 - 49·6)
35-39	Ischemic heart disease 33·2 (18·3 - 56·0)	Road injuries 32·1 (19·4 - 49·7)	Tuberculosis 31·0 (17·1 - 56·6)	Stroke 30·3 (15·2 - 50·4)	HIV/AIDS 22·2 (12·0 - 36·0)	Maternal disorders 61·1 (32·7 - 98·1)	Stroke 33·4 (19·6 - 60·7)	HIV/AIDS 29·5 (15·6 - 49·8)	Tuberculosis 27·1 (11·2 - 51·7)	Ischemic heart disease 20·5 (9·0 - 38·7)

Age group (in years)	Males					Females				
	Top 1	Top 2	Top 3	Top 4	Top 5	Top 1	Top 2	Top 3	Top 4	Top 5
40-44	Ischemic heart disease 55·0 (33·4 - 86·4)	Stroke 53·7 (25·9 - 93·3)	Tuberculosis 41·7 (23·0 - 71·8)	Road injuries 31·7 (19·5 - 49·0)	Cirrhosis and other chronic liver diseases 29·6 (16·7 - 45·7)	Stroke 59·1 (33·2 - 89·9)	Maternal disorders 56·5 (27·6 - 93·8)	Tuberculosis 34·5 (15·0 - 60·5)	Ischemic heart disease 33·2 (15·8 - 60·8)	HIV/AIDS 32·6 (20·1 - 49·5)
45-49	Stroke 64·8 (33·6 - 108·8)	Ischemic heart disease 63·3 (38·0 - 103·8)	Tuberculosis 54·1 (30·0 - 95·4)	Cirrhosis and other chronic liver diseases 38·9 (22·7 - 61·0)	Road injuries 23·5 (13·8 - 36·4)	Stroke 63·5 (39·7 - 99·6)	Tuberculosis 41·9 (17·1 - 76·8)	Ischemic heart disease 32·6 (15·3 - 58·9)	HIV/AIDS 27·6 (15·9 - 44·2)	Breast cancer 26·6 (16·0 - 40·4)
50-54	Stroke 115·2 (60·3 - 185·3)	Ischemic heart disease 108·4 (65·5 - 182·4)	Tuberculosis 80·5 (44·2 - 154·6)	Cirrhosis and other chronic liver diseases 54·3 (33·9 - 89·2)	Chronic kidney disease 29·0 (16·5 - 48·4)	Stroke 107·8 (68·0 - 166·0)	Tuberculosis 62·0 (22·7 - 127·8)	Ischemic heart disease 55·0 (27·5 - 96·0)	Breast cancer 36·6 (21·1 - 53·2)	Diabetes mellitus 33·3 (18·1 - 54·8)
55-59	Stroke 197·2 (105·7 - 319·2)	Ischemic heart disease 186·6 (111·4 - 299·1)	Tuberculosis 120·7 (62·8 - 243·2)	Cirrhosis and other chronic liver diseases 73·5 (41·7 - 117·7)	Chronic obstructive pulmonary disease 55·6 (32·1 - 88·4)	Stroke 179·3 (112·3 - 289·8)	Ischemic heart disease 98·5 (45·5 - 179·1)	Tuberculosis 89·5 (35·5 - 189·1)	Diabetes mellitus 63·0 (34·7 - 101·6)	Breast cancer 48·6 (29·1 - 71·7)
60-64	Stroke 330·6 (196·2 - 528·4)	Ischemic heart disease 287·0 (172·0 - 463·6)	Tuberculosis 198·7 (105·4 - 423·6)	Chronic obstructive pulmonary disease 132·0 (77·5 - 204·9)	Cirrhosis and other chronic liver diseases 92·2 (52·9 - 150·0)	Stroke 315·3 (198·1 - 519·8)	Ischemic heart disease 181·3 (85·8 - 308·3)	Tuberculosis 141·4 (53·1 - 349·6)	Diabetes mellitus 110·3 (62·8 - 175·6)	Lower respiratory infections 79·4 (39·8 - 160·2)
65-69	Stroke 548·7 (318·2 - 837·5)	Ischemic heart disease 456·5 (255·8 - 726·0)	Tuberculosis 301·9 (144·3 - 699·2)	Chronic obstructive pulmonary disease 266·0 (153·4 - 411·8)	Lower respiratory infections 161·1 (81·4 - 282·5)	Stroke 553·9 (354·9 - 858·6)	Ischemic heart disease 320·4 (153·8 - 594·5)	Tuberculosis 203·3 (69·8 - 476·4)	Diabetes mellitus 169·7 (93·0 - 291·0)	Lower respiratory infections 139·0 (69·9 - 270·0)
70-74	Stroke 888·5 (522·2 - 1355·0)	Ischemic heart disease 685·8 (381·5 - 1101·5)	Chronic obstructive pulmonary disease 508·8 (288·9 - 808·7)	Tuberculosis 428·9 (189·2 - 1076·0)	Lower respiratory infections 318·9 (167·0 - 582·3)	Stroke 999·2 (638·7 - 1433·7)	Ischemic heart disease 549·9 (270·9 - 969·6)	Lower respiratory infections 292·6 (140·0 - 556·1)	Tuberculosis 283·6 (101·8 - 648·6)	Hypertensive heart disease 253·5 (69·4 - 633·9)

Age group (in years)	Males					Females				
	Top 1	Top 2	Top 3	Top 4	Top 5	Top 1	Top 2	Top 3	Top 4	Top 5
75-79	Stroke 1360·4 (723·4 - 2054·9)	Ischemic heart disease 1032·2 (596·1 - 1700·3)	Chronic obstructive pulmonary disease 852·8 (481·0 - 1340·0)	Tuberculosis 580·2 (268·7 - 1475·5)	Lower respiratory infections 565·9 (311·8 - 992·9)	Stroke 1688·3 (1124·3 - 2450·5)	Ischemic heart disease 913·4 (458·5 - 1582·5)	Lower respiratory infections 538·6 (283·4 - 1011·7)	Hypertensive heart disease 426·3 (112·0 - 1093·9)	Tuberculosis 373·7 (150·6 - 843·2)
80-84	Stroke 1799·8 (981·7 - 2830·8)	Ischemic heart disease 1499·1 (895·3 - 2420·5)	Chronic obstructive pulmonary disease 1332·8 (771·6 - 2114·7)	Lower respiratory infections 1088·9 (616·8 - 1818·0)	Tuberculosis 718·2 (334·5 - 1772·6)	Stroke 2699·2 (1741·8 - 3841·1)	Ischemic heart disease 1616·0 (771·7 - 2878·6)	Lower respiratory infections 1124·2 (585·6 - 2125·4)	Hypertensive heart disease 708·6 (192·8 - 1721·1)	Alzheimer's disease and other dementias 532·8 (116·6 - 1638·4)
85-89	Stroke 2136·0 (1173·6 - 3374·2)	Ischemic heart disease 1939·3 (1062·2 - 3088·3)	Chronic obstructive pulmonary disease 1885·9 (1102·1 - 3022·9)	Lower respiratory infections 1764·0 (1018·5 - 3074·9)	Tuberculosis 800·2 (379·5 - 1903·5)	Stroke 4044·0 (2509·9 - 6349·6)	Ischemic heart disease 2729·1 (1345·6 - 4362·9)	Lower respiratory infections 1981·0 (1048·0 - 3572·6)	Hypertensive heart disease 1124·3 (331·7 - 2753·7)	Alzheimer's disease and other dementias 1113·1 (246·5 - 3310·5)
90-94	Lower respiratory infections 2810·1 (1706·4 - 4471·1)	Ischemic heart disease 2393·7 (1376·6 - 4014·5)	Chronic obstructive pulmonary disease 2171·8 (1248·6 - 3378·7)	Stroke 2081·1 (1072·0 - 3291·4)	Alzheimer's disease and other dementias 1268·1 (238·6 - 3671·8)	Stroke 4892·9 (2976·7 - 7774·3)	Ischemic heart disease 4059·5 (2082·4 - 7166·1)	Lower respiratory infections 3201·6 (1626·4 - 5552·8)	Alzheimer's disease and other dementias 1942·2 (436·6 - 5772·0)	Hypertensive heart disease 1442·8 (404·4 - 3677·3)
95+	Lower respiratory infections 4104·0 (2169·5 - 6941·1)	Ischemic heart disease 3096·7 (1633·5 - 5136·6)	Chronic obstructive pulmonary disease 2516·2 (1352·3 - 3940·4)	Stroke 2311·1 (1063·6 - 4239·0)	Alzheimer's disease and other dementias 2209·5 (472·4 - 5737·7)	Stroke 6467·4 (3621·1 - 10642·8)	Ischemic heart disease 6280·4 (2921·1 - 10732·5)	Lower respiratory infections 4580·8 (2186·3 - 8655·8)	Alzheimer's disease and other dementias 3037·6 (601·4 - 8616·3)	Hypertensive heart disease 1882·8 (475·1 - 4676·1)

Table S16 | Mortality rates (per 100 000) for the top five Level 3 causes of death by sex and age groups in Viet Nam, 2023

Age group (in years)	Males					Females				
	Top 1	Top 2	Top 3	Top 4	Top 5	Top 1	Top 2	Top 3	Top 4	Top 5
<1	Neonatal disorders 403·9 (275·0 - 555·4)	Congenital birth defects 277·2 (169·4 - 401·0)	Lower respiratory infections 153·5 (95·7 - 219·4)	COVID-19 28·8 (21·2 - 39·0)	Sexually transmitted infections excluding HIV 22·6 (7·7 - 46·3)	Neonatal disorders 282·4 (196·1 - 384·0)	Congenital birth defects 223·3 (129·2 - 311·3)	Lower respiratory infections 58·2 (35·1 - 87·8)	Other unspecified infectious diseases 25·7 (9·0 - 63·3)	COVID-19 14·3 (10·1 - 19·3)
2-4	Drowning 46·9 (25·1 - 74·4)	Lower respiratory infections 6·8 (3·2 - 12·1)	Congenital birth defects 5·8 (3·1 - 10·3)	Measles 5·8 (2·0 - 12·5)	Encephalitis 4·3 (1·1 - 9·9)	Drowning 8·0 (4·2 - 13·8)	Congenital birth defects 4·4 (2·3 - 7·3)	Measles 3·4 (1·2 - 6·6)	Lower respiratory infections 3·3 (1·6 - 5·4)	Pertussis 1·9 (0·2 - 5·5)
5-9	Drowning 20·5 (12·2 - 30·9)	Road injuries 4·4 (2·0 - 9·4)	Lower respiratory infections 2·5 (1·2 - 4·7)	Congenital birth defects 1·6 (0·9 - 2·6)	Falls 1·6 (0·7 - 2·9)	Drowning 7·2 (3·8 - 11·7)	Road injuries 2·2 (1·1 - 4·3)	Congenital birth defects 1·9 (1·1 - 3·2)	Lower respiratory infections 1·5 (0·7 - 2·5)	Leukemia 1·1 (0·7 - 1·7)
10-14	Drowning 12·8 (7·6 - 19·7)	Road injuries 6·3 (3·1 - 11·4)	Congenital birth defects 1·6 (0·9 - 2·6)	Falls 1·5 (0·7 - 2·6)	Leukemia 1·5 (0·9 - 2·4)	Drowning 9·3 (5·5 - 14·6)	Road injuries 4·1 (2·1 - 6·9)	Congenital birth defects 2·1 (1·3 - 3·3)	Leukemia 1·3 (0·8 - 2·1)	Lower respiratory infections 1·0 (0·5 - 1·6)
15-19	Road injuries 18·1 (9·3 - 32·8)	Drowning 7·5 (3·7 - 13·6)	Self-harm 5·2 (2·8 - 8·9)	Interpersonal violence 3·1 (1·4 - 6·6)	HIV/AIDS 2·7 (0·0 - 7·1)	Road injuries 11·8 (6·0 - 19·3)	Self-harm 5·9 (3·0 - 10·7)	Drowning 3·7 (1·9 - 6·4)	Maternal disorders 3·1 (1·5 - 5·5)	Congenital birth defects 1·9 (1·0 - 2·9)
20-24	Road injuries 36·8 (21·4 - 61·0)	Self-harm 12·2 (6·8 - 19·7)	HIV/AIDS 10·3 (0·0 - 26·4)	Drowning 8·9 (4·1 - 16·5)	Interpersonal violence 5·3 (2·6 - 11·8)	Road injuries 14·2 (7·3 - 23·2)	Self-harm 7·5 (3·8 - 11·8)	Maternal disorders 6·5 (3·5 - 10·8)	HIV/AIDS 4·0 (0·0 - 10·6)	Chronic kidney disease 2·8 (0·9 - 5·7)
25-29	Road injuries 32·3 (17·1 - 54·9)	HIV/AIDS 24·3 (0·0 - 55·5)	Self-harm 13·0 (6·9 - 22·8)	Drowning 7·3 (3·5 - 14·6)	Falls 6·0 (2·8 - 11·0)	Road injuries 15·6 (7·3 - 26·1)	HIV/AIDS 8·7 (0·0 - 24·4)	Self-harm 7·7 (3·9 - 12·4)	Maternal disorders 7·4 (4·0 - 12·3)	Chronic kidney disease 4·2 (1·3 - 7·7)
30-34	Road injuries 37·5 (18·4 - 74·6)	HIV/AIDS 34·3 (0·0 - 82·4)	Self-harm 17·8 (9·3 - 29·4)	Stroke 10·6 (5·5 - 17·2)	Cirrhosis and other chronic liver diseases 9·3 (5·1 - 14·6)	Road injuries 14·8 (7·1 - 26·7)	HIV/AIDS 9·1 (0·0 - 23·9)	Maternal disorders 7·6 (3·8 - 12·7)	Self-harm 6·8 (3·6 - 10·6)	Breast cancer 5·1 (2·9 - 7·8)

Age group (in years)	Males					Females				
	Top 1	Top 2	Top 3	Top 4	Top 5	Top 1	Top 2	Top 3	Top 4	Top 5
35-39	Road injuries 35·9 (18·0 - 63·9)	HIV/AIDS 34·3 (0·0 - 82·3)	Stroke 23·9 (13·1 - 39·0)	Cirrhosis and other chronic liver diseases 21·7 (13·1 - 32·9)	Self-harm 19·1 (8·8 - 34·0)	Road injuries 20·5 (8·8 - 35·4)	Breast cancer 15·3 (9·2 - 24·4)	HIV/AIDS 9·0 (0·0 - 23·8)	Stroke 8·9 (4·5 - 16·8)	Cervical cancer 8·8 (4·5 - 18·4)
40-44	Stroke 54·5 (33·1 - 83·1)	Cirrhosis and other chronic liver diseases 46·8 (28·1 - 72·9)	Road injuries 43·8 (21·0 - 82·6)	HIV/AIDS 29·4 (0·0 - 75·0)	Ischemic heart disease 28·9 (15·2 - 50·7)	Breast cancer 22·0 (14·0 - 35·3)	Road injuries 21·4 (8·9 - 38·3)	Stroke 15·7 (8·8 - 29·3)	Cervical cancer 13·8 (7·9 - 25·5)	Ischemic heart disease 11·5 (5·9 - 18·0)
45-49	Stroke 96·2 (58·0 - 143·2)	Cirrhosis and other chronic liver diseases 70·3 (42·3 - 109·9)	Road injuries 50·0 (22·8 - 100·1)	Ischemic heart disease 42·8 (23·8 - 68·7)	Tracheal, bronchus, and lung cancer 30·3 (16·1 - 47·1)	Stroke 31·0 (18·9 - 50·7)	Breast cancer 30·7 (18·7 - 45·9)	Road injuries 24·5 (10·7 - 41·9)	Ischemic heart disease 21·8 (12·1 - 33·9)	Cervical cancer 18·8 (10·5 - 30·6)
50-54	Stroke 186·8 (121·4 - 276·3)	Cirrhosis and other chronic liver diseases 97·7 (57·9 - 146·1)	Ischemic heart disease 80·4 (45·6 - 125·9)	Tracheal, bronchus, and lung cancer 67·8 (37·0 - 106·7)	Road injuries 58·2 (27·5 - 113·8)	Stroke 49·2 (31·5 - 79·1)	Ischemic heart disease 33·0 (18·4 - 52·5)	Breast cancer 32·9 (20·5 - 48·9)	Road injuries 26·5 (9·8 - 48·3)	Cervical cancer 22·9 (12·6 - 39·0)
55-59	Stroke 235·6 (162·7 - 341·5)	Ischemic heart disease 97·5 (54·9 - 155·2)	Cirrhosis and other chronic liver diseases 88·4 (53·8 - 134·0)	Tracheal, bronchus, and lung cancer 83·1 (45·0 - 126·9)	Road injuries 45·2 (21·7 - 91·6)	Stroke 74·3 (49·6 - 111·2)	Ischemic heart disease 48·9 (28·5 - 78·5)	Breast cancer 39·5 (24·6 - 59·4)	Diabetes mellitus 37·4 (22·3 - 57·8)	Tracheal, bronchus, and lung cancer 33·7 (19·6 - 52·6)
60-64	Stroke 496·7 (330·1 - 684·9)	Ischemic heart disease 214·6 (132·5 - 332·9)	Tracheal, bronchus, and lung cancer 178·2 (90·0 - 274·5)	Chronic obstructive pulmonary disease 91·4 (55·8 - 136·2)	Cirrhosis and other chronic liver diseases 90·7 (50·8 - 142·4)	Stroke 156·4 (96·1 - 243·5)	Ischemic heart disease 82·6 (46·5 - 122·5)	Diabetes mellitus 76·2 (42·6 - 121·8)	Tracheal, bronchus, and lung cancer 65·0 (39·6 - 97·8)	Breast cancer 48·2 (31·0 - 76·6)
65-69	Stroke 614·6 (416·4 - 855·2)	Ischemic heart disease 268·1 (163·7 - 428·0)	Tracheal, bronchus, and lung cancer 186·3 (97·6 - 281·1)	Chronic obstructive pulmonary disease 161·1 (101·1 - 244·9)	Diabetes mellitus 122·6 (72·0 - 191·5)	Stroke 245·7 (163·5 - 357·6)	Diabetes mellitus 117·3 (63·7 - 203·9)	Ischemic heart disease 107·0 (58·9 - 174·6)	Chronic obstructive pulmonary disease 68·5 (31·3 - 115·6)	Hypertensive heart disease 64·5 (29·9 - 115·7)

Age group (in years)	Males					Females				
	Top 1	Top 2	Top 3	Top 4	Top 5	Top 1	Top 2	Top 3	Top 4	Top 5
70-74	Stroke 1116·6 (745·3 - 1553·2)	Ischemic heart disease 492·3 (289·1 - 782·0)	Chronic obstructive pulmonary disease 304·5 (187·2 - 453·0)	Tracheal, bronchus, and lung cancer 233·7 (147·8 - 344·6)	Diabetes mellitus 188·0 (108·4 - 316·5)	Stroke 498·6 (328·7 - 728·6)	Ischemic heart disease 193·3 (110·4 - 305·3)	Diabetes mellitus 186·2 (96·7 - 305·9)	Chronic obstructive pulmonary disease 136·2 (67·2 - 231·6)	Hypertensive heart disease 123·9 (60·4 - 232·8)
75-79	Stroke 2014·2 (1331·4 - 2720·9)	Ischemic heart disease 775·1 (454·4 - 1276·0)	Chronic obstructive pulmonary disease 596·9 (356·6 - 893·4)	Diabetes mellitus 285·4 (168·4 - 456·8)	Tracheal, bronchus, and lung cancer 244·7 (146·4 - 368·0)	Stroke 754·6 (525·9 - 1114·2)	Ischemic heart disease 281·1 (165·5 - 454·9)	Chronic obstructive pulmonary disease 235·7 (121·0 - 423·9)	Diabetes mellitus 229·1 (128·1 - 369·9)	Alzheimer's disease and other dementias 217·0 (49·0 - 544·1)
80-84	Stroke 2727·7 (1602·8 - 3841·2)	Ischemic heart disease 1210·4 (720·5 - 1985·0)	Chronic obstructive pulmonary disease 970·9 (581·7 - 1444·8)	Alzheimer's disease and other dementias 454·9 (88·6 - 1219·1)	Lower respiratory infections 381·8 (225·1 - 624·4)	Stroke 1135·0 (685·2 - 1652·8)	Alzheimer's disease and other dementias 639·9 (149·7 - 1496·8)	Ischemic heart disease 511·2 (289·5 - 814·2)	Chronic obstructive pulmonary disease 453·6 (211·6 - 769·6)	Diabetes mellitus 318·2 (169·0 - 510·5)
85-89	Stroke 3060·0 (1952·8 - 4352·0)	Ischemic heart disease 1711·1 (1014·9 - 2619·6)	Chronic obstructive pulmonary disease 1385·2 (794·1 - 2048·8)	Alzheimer's disease and other dementias 1047·7 (231·6 - 3001·1)	Lower respiratory infections 572·7 (332·9 - 924·9)	Stroke 1626·8 (905·8 - 2542·2)	Alzheimer's disease and other dementias 1427·6 (393·0 - 3536·4)	Ischemic heart disease 833·2 (443·5 - 1376·0)	Chronic obstructive pulmonary disease 799·8 (377·9 - 1333·5)	Hypertensive heart disease 483·0 (220·8 - 890·7)
90-94	Stroke 3374·6 (2065·3 - 5308·1)	Ischemic heart disease 2337·6 (1370·1 - 4019·7)	Alzheimer's disease and other dementias 2077·7 (474·0 - 4980·6)	Chronic obstructive pulmonary disease 1670·2 (956·4 - 2683·8)	Lower respiratory infections 867·0 (494·3 - 1424·0)	Alzheimer's disease and other dementias 2796·8 (739·9 - 7156·8)	Stroke 2028·6 (1151·5 - 3071·1)	Ischemic heart disease 1330·0 (692·3 - 2129·3)	Chronic obstructive pulmonary disease 1116·2 (546·4 - 1826·6)	Hypertensive heart disease 697·9 (283·5 - 1212·6)
95+	Stroke 3639·1 (1970·6 - 5687·4)	Alzheimer's disease and other dementias 3480·7 (780·6 - 7742·6)	Ischemic heart disease 2936·7 (1511·0 - 4961·4)	Chronic obstructive pulmonary disease 1924·0 (1084·7 - 3108·4)	Lower respiratory infections 1323·7 (713·7 - 2394·9)	Alzheimer's disease and other dementias 4649·4 (1175·1 - 10182·5)	Stroke 2391·6 (1161·9 - 3863·9)	Ischemic heart disease 1929·1 (930·7 - 3150·6)	Chronic obstructive pulmonary disease 1341·6 (600·4 - 2313·8)	Lower respiratory infections 1062·9 (486·2 - 1867·2)

Table S17 | Relative contribution (%) of top five Level 2 risk factors to the total mortality by sex and age groups in ASEAN, 2023

Age group (in years)	Males					Females				
	Top 1	Top 2	Top 3	Top 4	Top 5	Top 1	Top 2	Top 3	Top 4	Top 5
<1	Child and maternal malnutrition<br>48·4%<br>(42·1 - 55·4)	Air pollution<br>13·6%<br>(11·1 - 16·4)	Unsafe water, sanitation, and handwashing<br>4·4%<br>(1·8 - 7·9)	Tobacco<br>2·0%<br>(1·1 - 3·2)	Non-optimal temperature<br>0·2%<br>(0·1 - 0·5)	Child and maternal malnutrition<br>45·2%<br>(39·4 - 50·8)	Air pollution<br>13·2%<br>(10·8 - 15·6)	Unsafe water, sanitation, and handwashing<br>4·2%<br>(1·7 - 8·0)	Tobacco<br>2·2%<br>(1·2 - 3·2)	Non-optimal temperature<br>0·2%<br>(0·0 - 0·5)
2-4	Child and maternal malnutrition<br>17·2%<br>(12·2 - 23·2)	Unsafe water, sanitation, and handwashing<br>4·1%<br>(1·9 - 7·2)	Air pollution<br>2·4%<br>(1·0 - 4·1)	Tobacco<br>1·9%<br>(1·1 - 2·8)	Kidney dysfunction<br>0·8%<br>(0·4 - 1·5)	Child and maternal malnutrition<br>23·0%<br>(17·5 - 28·7)	Unsafe water, sanitation, and handwashing<br>5·0%<br>(2·4 - 8·5)	Air pollution<br>3·8%<br>(1·6 - 5·9)	Tobacco<br>2·8%<br>(1·8 - 3·8)	Kidney dysfunction<br>0·8%<br>(0·4 - 1·3)
5-9	Unsafe water, sanitation, and handwashing<br>2·8%<br>(1·1 - 5·9)	Air pollution<br>2·3%<br>(1·0 - 3·7)	Tobacco<br>1·6%<br>(1·0 - 2·4)	Kidney dysfunction<br>1·2%<br>(0·7 - 2·0)	Child and maternal malnutrition<br>0·8%<br>(0·5 - 1·3)	Unsafe water, sanitation, and handwashing<br>3·0%<br>(1·3 - 6·1)	Air pollution<br>2·4%<br>(1·0 - 4·2)	Tobacco<br>1·7%<br>(1·1 - 2·6)	Child and maternal malnutrition<br>1·5%<br>(1·1 - 1·9)	Kidney dysfunction<br>1·4%<br>(0·7 - 2·2)
10-14	Unsafe water, sanitation, and handwashing<br>2·0%<br>(0·8 - 4·4)	Kidney dysfunction<br>1·9%<br>(1·2 - 2·9)	Air pollution<br>1·6%<br>(0·6 - 2·5)	Tobacco<br>1·3%<br>(0·9 - 1·8)	Non-optimal temperature<br>0·8%<br>(0·6 - 1·1)	Kidney dysfunction<br>2·5%<br>(1·4 - 3·7)	Unsafe water, sanitation, and handwashing<br>2·3%<br>(1·0 - 4·5)	Air pollution<br>1·7%<br>(0·7 - 2·8)	Tobacco<br>1·4%<br>(0·9 - 1·8)	Child and maternal malnutrition<br>1·2%<br>(0·9 - 1·5)
15-19	Occupational risks<br>4·8%<br>(3·3 - 6·5)	High alcohol use<br>2·0%<br>(1·4 - 2·6)	Kidney dysfunction<br>1·9%<br>(1·2 - 2·8)	Non-optimal temperature<br>1·3%<br>(1·0 - 1·6)	Unsafe sex<br>1·0%<br>(0·6 - 1·6)	Kidney dysfunction<br>4·3%<br>(2·6 - 6·1)	Occupational risks<br>1·9%<br>(1·2 - 2·8)	Unsafe sex<br>1·5%<br>(1·0 - 2·3)	Child and maternal malnutrition<br>1·5%<br>(0·9 - 2·0)	High fasting plasma glucose<br>1·3%<br>(0·9 - 1·9)
20-24	Occupational risks<br>9·2%<br>(6·8 - 12·0)	High alcohol use<br>4·0%<br>(2·9 - 5·3)	Kidney dysfunction<br>2·5%<br>(1·6 - 3·7)	Unsafe sex<br>2·4%<br>(1·2 - 3·6)	Non-optimal temperature<br>1·4%<br>(1·2 - 1·7)	Kidney dysfunction<br>5·0%<br>(3·1 - 7·0)	Occupational risks<br>3·7%<br>(2·4 - 5·4)	Unsafe sex<br>3·4%<br>(2·2 - 4·8)	Child and maternal malnutrition<br>2·1%<br>(1·2 - 2·8)	Intimate partner violence<br>1·5%<br>(0·3 - 3·5)
25-29	Occupational risks<br>9·9%<br>(7·6 - 12·8)	Dietary risks<br>5·4%<br>(0·3 - 7·8)	High alcohol use<br>5·2%<br>(4·0 - 6·6)	Unsafe sex<br>4·2%<br>(2·2 - 6·2)	High systolic blood pressure<br>4·0%<br>(2·4 - 5·6)	Unsafe sex<br>6·2%<br>(4·0 - 8·7)	Dietary risks<br>6·1%<br>(0·1 - 9·2)	Kidney dysfunction<br>5·9%<br>(4·3 - 7·7)	Air pollution<br>4·3%<br>(3·1 - 5·7)	Occupational risks<br>3·6%<br>(2·5 - 5·1)
30-34	Tobacco<br>12·6%<br>(10·1 - 15·4)	Occupational risks<br>8·5%<br>(6·7 - 11·3)	Dietary risks<br>8·4%<br>(0·9 - 12·1)	High systolic blood pressure	High alcohol use<br>6·1%<br>(4·6 - 8·1)	Dietary risks<br>9·2%<br>(0·2 - 13·9)	Unsafe sex<br>7·4%<br>(5·3 - 9·9)	Kidney dysfunction<br>7·0%<br>(5·0 - 9·1)	High systolic blood pressure	Air pollution<br>5·6%<br>(4·1 - 7·3)

Age group (in years)	Males					Females				
	Top 1	Top 2	Top 3	Top 4	Top 5	Top 1	Top 2	Top 3	Top 4	Top 5
				6·6% (4·2 - 9·0)					5·6% (3·5 - 8·4)	
35-39	Tobacco 15·8% (12·7 - 18·7)	Dietary risks 11·3% (1·8 - 15·6)	High systolic blood pressure 10·0% (6·9 - 13·2)	High alcohol use 7·2% (5·4 - 9·7)	Air pollution 6·9% (5·1 - 9·0)	Dietary risks 11·5% (0·5 - 16·8)	High systolic blood pressure 8·8% (5·9 - 12·3)	Unsafe sex 7·5% (5·3 - 9·7)	Kidney dysfunction 7·0% (5·1 - 8·9)	Air pollution 6·4% (4·7 - 8·1)
40-44	Tobacco 19·5% (16·0 - 22·8)	Dietary risks 14·2% (2·9 - 19·9)	High systolic blood pressure 14·1% (10·4 - 18·0)	Air pollution 8·5% (6·5 - 10·5)	High LDL cholesterol 8·4% (6·0 - 10·8)	Dietary risks 14·6% (1·1 - 21·7)	High systolic blood pressure 14·1% (10·2 - 18·8)	High body-mass index 8·0% (4·0 - 12·0)	Air pollution 8·0% (5·9 - 10·0)	Kidney dysfunction 7·8% (6·2 - 9·3)
45-49	Tobacco 23·1% (19·4 - 26·7)	High systolic blood pressure 19·2% (15·0 - 23·4)	Dietary risks 17·5% (3·6 - 24·8)	Air pollution 9·9% (7·5 - 12·2)	High LDL cholesterol 9·8% (6·8 - 12·7)	High systolic blood pressure 18·8% (14·3 - 24·4)	Dietary risks 16·7% (2·9 - 24·6)	Air pollution 9·4% (7·2 - 11·7)	High body-mass index 9·2% (5·1 - 13·4)	Kidney dysfunction 8·0% (6·4 - 9·4)
50-54	Tobacco 26·2% (22·4 - 30·0)	High systolic blood pressure 22·5% (18·0 - 26·8)	Dietary risks 18·2% (4·4 - 26·1)	Air pollution 11·5% (8·9 - 14·1)	High LDL cholesterol 10·1% (7·1 - 13·1)	High systolic blood pressure 21·7% (16·7 - 27·3)	Dietary risks 16·8% (3·1 - 25·2)	High body-mass index 11·0% (5·6 - 16·3)	Air pollution 10·7% (8·2 - 13·3)	High fasting plasma glucose 9·2% (7·3 - 11·3)
55-59	Tobacco 26·3% (22·9 - 29·9)	High systolic blood pressure 25·0% (19·8 - 29·9)	Dietary risks 19·4% (7·5 - 27·9)	Air pollution 12·5% (9·9 - 15·3)	High LDL cholesterol 9·4% (6·4 - 12·4)	High systolic blood pressure 23·6% (18·3 - 28·6)	Dietary risks 17·3% (4·9 - 25·8)	Air pollution 11·9% (9·0 - 14·7)	High fasting plasma glucose 11·1% (8·7 - 13·4)	High body-mass index 10·5% (5·5 - 15·6)
60-64	Tobacco 27·0% (22·9 - 30·9)	High systolic blood pressure 26·8% (22·0 - 31·5)	Dietary risks 18·9% (7·9 - 27·5)	Air pollution 13·6% (10·7 - 16·5)	High fasting plasma glucose 10·0% (8·3 - 12·1)	High systolic blood pressure 25·5% (20·5 - 30·4)	Dietary risks 16·8% (5·3 - 25·6)	Air pollution 13·3% (10·2 - 16·3)	High fasting plasma glucose 12·3% (10·1 - 14·8)	High body-mass index 9·6% (5·0 - 14·8)
65-69	High systolic blood pressure 26·3% (21·5 - 31·2)	Tobacco 25·3% (21·2 - 29·1)	Dietary risks 18·6% (8·6 - 26·9)	Air pollution 14·5% (11·5 - 17·6)	High fasting plasma glucose 10·8% (9·1 - 13·1)	High systolic blood pressure 27·0% (21·3 - 32·0)	Dietary risks 16·9% (6·3 - 26·0)	Air pollution 14·7% (11·5 - 18·2)	High fasting plasma glucose 13·4% (11·1 - 16·1)	Kidney dysfunction 9·7% (7·9 - 11·7)
70-74	High systolic blood pressure 27·1% (22·3 - 31·8)	Tobacco 23·3% (19·5 - 27·0)	Dietary risks 16·5% (6·9 - 24·8)	Air pollution 15·3% (11·9 - 18·4)	High fasting plasma glucose 10·7% (8·8 - 13·0)	High systolic blood pressure 28·2% (22·5 - 33·5)	Air pollution 15·9% (12·5 - 19·8)	Dietary risks 15·5% (5·3 - 24·2)	High fasting plasma glucose 13·5% (11·4 - 16·2)	Kidney dysfunction 9·6% (7·5 - 11·7)
75-79	High systolic blood pressure 26·4% (21·6 - 31·2)	Tobacco 21·8% (18·0 - 25·5)	Air pollution 16·0% (12·6 - 19·4)	Dietary risks 14·9% (6·1 - 22·6)	High fasting plasma glucose 10·5% (8·7 - 12·6)	High systolic blood pressure 28·2% (22·1 - 33·8)	Air pollution 16·9% (12·8 - 21·1)	Dietary risks 14·4% (5·1 - 22·7)	High fasting plasma glucose 13·2% (10·8 - 15·6)	Kidney dysfunction 8·9% (6·9 - 11·0)

Age group (in years)	Males					Females				
	Top 1	Top 2	Top 3	Top 4	Top 5	Top 1	Top 2	Top 3	Top 4	Top 5
80-84	High systolic blood pressure 25·0% (19·9 - 29·8)	Tobacco 19·6% (15·9 - 23·6)	Air pollution 16·5% (13·2 - 20·5)	Dietary risks 12·5% (4·8 - 19·4)	High fasting plasma glucose 10·1% (8·4 - 11·9)	High systolic blood pressure 27·4% (21·5 - 33·2)	Air pollution 17·9% (13·5 - 22·7)	Dietary risks 12·8% (4·5 - 20·2)	High fasting plasma glucose 12·3% (10·5 - 14·7)	Kidney dysfunction 7·9% (5·5 - 10·1)
85-89	High systolic blood pressure 22·4% (17·3 - 27·5)	Tobacco 19·0% (15·5 - 22·8)	Air pollution 16·7% (12·9 - 20·6)	Dietary risks 11·4% (4·8 - 17·9)	High fasting plasma glucose 9·5% (7·9 - 11·2)	High systolic blood pressure 24·9% (18·5 - 30·8)	Air pollution 18·1% (13·7 - 23·6)	Dietary risks 11·5% (3·9 - 18·3)	High fasting plasma glucose 10·9% (9·0 - 12·9)	Kidney dysfunction 8·3% (6·0 - 10·8)
90-94	High systolic blood pressure 19·7% (14·3 - 24·4)	Air pollution 16·3% (12·4 - 21·7)	Tobacco 14·1% (10·8 - 17·6)	Dietary risks 10·1% (3·9 - 16·0)	High fasting plasma glucose 9·3% (7·7 - 11·4)	High systolic blood pressure 22·2% (15·6 - 27·6)	Air pollution 18·2% (12·9 - 25·6)	High fasting plasma glucose 10·3% (8·2 - 12·3)	Dietary risks 10·2% (3·2 - 16·7)	Kidney dysfunction 8·3% (5·7 - 10·7)
95+	High systolic blood pressure 17·3% (11·6 - 22·6)	Tobacco 16·0% (12·3 - 20·2)	Air pollution 15·4% (11·0 - 22·0)	High fasting plasma glucose 9·3% (7·5 - 11·4)	Kidney dysfunction 9·3% (6·5 - 12·2)	High systolic blood pressure 20·7% (13·8 - 27·1)	Air pollution 17·4% (11·5 - 25·9)	High fasting plasma glucose 9·6% (7·5 - 12·0)	Dietary risks 9·1% (3·1 - 15·2)	Kidney dysfunction 8·5% (5·4 - 11·3)

Table S18 | Absolute number of deaths attributable to the top five Level 2 risk factors by sex and age groups in ASEAN, 2023

	Males					Females				
**Age group**	**Top 1**	**Top 2**	**Top 3**	**Top 4**	**Top 5**	**Top 1**	**Top 2**	**Top 3**	**Top 4**	**Top 5**
<1	Child and maternal malnutrition 57 800 (48 900–66 600)	Air pollution 16 200 (13 300–19 600)	Unsafe water, sanitation, and handwashing 5 310 (2 080–9 150)	Tobacco 2 410 (1 360–3 830)	Non-optimal temperature 266 (73–589)	Child and maternal malnutrition 38 800 (33 400–45 000)	Air pollution 11 300 (9 310–13 800)	Unsafe water, sanitation, and handwashing 3 650 (1 420–7 100)	Tobacco 1 860 (1 050–2 800)	Non-optimal temperature 183 (27–413)
2-4	Child and maternal malnutrition 3 290 (2 280–4 770)	Unsafe water, sanitation, and handwashing 792 (369–1 400)	Air pollution 460 (179–858)	Tobacco 359 (210–570)	Kidney dysfunction 159 (84–299)	Child and maternal malnutrition 3 090 (2 230–4 020)	Unsafe water, sanitation, and handwashing 674 (314–1 150)	Air pollution 510 (228–803)	Tobacco 372 (227–526)	Kidney dysfunction 101 (47–178)
5-9	Unsafe water, sanitation, and handwashing 452 (181–981)	Air pollution 363 (147–593)	Tobacco 252 (155–377)	Kidney dysfunction 193 (106–326)	Child and maternal malnutrition 133 (74–200)	Unsafe water, sanitation, and handwashing 379 (176–722)	Air pollution 295 (116–521)	Tobacco 218 (132–343)	Child and maternal malnutrition 185 (136–245)	Kidney dysfunction 170 (90–264)
10-14	Unsafe water, sanitation, and handwashing 297 (124–697)	Kidney dysfunction 283 (170–462)	Air pollution 237 (96–380)	Tobacco 190 (127–279)	Non-optimal temperature 127 (87–167)	Kidney dysfunction 282 (157–405)	Unsafe water, sanitation, and handwashing 251 (115–494)	Air pollution 183 (72–320)	Tobacco 151 (100–213)	Child and maternal malnutrition 130 (101–174)
15-19	Occupational risks 1 570 (1 080–2 230)	High alcohol use 651 (474–842)	Kidney dysfunction 634 (403–956)	Non-optimal temperature 439 (335–529)	Unsafe sex 335 (189–516)	Kidney dysfunction 905 (539–1 310)	Occupational risks 393 (248–614)	Unsafe sex 321 (202–461)	Child and maternal malnutrition 313 (178–448)	High fasting plasma glucose 279 (168–402)
20-24	Occupational risks 3 950 (2 740–5 650)	High alcohol use 1 710 (1 290–2 150)	Kidney dysfunction 1 080 (667–1 630)	Unsafe sex 1 000 (499–1 520)	Non-optimal temperature 600 (490–731)	Kidney dysfunction 1 400 (863–2 020)	Occupational risks 1 050 (664–1 580)	Unsafe sex 946 (628–1 330)	Child and maternal malnutrition 599 (339–824)	Intimate partner violence 426 (78–980)
25-29	Occupational risks 4 880 (3 700–6 750)	Dietary risks 2 670 (127–4 030)	High alcohol use 2 570 (1 970–3 270)	Unsafe sex 2 050 (1 100–3 050)	High systolic blood pressure 1 960 (1 140–2 750)	Unsafe sex 1 960 (1 250–2 820)	Dietary risks 1 920 (20–2 980)	Kidney dysfunction 1 870 (1 360–2 550)	Air pollution 1 370 (941–1 830)	Occupational risks 1 150 (794–1 630)
30-34	Tobacco 8 620 (6 670–10 800)	Occupational risks 5 830 (4 450–8 000)	Dietary risks 5 770 (607–8 380)	High systolic blood pressure 4 530 (2 760–6 230)	High alcohol use 4 190 (3 190–5 460)	Dietary risks 3 940 (80–6 050)	Unsafe sex 3 160 (2 240–4 250)	Kidney dysfunction 2 970 (2 070–4 050)	High systolic blood pressure 2 400 (1 490–3 640)	Air pollution 2 370 (1 730–3 090)

Age group	Males					Females				
	Top 1	Top 2	Top 3	Top 4	Top 5	Top 1	Top 2	Top 3	Top 4	Top 5
35-39	Tobacco 13 700 (10 800–17 100)	Dietary risks 9 880 (1 540–14 200)	High systolic blood pressure 8 750 (5 870–11 400)	High alcohol use 6 300 (4 700–8 370)	Air pollution 6 040 (4 390–8 120)	Dietary risks 5 980 (267–9 330)	High systolic blood pressure 4 570 (3 020–6 630)	Unsafe sex 3 870 (2 740–5 400)	Kidney dysfunction 3 640 (2 580–4 900)	Air pollution 3 330 (2 430–4 410)
40-44	Tobacco 22 000 (17 400–26 800)	Dietary risks 16 000 (2 970–23 400)	High systolic blood pressure 15 900 (11 600–21 100)	Air pollution 9 550 (7 180–12 300)	High LDL cholesterol 9 520 (6 580–13 000)	Dietary risks 11 300 (840–18 000)	High systolic blood pressure 11 000 (7 560–15 400)	High body-mass index 6 250 (3 150–9 850)	Air pollution 6 190 (4 550–8 380)	Kidney dysfunction 6 050 (4 560–7 890)
45-49	Tobacco 35 600 (28 600–43 500)	High systolic blood pressure 29 500 (22 500–38 800)	Dietary risks 26 900 (5 760–39 600)	Air pollution 15 200 (11 400–19 700)	High LDL cholesterol 15 100 (10 400–21 700)	High systolic blood pressure 18 000 (13 200–24 700)	Dietary risks 15 900 (2 690–24 300)	Air pollution 8 950 (6 790–11 800)	High body-mass index 8 850 (4 730–13 600)	High LDL cholesterol 7 950 (5 070–11 600)
50-54	Tobacco 54 900 (45 200–67 000)	High systolic blood pressure 47 100 (34 900–60 000)	Dietary risks 38 100 (9 480–57 100)	Air pollution 24 000 (18 500–30 300)	High LDL cholesterol 21 200 (13 900–28 400)	High systolic blood pressure 26 300 (19 500–35 700)	Dietary risks 20 300 (3 490–31 300)	High body-mass index 13 300 (6 560–20 000)	Air pollution 13 000 (9 680–17 200)	High fasting plasma glucose 11 100 (8 430–14 300)
55-59	Tobacco 64 600 (52 800–77 600)	High systolic blood pressure 61 400 (47 000–77 600)	Dietary risks 47 500 (17 600–70 600)	Air pollution 30 700 (23 300–39 300)	High LDL cholesterol 23 100 (15 000–32 100)	High systolic blood pressure 34 600 (25 800–44 100)	Dietary risks 25 500 (6 890–40 400)	Air pollution 17 400 (13 200–21 900)	High fasting plasma glucose 16 200 (12 800–20 600)	High body-mass index 15 500 (7 780–23 600)
60-64	Tobacco 81 600 (66 100–97 000)	High systolic blood pressure 81 100 (63 300–100 000)	Dietary risks 57 300 (23 500–87 500)	Air pollution 41 100 (30 600–51 700)	High fasting plasma glucose 30 200 (24 000–38 600)	High systolic blood pressure 48 000 (37 100–60 500)	Dietary risks 31 600 (9 260–49 200)	Air pollution 25 000 (18 900–32 200)	High fasting plasma glucose 23 100 (18 700–28 400)	High body-mass index 18 100 (9 060–27 900)
65-69	High systolic blood pressure 78 900 (59 100–97 700)	Tobacco 75 700 (61 700–91 400)	Dietary risks 55 700 (25 700–83 500)	Air pollution 43 400 (34 500–55 700)	High fasting plasma glucose 32 400 (25 500–40 700)	High systolic blood pressure 54 700 (41 800–68 200)	Dietary risks 34 200 (12 200–52 400)	Air pollution 29 800 (23 100–38 100)	High fasting plasma glucose 27 200 (22 400–33 700)	Kidney dysfunction 19 600 (15 600–24 500)
70-74	High systolic blood pressure 77 600 (59 800–94 800)	Tobacco 66 800 (54 700–80 800)	Dietary risks 47 300 (20 700–72 300)	Air pollution 43 800 (34 200–54 200)	High fasting plasma glucose 30 700 (23 600–39 100)	High systolic blood pressure 60 000 (46 200–75 200)	Air pollution 33 700 (25 800–42 400)	Dietary risks 33 000 (11 300–52 300)	High fasting plasma glucose 28 800 (23 300–35 800)	Kidney dysfunction 20 300 (15 800–25 600)
75-79	High systolic blood pressure 58 000 (45 100–70 300)	Tobacco 47 700 (39 300–58 200)	Air pollution 35 000 (28 500–43 600)	Dietary risks 32 700 (14 100–49 000)	High fasting plasma glucose 23 100 (18 100–28 500)	High systolic blood pressure 53 500 (42 400–65 600)	Air pollution 32 000 (24 600–41 500)	Dietary risks 27 400 (9 770–43 500)	High fasting plasma glucose 25 000 (20 300–30 100)	Kidney dysfunction 16 900 (13 300–21 000)

Age group	Males					Females				
	Top 1	Top 2	Top 3	Top 4	Top 5	Top 1	Top 2	Top 3	Top 4	Top 5
80-84	High systolic blood pressure 43 900 (33 800–54 100)	Tobacco 34 500 (27 800–41 400)	Air pollution 28 900 (22 500–35 700)	Dietary risks 21 900 (8 740–33 700)	High fasting plasma glucose 17 700 (14 300–22 500)	High systolic blood pressure 53 400 (40 400–67 800)	Air pollution 34 800 (25 600–44 500)	Dietary risks 24 900 (8 830–39 900)	High fasting plasma glucose 23 900 (19 600–28 700)	Kidney dysfunction 15 300 (10 900–20 100)
85-89	High systolic blood pressure 26 000 (19 600–32 100)	Tobacco 22 100 (17 800–27 000)	Air pollution 19 300 (15 000–24 700)	Dietary risks 13 200 (5 640–21 000)	High fasting plasma glucose 11 000 (8 940–13 600)	High systolic blood pressure 42 300 (31 200–53 500)	Air pollution 30 800 (22 100–40 500)	Dietary risks 19 700 (6 690–31 400)	High fasting plasma glucose 18 600 (14 900–22 300)	Kidney dysfunction 14 100 (10 100–18 100)
90-94	High systolic blood pressure 12 200 (8 600–15 300)	Air pollution 10 100 (7 450–13 800)	Tobacco 8 750 (6 510–11 300)	Dietary risks 6 230 (2 510–10 100)	High fasting plasma glucose 5 790 (4 680–7 040)	High systolic blood pressure 24 800 (17 100–32 300)	Air pollution 20 300 (13 700–28 700)	High fasting plasma glucose 11 500 (8 940–14 100)	Dietary risks 11 400 (3 620–18 500)	Kidney dysfunction 9 240 (6 530–11 900)
95+	High systolic blood pressure 5 560 (3 890–7 410)	Tobacco 5 120 (3 890–6 670)	Air pollution 4 950 (3 380–7 170)	High fasting plasma glucose 2 980 (2 280–3 760)	Kidney dysfunction 2 980 (2 050–4 060)	High systolic blood pressure 14 600 (9 270–19 500)	Air pollution 12 300 (7 660–18 100)	High fasting plasma glucose 6 790 (5 180–8 580)	Dietary risks 6 400 (2 230–10 500)	Kidney dysfunction 5 990 (3 890–8 270)

Author list

Taishun Li*, Periklis Charalampous*, Stephanie C C van der Lubbe*, Yawen Zhou, Simon I Hay, Jeofrey Bautista Abalos, Edimansyah Abdin, Hanif Abdul Rahman, Asnawi Abdullah, Abdulrakib Abdulrahim, Michael R M Abrigo, Wirawan Adikusuma, Tawseef Ahmad, Sima Ahmadpour, Mohamed Mustaf Ahmed, Budi Aji, Mohammed Alfaqeeh, Khaidar Ali, Syed Mohamed Aljunid, Joseph Uy Almazan, Ali Salman Al-Shami, Junaid Amin, Roshan A Ananda, Chin-Siang Ang, Nguyen Hoang Anh, Sumadi Lukman Anwar, Irfan Ardani, Rizal Arifin, Keivan Armani, Mohammad Ashfaq, Muhammad Shahzad Aslam, Yuni Asri, Zaw Zaw Aung, Zulfa Ayuningsih, Fitriyono Ayustaningwarno, Abul Kalam Azad, Mohd Yusmaidie Aziz, Ahmed Bali, Muhammad Irfan Bashir, Bagas Suryo Bintoro, Ahmad Naoras Bitar, Ai Cao My Bui, Asmat Burhan, Muhammad Daoud Butt, Dimples Acquiatan Camporedondo, Yu Cao, Hana Chen, Nicholas WS Chew, Ruixin Chi, Bryan Chong, Dennis Chong, Karleen Chong, Christina Malini Christopher, Dinh-Toi Chu, Mayank Dalakoti, Pojsakorn Danpanichkul, Bryan Gervais de Liyis, Edward Christopher Dee, Sadia Din, Mai Ngoc Do, Thao Huynh Phuong Do, Minh Cuong Duong, Angel Belle Cheng Dy, Ferry Efendi, Ekawati Ekawati, Muhammad Esa, Maryam Farooqui, Nelsensius Klau Fauk, Neeraj Kumar Fuloria, Shivkanya Fuloria, Francis Sarial Ganancial, Fernando Jr Barroga Garcia, Adrian Eleazar Go, Bey Hing Goh, Ernesto Ramos Gregorio, Nguyen Hai Nam, Harapan Harapan, Arief Hargono, Andy Martahan Andreas Hariandja, Faizul Hasan, Zaw Myo Hein, Rohman Hikmat, Aaron Kwun Hang Ho, Tinh Ho Huu, Mehdi Hosseinzadeh, Mega Hasanul Huda, Fadly Husain, Mohd Salami Ibrahim, Umar Idris Ibrahim, Korakrit Imwattana, Lalu Muhammad Irham, Fahrul Islam, Jazlan Jamaluddin, Roland Dominic G Jamora, Hanish Singh Jayasingh Chellammal, Yovanthi Anurangi Jayasinghe, Jost B Jonas, Muhammad Khan, Parisa Khoshvaght, Siti Khuzaiyah, Ko Ko, Soewarta Kosen, Chong-Han Kua, Garry Kuan, Yee Cheng Kueh, Maria Dyah Kurniasari, Dea Anita Ariani Kurniasih, Asep Kusnali, Aria Kusuma, Dian Kusuma, Daphne Teck Ching Lai, Agung Dwi Laksono, Tri Laksono, Jerrald Lau, Bao Huy Le, Thao Thi Thu Le, Hoi Leong Lee, Shaun Wen Huey Lee, Elvynna Leong, Mohammad Farris Iman Leong Bin Abdullah, Retno Lestari, Mei Fong Liew, Lee-Ling Lim, Donglin Liu, Xiao Liu, Arianna Maever Loreche, Hung N Luu, Kaung Suu Lwin, Zheng Feei Ma, Melani Ratih Mahanani, Roy Rillera Marzo, Richard James Maude, Muhammad Miftahussurur, Muhammad Agus Mikrajab, Long Chiau Ming, Maziar Moradi-Lakeh, Amartya Mukhopadhyay, Lutfi Muzaqi, Kiran P Nagdeo, Firzan Nainu, Tam Thi Ngo, Cao Duy Nguyen, Ha Thu Nguyen, Hien Thu Nguyen, Hien Thi Nguyen, Long Hoang Nguyen, Minh Thien Nguyen, Minh-Ha Nguyen, Nghia Phu Nguyen, Thanh Van Nguyen, Thinh Gia Nguyen, Luong Nguyen Thanh, Cham Thi Nguyen Thi, Ricvan Dana Nindrea, Mohammed Noushad, Nurfatimah Nurfatimah, Ahmad Dzulfikri Nurhan, Sok King Ong, Sandersan Onie, Zhinya Kawa Othman, Helena Ullyartha Pangaribuan, Santa Maria Pangaribuan, Bunga A Paramashanti, Veincent Christian Filipino Pepito, Tung Thanh Pham, Dimitri Poddighe, Kian Keong Poh, Thantrira Porntaveetus, Ragil Angga Prastiya, Yusuf Alif Pratama, Hery Purnobasuki, Nuzul Qur'aniati, Ali Qureshi, Basuki Rachmat, Asad Ur Rahman, Vignesh Ramachandran, Kadar Ramadhan, Sheena Ramazanu, Ateeq Rehman, Yohanes Andy Rias, Erna Rochmawati, Alina Rodriguez, Iftitakhur Rohmah, Thanakorn Rojanathagoon, Chanapong Rojanaworarit, Debby Syahru Romadlon, Betty Roosihermiatie, Bedanta Roy, Suebsarn Ruksakulpiwat, Ahmad Sahban Rafsanjani, Shuaibu Musa Saidu, Yoseph Leonardo Samodra, Lintang Dian Saraswati, Shilpa Sarawagi, Ni Putu Wulan

Purnama Sari, Mohammad-Ali Sarvghadi, Tual Sawn Khai, Abrar Mohammad Sayaf, KT Matthew Seah, Zhi Huang See, Siddharthan Selvaraj, Shazlin Shaharudin, JiaHao Shi, Ching-Hui Sia, Robert Sinto, Chandrashekhar T Sreeramareddy, Vetriselvan Subramaniyan, Chee Fai Sui, Desy Sulistiyorini, Henry Surendra, Suryanti Suryanti, Herry Susanto, Hani Susianti, Tri Nugraha Susilawati, Dayinta Annisa Syaiful, Balamurugan Tagiisuran, Ching Siang Tan, Kit-Aun Tan, Ekamol Tantisattamo, Ingan Ukur Tarigan, Andrew W Taylor-Robinson, Van Chi Thai, Lakshmi Thangavelu, Hein Thu, Jansje Henny Vera Ticoalu, Santo Imanuel Tonapa, Nam Minh-phuong Tran, Nam Hoang Tran, Quynh Thuy Huong Tran, Thang Huu Tran, Thi Thuy Vy Tran, Nguyen Tran Minh Duc, Indang Trihandini, Quynh Xuan Nguyen Truong, Abdul Rohim Tualeka, Sok Cin Tye, Josefien van Olmen, Narayanaswamy Venketasubramanian, Nguyen-Kieu Viet-Nhi, Tuan Vinh, Lan Thi Hoang Vu, Linh Vu, Thinh Toan Vu, Ali Wagdy, Mugi Wahidin, Tati Suryati Warouw, Apichai Wattanapisit, Taweewat Wiangkham, Yohanes Cakrapradipta Wibowo, Akhmad Fajri Widodo, Angga Wilandika, Sri Winarni, Utoomporn Wongsin, Mun Fei Yam, Kwang Mo Yang, Siyan Yi, Mustafa Younis, Riky Tri Yunardi, Emilia Zainal Abidin, Lilik Zuhriyah, Mohsen Naghavi†, Marie Ng†, and Cuilin Zhang†.

*Co-first authors

†Co-senior authors

Authors' affiliations

Nanjing Drum Tower Hospital, Affiliated Hospital of Medical School (T Li PhD), Nanjing University, Nanjing, China; Global Center for Asian Women's Health (GloW), Yong Loo Lin School of Medicine (T Li PhD), National University of Singapore, Singapore, ; Yong Loo Lin School of Medicine (P Charalampous PhD, N W Chew MD, C Sia MSCI, Prof N Venketasubramanian MSc), NUS-IHME Global Burden of Disease Research Centre, Yong Loo Lin School of Medicine (S C C van der Lubbe PhD, Y Zhou MD, M Ng PhD), Saw Swee Hock School of Public Health (E Abdin PhD, S Ramazanu PhD, H Thu MPH, Prof S Yi PhD), Faculty of Dentistry (Y Cao BSc), Department of Medicine (B Chong MBBS, M Liew MRCP, Prof K Poh MD), Cardiovascular Metabolic Translational Research Program (M Dalakoti MPH), Department of Surgery (J Lau PhD), Department of Psychological Medicine (Prof A Rodriguez PhD), Department of Family Medicine (Prof J van Olmen PhD), Global Center for Asian Women's Health (GloW), Yong Loo Lin School of Medicine (Prof C Zhang MD), Department of Obstetrics & Gynaecology, Yong Loo Lin School of Medicine (Prof C Zhang MD), National University of Singapore, Singapore, Singapore; Department of Public Health (P Charalampous PhD), Erasmus University Medical Center, Rotterdam, Netherlands; Institute for Health Metrics and Evaluation (Prof S I Hay DSc, Prof M Naghavi PhD, M Ng PhD), Department of Health Metrics Sciences, School of Medicine (Prof S I Hay DSc, Prof M Naghavi PhD), University of Washington, Seattle, WA, USA; University of Eastern Pangasinan, Binalonan, Philippines (J B Abalos PhD); Research Division (E Abdin PhD), Institute of Mental Health, Singapore, Singapore; Institute of Health Sciences (H Abdul Rahman PhD), University of Brunei Darussalam, Gadong, Brunei; National Health Policy Agency (Prof A Abdullah PhD), Ministry of Health, Jakarta, Indonesia; Universitas Muhammadiyah Aceh, Banda Aceh, Indonesia (Prof A Abdullah PhD); Department of Medical Microbiology (A Abdulrahim MSc), Department of Psychiatry (K Tan PhD), Department of Environmental and Occupational Health (E Zainal Abidin PhD), Universiti Putra Malaysia, Serdang, Malaysia; Philippine Institute for Development Studies, Quezon City, Philippines (M R M Abrigo PhD); Research Center for Computing (W Adikusuma PhD), National Research and Innovation Agency (BRIN), Cibinong, Indonesia; Department of Clinical Pharmacy (T Ahmad PhD), Prince of Songkla University, Hat-Yai, Thailand; Department of Pharmacy (T Ahmad PhD), COMSATS Institute of Information Technology, Abbottabad, Pakistan; Department of Information System (S Ahmadpour PhD), Universiti Tunku Abdul Rahman, Kampar, Malaysia; Institute for Global Health (M M Ahmed MD), SIMAD University, Mogadishu, Somalia; Faculty of Medicine and Public Health (B Aji DrPH), Department of Public Health (K Ali MPH), Center of Sustainable Development Goals (K Ali MPH), Jenderal Soedirman University, Purwokerto, Indonesia; Department of Pharmacology and Clinical Pharmacy (M Alfaqeeh PhD), Faculty of Law (A Kusnali MA), Padjadjaran University, Bandung, Indonesia; Department of Public Health and Community Medicine (Prof S M Aljunid PhD, Prof C T Sreeramareddy MD), International Medical University, Kuala Lumpur, Malaysia; International Centre for Casemix and Clinical Coding (Prof S M Aljunid PhD), National University of Malaysia, Bandar Tun Razak, Malaysia; Department of Medicine (J U Almazan PhD), Nazarbayev University, Astana, Kazakhstan; Department of Pharmacology (A S Al-Shami MS), Sana'a University, Sanaa, Yemen; Department of Basic Medical Sciences (A S Al-Shami MS), Universiti Malaysia Sarawak, Kota Samarahan, Malaysia; Department of Physiotherapy (J Amin MSc), University of Hail, Hail, Saudi

Arabia; Department of Orthopaedic Rehabilitation (J Amin MSc), University of Science Malaysia, Kubang Kerian, Malaysia; Department of General Medicine (R A Ananda MD), Eastern Health, Box Hill, VIC, Australia; Department of Primary Care and Public Health (R A Ananda MD), School of Public Health (K Armani PhD), Imperial College London, London, England; World Health Organization for Digital Health and Health Education (C Ang PhD), Lee Kong Chian School of Medicine (R Chi PhD), National Institute of Education (S Shaharudin PhD), Nanyang Technological University, Singapore, Singapore; Department of General Medicine (N Anh MD), Thai Binh University of Medicine and Pharmacy in Vietnam, Thai Binh City, Viet Nam; Ophthalmology Department (N Anh MD), Hoa Binh General Hospital, Hoa Binh City, Viet Nam; Department of Surgery (S Anwar PhD), Gadjah Mada University, Yogyakarta, Indonesia; Research Center for Public Health and Nutrition (I Ardani MPH, D A Kurniasih PhD), Research Organization for Health (M Wahidin PhD), Health Unit Organization (T S Warouw DrPH), National Research and Innovation Agency (BRIN), Bogor, Indonesia (A Kusnali MA); Faculty of Engineering (R Arifin PhD), Universitas Muhammadiyah Ponorogo, Ponorogo, Indonesia; Faculty of Pharmaceutical Sciences (K Armani PhD), UCSI University, Kuala Lumpur, Malaysia; Research & Development Cell (M Ashfaq PhD), Woxsen University, Hyderabad, India; Faculty of Engineering (M Ashfaq PhD), Center of Excellence in Precision Medicine and Digital Health (S Din PhD, Prof M Khan PhD, T Porntaveetus PhD, Prof A Wagdy PhD), Faculty of Nursing (F Hasan PhD, I Rohmah PhD, D S Romadlon PhD), Department of Pharmaceutical Sciences and Technology (Z K Othman MSc), School of Global Health (S M Saidu MSc), College of Public Health Sciences (J Shi MPH), Chulalongkorn University, Bangkok, Thailand; School of Traditional Chinese Medicine (M Aslam PhD), Xiamen University Malaysia, Sepang, Malaysia; Nursing Department (Y Asri PhD), Faculty of Health Science (Y Asri PhD), Institute of Technology and Health Science RS Dr Soepraoen, Malang, Indonesia; Department of Health Outcome and Policy Evaluation (Z Aung MPH), Thailand National Health Foundation, Bangkok, Thailand; Department of Nutrition (Z Ayuningsih MEpid), Universitas Islam Negeri Walisongo, Semarang, Indonesia; Nutrition Science Department (F Ayustaningwarno PhD), Center of Nutrition Research (CENURE) (F Ayustaningwarno PhD), Occupational Safety and Health Study Program (E Ekawati MSc), Department of Epidemiology and Tropical Disease (L D Saraswati PhD), Diponegoro University, Semarang, Indonesia; Department of Pharmacy (A Azad PhD), Universiti Antarabangsa MAIWP, Kuala Lumpur, Malaysia; Advanced Medical & Dental Institute (M Aziz PhD), Universiti Sains Malaysia, Penang, Malaysia; Institute of Research and Development (A Bali PhD, P Khoshvaght BSc), School of Engineering and Technology (Prof M Hosseinzadeh PhD), Institute for Global Health Innovations (C Nguyen MD, H T Nguyen MD), Duy Tan University, Da Nang, Viet Nam; Jadara Research Center (A Bali PhD), Jadara University, Irbid, Jordan; Faculty of Pharmacy (M Bashir PhD), Universiti Sultan Zainal Abidin, Terengganu, Malaysia; Department of Health Behaviour, Environment and Social Medicine (B Bintoro PhD), Center of Health and Behavior and Promotion (B Bintoro PhD), Universitas Gadjah Mada, Sleman, Indonesia; Department of Clinical Pharmacy (A N Bitar PhD), Universiti Sultan Zainal Abidin, Besut, Malaysia; School of Medicine (A C M Bui MD), University of Medicine and Pharmacy at Ho Chi Minh City, Ho Chi Minh, Viet Nam; School of Nursing (A Burhan MSc), Universitas Harapan Bangsa (National Hope University), Banyumas, Indonesia; School of Pharmaceutical Sciences (M Butt PhD), Universiti Sains Malaysia, Pulau

Penang, Malaysia; School of Medicine (D A Camporedondo MD), Caraga State University, Butuan City, Philippines; Elisa R. Ochoa Memorial Maternity and General Hospital, Butuan City, Philippines (D A Camporedondo MD); Faculty of Medicine (H Chen MSc), University of Cyberjaya, Cyberjaya, Malaysia; Office of Global Curtin (H Chen MSc), Curtin University, Perth, WA, Australia; Department of Psychiatry (D Chong BSc), Department of Public Health and Primary Care (M Dalakoti MPH), University of Cambridge, Cambridge, England; Department of Primary Care Medicine (K Chong MS, J Jamaluddin MMed), Faculty of Medicine (M Noushad PhD), Universiti Malaya, Kuala Lumpur, Malaysia; School of Pharmaceutical Science (C M Christopher PhD), Science University of Malaysia, Gelugor, Malaysia; The Interdisciplinary Research Group on Biomedicine and Health (D Chu PhD), Faculty of Applied Sciences (D Chu PhD), VNU International School (VNUIS), Hanoi, Viet Nam; Department of Internal Medicine (P Danpanichkul MD), Texas Tech University, Lubbock, TX, USA; Department of Neurosurgery (B G de Liyis MD), National Brain Center in Indonesia, Jakarta, Indonesia; Memorial Sloan Kettering Cancer Center (E Dee MD), Memorial Sloan Kettering Cancer Center, New York, NY, USA; Department of Computer Engineering (S Din PhD), Gachon University, Seongnam-si, South Korea; International Institute for Training and Research (INSTAR) (M N Do MD), Vietnam National University, Hanoi, Viet Nam; Department of Medicine (T H P Do MD), Department of Nutrition and Food Safety (H Nguyen PhD), Can Tho University of Medicine and Pharmacy, Can Tho, Viet Nam; School of Population Health (M Duong PhD), University of New South Wales, Sydney, NSW, Australia; Ateneo Center for Research and Innovation (A C Dy MD), Center for Research and Innovation (A Loreche MS), Ateneo De Manila University, Pasig City, Philippines; Demography and Health (A C Dy MD), London School of Hygiene & Tropical Medicine, London, England; Advanced Nursing Department (Prof F Efendi PhD), Division of Gastroenterohepatology (Prof M Miftahussurur PhD), Department of Pharmacy Practice (A Nurhan PharmD, Prof C Tan PhD), Department of Health and Life Sciences (R A Prastiya PhD), Department of Pharmacy Practice, Faculty of Pharmacy, Universitas Airlangga (Y A Pratama MSc), Department of Biology (Prof H Purnobasuki PhD), Faculty of Nursing (N Qur'aniati PhD), Department of Engineering (R Yunardi PhD), Universitas Airlangga (Airlangga University), Surabaya, Indonesia; La Trobe University, Melbourne, VIC, Australia (Prof F Efendi PhD); Drug Delivery System Excellence Center (M Esa PhD), Prince of Songkla University Hat Yai, Hat Yai, Thailand; Discipline of Social and Administrative Pharmacy (M Farooqui PhD), Science University of Malaysia, Penang, Malaysia; Centre for Public Health, Equity and Human Flourishing (N K Fauk PhD), Torrens University Australia, Adelaide, SA, Australia; Institute of Resource Governance and Social Change, Kupang, Indonesia (N K Fauk PhD); Faculty of Pharmacy (Prof N K Fuloria PhD), Faculty of Pharmcy (S Fuloria PhD), AIMST University, Bedong, Malaysia; Department of Public Health (F S Ganancial MPH), China Medical University, Taichung, Taiwan; Department of Health Policy and Administration (Prof F B Garcia PhD), College of Public Health (E R Gregorio PhD), Department of Neurosciences (Prof R G Jamora PhD), National Institutes of Health (A Loreche MS), University of the Philippines Manila, Manila, Philippines; Department of Anatomy (A E Go MD), Cebu Institute of Medicine, Cebu City, Philippines; Sunway Biofunctional Molecules Discovery Centre (Prof B Goh PhD), School of Pharmacy (Prof L Ming PhD), Sunway University, Sunway City, Malaysia; Australian Research Centre in Complementary and Integrative Medicine (Prof B Goh PhD), University of Technology Sydney, Ultimo, NSW, Australia; Department of Hepato-Pancreatico-

Biliary Surgery (N Hai Nam PhD), Thong Nhat Hospital, Ho Chi Minh City, Viet Nam; Medical Research Unit (H Harapan PhD), Universitas Syiah Kuala, Banda Aceh, Indonesia; Department of Epidemiology, Biostatistics, Population Studies and Health Promotion (A Hargono Dr), Faculty of Public Health, Universitas Airlangga, Surabaya, Indonesia; Health Polytechnic Ministry of Health Jakarta, Jakarta, Indonesia (A M A Hariandja DrPH); College of Medicine (Z Hein PhD), Ajman University, Ajman, United Arab Emirates; Department of Nursing (R Hikmat MSc), Department of Family and Preventive Medicine (Prof A Wattanapisit PhD), Prince of Songkla University, Songkhla, Thailand; Department of Cardiology (A Ho MSc), National University Hospital Singapore, Singapore, Singapore; Epidemiological Department (T Ho Huu PhD), Public Health Institute, Ho Chi Minh, Viet Nam; International Center for Materials Sciences and Technology (Prof M Hosseinzadeh PhD), Western Caspian University, Baku, Azerbaijan; Pediatric Nursing Department (M H Huda PhD), Faculty of Public Health (D Kusuma DSc, Prof I Trihandini PhD), University of Indonesia, Depok, Indonesia; Department of Antropology and Sociology (F Husain PhD), Department of Public Health (L Muzaqi MOHS), Universitas Negeri Semarang, Semarang, Indonesia; Department of Medical and Health Professional Education (M Ibrahim PhD), Faculty of Medicine (Prof M Leong Bin Abdullah DrPsych), Universiti Sultan Zainal Abidin, Kuala Terengganu, Malaysia; Faculty of Pharmacy (U I Ibrahim PhD), Sultan Zainal Abidin University, Malaysia, Terengganu, Malaysia; Department of Microbiology (K Imwattana PhD), Faculty of Nursing (S Ruksakulpiwat PhD), Excellence Center for Organ Transplantation (E Tantisattamo MD), Faculty of Public Health (U Wongsin PhD), Mahidol University, Bangkok, Thailand; Department of Pathology & Immunology (K Imwattana PhD), Baylor College of Medicine, Houston, TX, USA; Faculty of Pharmacy (L M Irham PhD), Universitas Ahmad Dahlan, Yogyakarta, Indonesia; Department of Environmental Health (F Islam MPH), Poltekkes Kemenkes Mamuju (Health Polytechnic of the Ministry of Health, Mamuju), Mamuju, Indonesia; Institute for Neurosciences (Prof R G Jamora PhD), St. Luke's Medical Center, Bonifacio Global City, Philippines; Department of Pharmacology and Life Sciences (H Jayasingh Chellammal PhD), Universiti Teknologi MARA (UiTM), Selangor, Malaysia; Centre for Evidence Synthesis and Implementation Research (Y A Jayasinghe MSc), Cephas Health Research Initiative, Inc, Ibadan, Nigeria; Rothschild Foundation Hospital (Prof J B Jonas MD), Rothschild Foundation Hospital, Paris, France; Singapore Eye Research Institute (Prof J B Jonas MD), Singapore Eye Research Institute, Singapore, Singapore; Riphah School of Computing & Innovation (Prof M Khan PhD), Riphah International University, Lahore, Pakistan; Department of CSE–AI&ML (P Khoshvaght BSc), Geethanjali College of Engineering and Technology, Hyderabad, India; Faculty of Health Sciences (S Khuzaiyah PhD), Universitas Muhammadiyah Pekajangan Pekalongan, Pekalongan, Indonesia; Graduate School of Biomedical and Health Sciences (K Ko PhD), Hiroshima University, Hiroshima, Japan; Independent Consultant, Jakarta, Indonesia (S Kosen MD); School of Applied Science (C Kua PhD), Republic Polytechnic, Singapore, Singapore; School of Health Sciences (Prof G Kuan PhD), Universiti Sains Malaysia, Kubang Kerian, Malaysia; School of Physical Education (Prof G Kuan PhD), Henan University, Keifeng, China; Department of Biostatistics and Research Methodology (Prof Y Kueh PhD), School of Health Sciences (S Shaharudin PhD), Universiti Sains Malaysia, Kota Bharu, Malaysia; Faculty of Medicine and Health Science (M Kurniasari PhD), Universitas Kristen Satya Wacana (Satya Wacana Christian University), Salatiga, Indonesia; School of Nursing (M

Kurniasari PhD), School of Pharmacy (A Nurhan PharmD), College of Medicine (N Viet-Nhi PhD), Graduate Institute of Injury Prevention and Control, College of Public Health (A F Widodo PhD), Taipei Medical University, Taipei, Taiwan; Clinical Research Unit of National Center for Mental Health Marzoeki Mahdi Hospital (A Kusuma DrPH), Ministry of Health of the Republic of Indonesia, Bogor, Indonesia; Department of Public Health and Epidemiology (D Kusuma DSc), Khalifa University of Science and Technology, Abu Dhabi, United Arab Emirates; School of Digital Science (D Lai PhD), Institute of Applied Data Analytics (D Lai PhD), Faculty of Science (E Leong PhD), Institute of Health Sciences (S Ong FAMS), Universiti Brunei Darussalam, Bandar Seri Begawan, Brunei; Department of Public Health and Nutrition (A D Laksono DrPH), National Research and Innovation Agency Republic of Indonesia, Central Jakarta, Indonesia; Health Policy (A D Laksono DrPH), Airlangga Centre for Health Policy, Surabaya, Indonesia; Department of Physiotherapy (T Laksono PhD), Universitas 'Aisyiyah Yogyakarta, Yogyakarta, Indonesia; School of Medicine (B Le MD), Pham Ngoc Thach University of Medicine, Ho Chi Minh, Viet Nam; Department of Internal Medicine (T H Tran MD), University of Medicine and Pharmacy at Ho Chi Minh City, Ho Chi Minh City, Viet Nam (T T T Le MD); Faculty of Electronic Engineering and Technology (H Lee PhD), Universiti Malaysia Perlis, Arau, Malaysia; School of Pharmacy (Prof S W H Lee PhD), Jeffrey Cheah School of Medicine and Health Sciences (Prof R R Marzo MD), Monash University, Subang Jaya, Malaysia; School of Pharmacy (Prof S W H Lee PhD), Taylor's University Lakeside Campus, Subang Jaya, Malaysia; Department of Psycho-oncology (Prof M Leong Bin Abdullah DrPsych), First Affiliated Hospital of Henan Medical University, Weihui City, China; Faculty of Health Sciences (R Lestari Dr), Department of Public Health (L Zuhriyah PhD), Universitas Brawijaya, Malang, Indonesia; Division or Respiratory & Critical Care Medicine (M Liew MRCP), Alexandra Hospital, Singapore, Singapore; Department of Medicine (L Lim PhD), Department of Sports Medicine (Z See MS), University of Malaya, Kuala Lumpur, Malaysia; Department of Medicine and Therapeutics (L Lim PhD), The Chinese University of Hong Kong, Hong Kong, China; International College of Digital Innovation (D Liu MS), Chiang Mai University, Chiang mai, Thailand; Department of Cardiovascular Metabolism Diseases (X Liu MD), Health Services and Systems Research Programme (S Sarawagi MSc), Duke-NUS Medical School, Singapore, Singapore; Dr. Mary and Ron Neal Cancer Center (Prof H N Luu MD), Houston Methodist Research Institute, Houston, TX, USA; School of Human and Health Sciences (K Lwin PhD), University of Huddersfield, Huddersfield, UK; Centre for Public Health and Wellbeing (Z Ma PhD), University of the West of England, Bristol, England; Heidelberg Institute of Global Health (M Mahanani PhD), Heidelberg University, Heidelberg, Germany; Division Prevention of Cardiovascular and Metabolic Diseases (M Mahanani PhD), Department of Cardiology, Angiology, Haemostaseology and Medical Intensive Care (Y Wibowo Dr), Department of Experimental Pharmacology (Y Wibowo Dr), Heidelberg University, Mannheim, Germany; Faculty of Humanities and Health Sciences (Prof R R Marzo MD), Curtin University, Sarawak, Malaysia; Department of Epidemiology (Prof R J Maude PhD), Mahidol-Oxford Tropical Medicine Research Unit, Bangkok, Thailand; Nuffield Department of Medicine (Prof R J Maude PhD), Radcliffe Department of Medicine (T Vinh BS), University of Oxford, Oxford, England; Research Center for Public Health and Nutrition (M Mikrajab MPH), National Research and Innovation Agency (BRIN), Bogor District, Indonesia; Centre for Aquatic Foods and Global Health (AFAGH), Institute of Tropical

Aquaculture and Fisheries (AKUATROP) (Prof M Moradi-Lakeh MD), Universiti Malaysia Terengganu, Kuala Terengganu, Malaysia; Preventive Medicine and Public Health Research Center (Prof M Moradi-Lakeh MD), Iran University of Medical Sciences, Tehran, Iran; Department of Medicine (A Mukhopadhyay MD), National University Health System, Singapore, Singapore; Department of Epidemiology (K P Nagdeo DDS), New York University, New York, NY, USA; Faculty of Pharmacy (Prof F Nainu PhD), Hasanuddin University, Makassar, Indonesia; Faculty of Pharmacy (T T Ngo MSc), Ton Duc Thang University, Hanoi, Viet Nam; Department of Health Economics and Policy (H T Nguyen PhD), Department of Epidemiology (Prof L T H Vu PhD), Hanoi University of Public Health, Hanoi, Viet Nam; Institute for Advanced Study in Technology (L H Nguyen PhD), Ton Duc Thang University, Ho Chi Minh city, Viet Nam; Department of Epidemiology (M T Nguyen PhD), University of Medicine and Pharmacy at Ho Chi Minh City, Ho chi Minh, Viet Nam; Department of Epidemiology (M Nguyen MPharm), Vanderbilt University, Nashville, TN, USA; Cardiovascular Research Department (N P Nguyen MD), Methodist Hospital, Merrillville, IN, USA; College of Health Sciences (N P Nguyen MD), Nam Can Tho University, Can Tho City, Viet Nam; The Children's Hospital Westmead Clinical School (T Nguyen MD), The University of Sydney, New South Wales, NSW, Australia; International University of Health and Welfare (T G Nguyen MD), International University of Health and Welfare, Narita, Japan; Stockholm Resilience Center (L Nguyen Thanh PhD), Stockholm University, Stockholm, Sweden; College of Health Science (C T Nguyen Thi MPH), College of Health Sciences (T T Pham PhD), College of Health Sciences (CHS) (Prof D Poddighe PhD), VinUniversity, Hanoi, Viet Nam; Faculty of Medicine (R Nindrea PhD), Universitas Negeri Padang in Indonesia, Padang, Indonesia; Midwifery Department (N Nurfatimah MPH), Poltekkes Kemenkes Palu, Palu, Indonesia; Department of Public Health (S Ong FAMS), Ministry of Health, Bandar Seri Begawan, Brunei; Department of Global Health and Social Medicine (S Onie PhD), Joslin Diabetes Center (B Tagiisuran PhD, S Tye PhD), Harvard University, Boston, MA, USA; Wellspring Research (S Onie PhD), Wellspring Center Indonesia, Jakarta, Indonesia; National Research and Innovation Agency (H U Pangaribuan MSc), National Research Council, Jakarta, Indonesia; College of Nursing (S M Pangaribuan MSc), Krida Wacana Christian University, Jakarta, Indonesia, Taipei, Indonesia; Research Center for Public Health and Nutrition (B A Paramashanti PhD), National Research and Innovation Agency (BRIN) in Indonesia, Jakarta, Indonesia; St Luke's Medical Center Foundation, Inc, Taguig City, Philippines (V F Pepito MSc); Research Advancement Consortium in Health, Hanoi, Viet Nam (T T Pham PhD); Department of Cardiology (Prof K Poh MD), National University Heart Center Singapore, Singapore, Singapore; Research Center in Advancing Community Healthcare, Surabaya, Indonesia (N Qur'aniati PhD); School of Pharmaceutical Sciences (A Qureshi PhD), Universiti Sains Malaysia in Malaysia, Penang, Malaysia; Research Center for Public Health and Nutrition (B Rachmat MPH), National Research and Innovation Agency (BRIN), Jakarta, Indonesia (B Roosihermiatie PhD, I U Tarigan PhD); Chakri Naruebodindra Medical Institute (A Rahman PhD), Mahidol University, Samut Prakan, Thailand; Preclinical Department (V Ramachandran PhD), Royal College of Medicine Perak, Universiti Kuala Lumpur, Ipoh, Malaysia; Department of Midwifery (K Ramadhan MPH), Ministry of Health of the Republic of Indonesia, Palu, Indonesia; School of Nursing & Health Sciences (S Ramazanu PhD), Hong Kong Metropolitan University, Hong Kong, China; Computer Science and Engineering Department (Prof A Rehman PhD), Saveetha Institute of

Medical and Technical Sciences, Chennai, India; College of Nursing (Y Rias MNS), Insitut Ilmu Kesehatan Bhakti Wiyata Kediri, Kediri, Indonesia; School of Nursing (Prof E Rochmawati PhD), Universitas Muhammadiyah Yogyakarta, Bantul, Indonesia; School of Allied Health (Prof A Rodriguez PhD), Anglia Ruskin University, Chelmsford, England; Bristol Heart Institute (T Rojanathagoon MD), University of Bristol, Bristol, England; Department of Population Health (Prof C Rojanaworarit PhD), Hofstra University, Hempstead, NY, USA; Faculty of Medicine (B Roy PhD), Quest International University Perak, Ipoh, Malaysia; Department of Smart Computing and Cyber Resilience (DSCCR) (A Sahban Rafsanjani PhD), Sunway University, Selangor, Malaysia; Institute of Epidemiology and Preventive Medicine (Y L Samodra PhD), National Taiwan University, Taipei, Taiwan; Benang Merah Research Center (BMRC), Minahasa Utara, Indonesia (Y L Samodra PhD); National Dental Research Institute Singapore (S Sarawagi MSc), National Dental Centre Singapore, Singapore, Singapore; Department of Palliative Nursing (N Sari MN), Widya Mandala Surabaya Catholic University in Indonesia, Surabaya, Indonesia; School of Computer Sciences (M Sarvghadi PhD), University of Science Malaysia, Penang Island, Malaysia; Department of Applied Social Sciences (T Sawn Khai PhD), Hong Kong Polytechnic University, Hong Kong, China; School of Chemical Sciences (A Sayaf PhD), University Sains Malaysia, Penang, Malaysia; Clinical Trials Unit (K Seah PhD), University of Warwick, Coventry, England; Faculty of Dentistry (Prof S Selvaraj PhD), University of Puthisastra, Phnom Penh, Cambodia; Department of Internal Medicine (R Sinto MD), University of Indonesia, Jakarta, Indonesia; Department of Internal Medicine (R Sinto MD), Dr. Cipto Mangunkusumo National Hospital, Jakarta Pusat, Indonesia; Department of Medical Sciences (Prof V Subramaniyan PhD), Sunway University, Subang Jaya, Malaysia; Faculty of Medical and Life Science (C Sui MSc), Sunway University, Kuala Lumpur, Malaysia; Faculty of Health Science (D Sulistiyorini MSc), Universitas Indonesia Maju, Jakarta, Indonesia; Infectious and Tropical Diseases Epidemiology, Public Health Program (H Surendra PhD), Monash University, Tengerang, Indonesia; Oxford University Clinical Research Unit Indonesia (H Surendra PhD), Oxford University, Jakarta, Indonesia; University of Dian Nuswantoro, Semarang, Indonesia (S Suryanti PhD); Faculty of Nursing (H Susanto PhD), Universitas Islam Sultan Agung, Semarang, Indonesia; Department of Clinical Pathology (H Susianti PhD), Brawijaya University, Malang, Indonesia; Hospital Central Laboratory (H Susianti PhD), Dr Saiful Anwar General Hospital, Malang, Indonesia; Department of Microbiology (T N Susilawati PhD), Universitas Sebelas Maret, Surakarta, Indonesia; Faculty of Public Health (D A Syaiful MGPH), Universitas Airlangga Indonesia, Surabaya, Indonesia; School of Pharmaceutical Sciences (B Tagiisuran PhD), University of Science Malaysia, Penang, Malaysia; School of Pharmacy (Prof C Tan PhD), KPJ Healthcare University, Negeri Seremban, Malaysia; American Heart Association Comprehensive Hypertension Center (E Tantisattamo MD), University of California Irvine, Orange, CA, USA; College of Health Sciences (Prof A W Taylor-Robinson PhD), VinUniversity (VinUni), Hanoi, Vietnam, Hanoi, Viet Nam; Center for Global Health (Prof A W Taylor-Robinson PhD), University of Pennsylvania, Philadelphia, PA, USA; School of Biomedical Sciences (V Thai PhD), The University of Western Australia, Crawley, WA, Australia; University of Cyberjaya, Selangor, Malaysia (Prof L Thangavelu PhD); Faculty of Public Health (J H V Ticoalu MPH), Universitas Sam Ratulangi (Sam Ratulangi University), Manado, Indonesia; College of Nursing (S I Tonapa PhD), Kaohsiung Medical University, Taiwan, Kaohsiung, Taiwan; School of Biomedical

Engineering (N M Tran PhD), International University, VNU-HCM, Ho Chi Minh city, Viet Nam; School of Biomedical Engineering (N M Tran PhD), Viet Nam National University, Ho Chi Minh City, Viet Nam; International Center (N Tran PhD), Tokushima University, Tokushima, Japan; Second Department of Internal Medicine (Q T H Tran PhD), Kansai Medical University, Hirakata, Japan; Department of Business Analytics (T H Tran MD), University of Massachusetts Dartmouth, Dartmouth, MA, USA; Department of Internal Medicine (T Tran MSc), Minh Anh Hospital, Ho Chi Minh, Viet Nam; Research and Advocacy Initiative (N Tran Minh Duc MD), ALS Vietnam, Quang Ngai, Viet Nam; College of Health Science (Q X N Truong PhD), Vin University, Hanoi, Viet Nam; Department of Occupational Health and Safety (A R Tualeka PhD), University of Development, Surabaya, Indonesia; Department of Family Medicine & Population Health (Prof J van Olmen PhD), University of Antwerp, Antwerp, Belgium; Raffles Neuroscience Centre (Prof N Venketasubramanian MSc), Raffles Hospital, Singapore, Singapore; Faculty of Public Health (L Vu PhD), International Institute for Training and Research (INSTAR) (L Vu PhD), VNU University of Medicine and Pharmacy, Hanoi, Viet Nam; Department of Health and Human Performance (Prof T Vu PhD), York College/City University of New York, New York, NY, USA; School of Business (Prof A Wagdy PhD), University of Science and Technology, 6th of October City, Egypt; Department of Physical Therapy (T Wiangkham PhD), Naresuan University, Phitsanulok, Thailand; Department of Nursing (A Wilandika PhD), Universitas Aisyiyah Bandung, Bandung, Indonesia; Faculty of Public Health (S Winarni DrPH), Diponegoro University, Kota Semarang, Indonesia; Universiti Sains Malaysia (M Yam PhD), Universiti Sains Malaysia, Pulau Pinang, Malaysia; ASEAN Institute for Health Development (K Yang PhD), Mahidol University, Nakhon Pathom, Thailand; KHANA Center for Population Health Research, Phnom Penh, Cambodia (Prof S Yi PhD); Department of Health Policy and Management (Prof M Younis PhD), Jackson State University, Jackson, MS, USA; Asia Pacific University of Technology & Innovation, Kuala Lumpur, Malaysia (Prof M Younis PhD)

Authors' contributions

Providing data or critical feedback on data sources

Hanif Abdul Rahman, Asnawi Abdullah, Michael R M Abrigo, Tawseef Ahmad, Budi Aji, Ali Salman Al-Shami, Mohammed Alfaqeeh, Syed Mohamed Aljunid, Joseph Uy Almazan, Junaid Amin, Nguyen Hzoang Anh, Sumadi Lukman Anwar, Mohammad Ashfaq, Muhammad Shahzad Aslam, Yuni Asri, Zaw Zaw Aung, Zulfa Ayuningsih, Fitriyono Ayustaningwarno, Mohd Yusmaidie Aziz, Ahmed Bali, Muhammad Irfan Bashir, Bagas Suryo Bintoro, Ahmad Naoras Bitar, Asmat Burhan, Muhammad Daoud Butt, Dimples Acquiatan Camporedondo, Periklis Charalampous, Nicholas WS Chew, Ruixin Chi, Bryan Chong, Christina Malini Christopher, Dinh-Toi Chu, Mayank Dalakoti, Pojsakorn Danpanichkul, Bryan Gervais de Liyis, Edward Christopher Dee, Sadia Din, Mai Ngoc Do, Thao Huynh Phuong Do, Muhammad Esa, Maryam Farooqui, Neeraj Kumar Fuloria, Shivkanya Fuloria, Francis Sarial Ganancial, Adrian Eleazar Go, Ernesto Ramos Gregorio, Nguyen Hai Nam, Harapan Harapan, Andy Martahan Andreas Hariandja, Faizul Hasan, Simon I Hay, Zaw Myo Hein, Rohman Hikmat, Mehdi Hosseinzadeh, Lalu Muhammad Irham, Jazlan Jamaluddin, Hanish Singh Jayasingh Chellammal, Yovanthi Anurangi Jayasinghe, Jost B Jonas, Muhammad Khan, Parisa Khoshvaght, Soewarta Kosen, Garry Kuan, Maria Dyah Kurniasari, Asep Kusnali, Dian Kusuma, Thao Thi Thu Le, Hoi Leong Lee, Shaun Wen Huey Lee, Xiao Liu, Kaung Suu Lwin, Zheng Feei Ma, Roy Rillera Marzo, Amartya Mukhopadhyay, Lutfi Muzaqi, Mohsen Naghavi, Tam Thi Ngo, Cao Duy Nguyen, Ha Thu Nguyen, Hien Thu Nguyen, Long Hoang Nguyen, Minh-Ha Nguyen, Nghia Phu Nguyen, Thanh Van Nguyen, Thinh Gia Nguyen, Nurfatimah Nurfatimah, Ahmad Dzulfikri Nurhan, Sok King Ong, Sandersan Onie, Santa Maria Pangaribuan, Thantrira Porntaveetus, Nuzul Qur'aniati, Vignesh Ramachandran, Kadar Ramadhan, Sheena Ramazanu, Ateeq Rehman, Yohanes Andy Rias, Thanakorn Rojanathagoon, Chanapong Rojanaworarit, Debby Syahru Romadlon, Betty Roosihermiatie, Suebsarn Ruksakulpiwat, Mohammad-Ali Sarvghadi, Tual Sawn Khai, Abrar Mohammad Sayaf, KT Matthew Seah, Siddharthan Selvaraj, Shazlin Shaharudin, JiaHao Shi, Ching-Hui Sia, Chandrashekhar T Sreeramareddy, Vetriselvan Subramaniyan, Chee Fai Sui, Henry Surendra, Suryanti Suryanti, Tri Nugraha Susilawati, Dayinta Annisa Syaiful, Ching Siang Tan, Kit-Aun Tan, Ekamol Tantisattamo, Van Chi Thai, Hein Thu, Santo Imanuel Tonapa, Quynh Thuy Huong Tran, Thi Thuy Vy Tran, Indang Trihandini, Abdul Rohim Tualeka, Narayanaswamy Venketasubramanian, Nguyen-Kieu Viet-Nhi, Tuan Vinh, Linh Vu, Ali Wagdy, Mugi Wahidin, Taweewat Wiangkham, Akhmad Fajri Widodo, Angga Wilandika, Utoomporn Wongsin, Siyan Yi, Mustafa Younis, and Emilia Zainal Abidin.

Developing methods or computational machinery

Simon I Hay and Mohsen Naghavi.

Providing critical feedback on methods or results

Jeofrey Bautista Abalos, Edimansyah Abdin, Hanif Abdul Rahman, Asnawi Abdullah, Abdulrakib Abdulrahim, Michael R M Abrigo, Wirawan Adikusuma, Tawseef Ahmad, Sima Ahmadpour, Mohamed Mustaf Ahmed, Budi Aji, Ali Salman Al-Shami, Mohammed Alfaqeeh, Khaidar Ali, Syed

Mohamed Aljunid, Joseph Uy Almazan, Junaid Amin, Roshan A Ananda, Chin-Siang Ang, Nguyen Hoang Anh, Sumadi Lukman Anwar, Rizal Arifin, Keivan Armani, Mohammad Ashfaq, Muhammad Shahzad Aslam, Yuni Asri, Zaw Zaw Aung, Zulfa Ayuningsih, Fitriyono Ayustaningwarno, Abul Kalam Azad, Mohd Yusmaidie Aziz, Ahmed Bali, Muhammad Irfan Bashir, Bagas Suryo Bintoro, Ahmad Naoras Bitar, Ai Cao My Bui, Asmat Burhan, Muhammad Daoud Butt, Dimples Acquiatan Camporedondo, Yu Cao, Periklis Charalampous, Hana Chen, Nicholas WS Chew, Ruixin Chi, Bryan Chong, Dennis Chong, Karleen Chong, Christina Malini Christopher, Dinh-Toi Chu, Mayank Dalakoti, Pojsakorn Danpanichkul, Bryan Gervais de Liyis, Edward Christopher Dee, Sadia Din, Mai Ngoc Do, Thao Huynh Phuong Do, Minh Cuong Duong, Angel Belle Cheng Dy, Ferry Efendi, Muhammad Esa, Maryam Farooqui, Neeraj Kumar Fuloria, Shivkanya Fuloria, Francis Sarial Ganancial, Fernando Jr Barroga Garcia, Adrian Eleazar Go, Bey Hing Goh, Ernesto Ramos Gregorio, Nguyen Hai Nam, Harapan Harapan, Arief Hargono, Faizul Hasan, Simon I Hay, Zaw Myo Hein, Rohman Hikmat, Aaron Kwun Hang Ho, Tinh Ho Huu, Mehdi Hosseinzadeh, Mega Hasanul Huda, Fadly Husain, Mohd Salami Ibrahim, Umar Idris Ibrahim, Korakrit Imwattana, Lalu Muhammad Irham, Jazlan Jamaluddin, Roland Dominic G Jamora, Hanish Singh Jayasingh Chellammal, Yovanthi Anurangi Jayasinghe, Jost B Jonas, Muhammad Khan, Parisa Khoshvaght, Siti Khuzaiyah, Ko Ko, Chong-Han Kua, Garry Kuan, Yee Cheng Kueh, Maria Dyah Kurniasari, Dea Anita Ariani Kurniasih, Asep Kusnali, Aria Kusuma, Dian Kusuma, Daphne Teck Ching Lai, Tri Laksono, Jerrald Lau, Bao Huy Le, Thao Thi Thu Le, Hoi Leong Lee, Shaun Wen Huey Lee, Elvynna Leong, Mohammad Farris Iman Leong Bin Abdullah, Retno Lestari, Mei Fong Liew, Lee-Ling Lim, Donglin Liu, Xiao Liu, Arianna Maever Loreche, Hung N Luu, Kaung Suu Lwin, Zheng Feei Ma, Melani Ratih Mahanani, Roy Rillera Marzo, Richard James Maude, Muhammad Miftahussurur, Long Chiau Ming, Maziar Moradi-Lakeh, Amartya Mukhopadhyay, Lutfi Muzaqi, Kiran P Nagdeo, Mohsen Naghavi, Firzan Nainu, Tam Thi Ngo, Cao Duy Nguyen, Ha Thu Nguyen, Hien Thu Nguyen, Hien Thi Nguyen, Long Hoang Nguyen, Minh Thien Nguyen, Minh-Ha Nguyen, Nghia Phu Nguyen, Thanh Van Nguyen, Thinh Gia Nguyen, Luong Nguyen Thanh, Cham Thi Nguyen Thi, Ricvan Dana Nindrea, Nurfatimah Nurfatimah, Ahmad Dzulfikri Nurhan, Sandersan Onie, Zhinya Kawa Othman, Helena Ullyartha Pangaribuan, Santa Maria Pangaribuan, Bunga A Paramashanti, Veincent Christian Filipino Pepito, Tung Thanh Pham, Kian Keong Poh, Thantrira Porntaveetus, Ragil Angga Prastiya, Yusuf Alif Pratama, Hery Purnobasuki, Nuzul Qur'aniati, Ali Qureshi, Basuki Rachmat, Asad Ur Rahman, Vignesh Ramachandran, Kadar Ramadhan, Ateeq Rehman, Yohanes Andy Rias, Erna Rochmawati, Alina Rodriguez, Iftitakhur Rohmah, Thanakorn Rojanathagoon, Chanapong Rojanaworarit, Debby Syahru Romadlon, Betty Roosihermiatie, Suebsarn Ruksakulpiwat, Ahmad Sahban Rafsanjani, Shuaibu Musa Saidu, Yoseph Leonardo Samodra, Lintang Dian Saraswati, Shilpa Sarawagi, Ni Putu Wulan Purnama Sari, Tual Sawn Khai, Abrar Mohammad Sayaf, KT Matthew Seah, Zhi Huang See, Siddharthan Selvaraj, Shazlin Shaharudin, JiaHao Shi, Ching-Hui Sia, Robert Sinto, Chandrashekhar T Sreeramareddy, Vetriselvan Subramaniyan, Chee Fai Sui, Henry Surendra, Suryanti Suryanti, Herry Susanto, Hani Susianti, Tri Nugraha Susilawati, Dayinta Annisa Syaiful, Balamurugan Tagiisuran, Ching Siang Tan, Kit-Aun Tan, Ekamol Tantisattamo, Ingan Ukur Tarigan, Andrew W Taylor-Robinson, Van Chi Thai, Lakshmi Thangavelu, Hein Thu, Jansje Henny Vera Ticoalu, Santo Imanuel Tonapa, Nam Minh-phuong Tran, Nam Hoang Tran, Quynh Thuy Huong Tran, Thi Thuy Vy

Tran, Nguyen Tran Minh Duc, Indang Trihandini, Quynh Xuan Nguyen Truong, Sok Cin Tye, Stephanie C C van der Lubbe, Narayanaswamy Venketasubramanian, Nguyen-Kieu Viet-Nhi, Tuan Vinh, Lan Thi Hoang Vu, Linh Vu, Thinh Toan Vu, Ali Wagdy, Mugi Wahidin, Apichai Wattanapisit, Taweewat Wiangkham, Yohanes Cakrapradipta Wibowo, Akhmad Fajri Widodo, Angga Wilandika, Sri Winarni, Utoomporn Wongsin, Mun Fei Yam, Kwang Mo Yang, Siyan Yi, Mustafa Younis, Riky Tri Yunardi, Emilia Zainal Abidin, and Lilik Zuhriyah.

Drafting the manuscript or revising it critically for important intellectual content

Hanif Abdul Rahman, Asnawi Abdullah, Abdulrakib Abdulrahim, Tawseef Ahmad, Sima Ahmadpour, Ali Salman Al-Shami, Mohammed Alfaqeeh, Khaidar Ali, Syed Mohamed Aljunid, Junaid Amin, Roshan A Ananda, Chin-Siang Ang, Irfan Ardani, Muhammad Shahzad Aslam, Yuni Asri, Zulfa Ayuningsih, Fitriyono Ayustaningwarno, Ahmed Bali, Muhammad Irfan Bashir, Ai Cao My Bui, Asmat Burhan, Dimples Acquiatan Camporedondo, Periklis Charalampous, Hana Chen, Nicholas WS Chew, Ruixin Chi, Bryan Chong, Dinh-Toi Chu, Mayank Dalakoti, Bryan Gervais de Liyis, Edward Christopher Dee, Sadia Din, Mai Ngoc Do, Minh Cuong Duong, Ferry Efendi, Ekawati Ekawati, Muhammad Esa, Maryam Farooqui, Nelsensius Klau Fauk, Neeraj Kumar Fuloria, Shivkanya Fuloria, Fernando Jr Barroga Garcia, Bey Hing Goh, Ernesto Ramos Gregorio, Nguyen Hai Nam, Harapan Harapan, Faizul Hasan, Simon I Hay, Zaw Myo Hein, Rohman Hikmat, Mehdi Hosseinzadeh, Mohd Salami Ibrahim, Umar Idris Ibrahim, Lalu Muhammad Irham, Fahrul Islam, Jazlan Jamaluddin, Yovanthi Anurangi Jayasinghe, Jost B Jonas, Muhammad Khan, Parisa Khoshvaght, Chong-Han Kua, Maria Dyah Kurniasari, Asep Kusnali, Aria Kusuma, Dian Kusuma, Daphne Teck Ching Lai, Agung Dwi Laksono, Bao Huy Le, Thao Thi Thu Le, Hoi Leong Lee, Shaun Wen Huey Lee, Elvynna Leong, Mohammad Farris Iman Leong Bin Abdullah, Taishun Li, Lee-Ling Lim, Donglin Liu, Xiao Liu, Kaung Suu Lwin, Zheng Feei Ma, Melani Ratih Mahanani, Roy Rillera Marzo, Muhammad Agus Mikrajab, Maziar Moradi-Lakeh, Amartya Mukhopadhyay, Lutfi Muzaqi, Mohsen Naghavi, Marie Ng, Cao Duy Nguyen, Ha Thu Nguyen, Hien Thu Nguyen, Minh Thien Nguyen, Nghia Phu Nguyen, Thanh Van Nguyen, Thinh Gia Nguyen, Cham Thi Nguyen Thi, Mohammed Noushad, Nurfatimah Nurfatimah, Sandersan Onie, Helena Ullyartha Pangaribuan, Bunga A Paramashanti, Veincent Christian Filipino Pepito, Dimitri Poddighe, Kian Keong Poh, Thantrira Porntaveetus, Nuzul Qur'aniati, Ali Qureshi, Vignesh Ramachandran, Kadar Ramadhan, Ateeq Rehman, Yohanes Andy Rias, Alina Rodriguez, Thanakorn Rojanathagoon, Chanapong Rojanaworarit, Debby Syahru Romadlon, Betty Roosihermiatie, Bedanta Roy, Suebsarn Ruksakulpiwat, Lintang Dian Saraswati, Shilpa Sarawagi, Tual Sawn Khai, Abrar Mohammad Sayaf, KT Matthew Seah, Zhi Huang See, Siddharthan Selvaraj, Shazlin Shaharudin, Ching-Hui Sia, Robert Sinto, Chandrashekhar T Sreeramareddy, Vetriselvan Subramaniyan, Chee Fai Sui, Desy Sulistiyorini, Henry Surendra, Herry Susanto, Tri Nugraha Susilawati, Balamurugan Tagiisuran, Ching Siang Tan, Kit-Aun Tan, Andrew W Taylor-Robinson, Santo Imanuel Tonapa, Nam Minh-phuong Tran, Nam Hoang Tran, Thang Huu Tran, Thi Thuy Vy Tran, Nguyen Tran Minh Duc, Sok Cin Tye, Stephanie C C van der Lubbe, Josefien van Olmen, Narayanaswamy Venketasubramanian, Nguyen-Kieu Viet-Nhi, Tuan Vinh, Linh Vu, Thinh Toan Vu, Ali Wagdy, Tati Suryati Warouw, Apichai

Wattanapisit, Taweewat Wiangkham, Sri Winarni, Utoomporn Wongsin, Kwang Mo Yang, Siyan Yi, Riky Tri Yunardi, Emilia Zainal Abidin, Cuilin Zhang, and Yawen Zhou.

Managing the estimation or publications process

Simon I Hay, Mohsen Naghavi, and Marie Ng.
